# Boryl radical-mediated halogen-atom transfer (XAT) enables the Sonogashira-like alkynylation of alkyl halides†

**DOI:** 10.1039/d4sc06516f

**Published:** 2024-10-24

**Authors:** Javier Corpas, Maialen Alonso, Daniele Leonori

**Affiliations:** a Institute of Organic Chemistry, RWTH Aachen University Landoltweg 1 Aachen 52056 Germany daniele.leonori@rwth-aachen.de

## Abstract

Alkynes are a crucial class of materials with application across the wide range of chemical disciplines. The alkynylation of alkyl halides presents an ideal strategy for assembling these materials. Current methods rely on the intrinsic electrophilic nature of alkyl halides to couple with nucleophilic acetylenic systems, but these methods faces limitations in terms of applicability and generality. Herein, we introduce a different approach to alkynylation of alkyl halides that proceeds *via* radical intermediates and uses alkynyl sulfones as coupling partners. This strategy exploits the ability of amine-ligated boryl radicals to activate alkyl iodides and bromides through halogen-atom transfer (XAT). The resulting radicals then undergo a cascade of α-addition and β-fragmentation with the sulfone reagent, leading to the construction of C(sp^3^)–C(sp) bonds. The generality of the methodology has been demonstrated by its successful application in the alkynylation of complex and high-value molecules.

## Abstract 2

The Sonogashira-like alkynylation of alkyl halides is achieved by a photocatalytic approach exploiting amine-ligated boryl radicals as halogen-atom transfer mediators in combination with alkynyl sulfones as SOMOphilic reagents.

## Graphical abstract

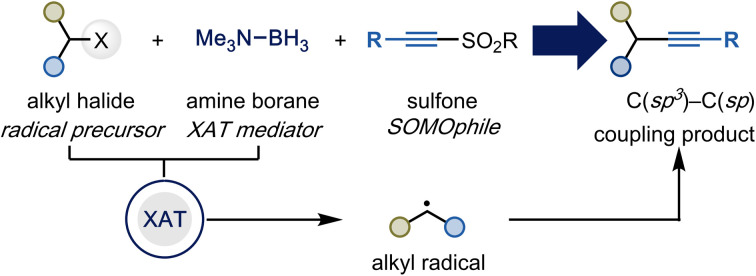

## Introduction

**Scheme 1:**
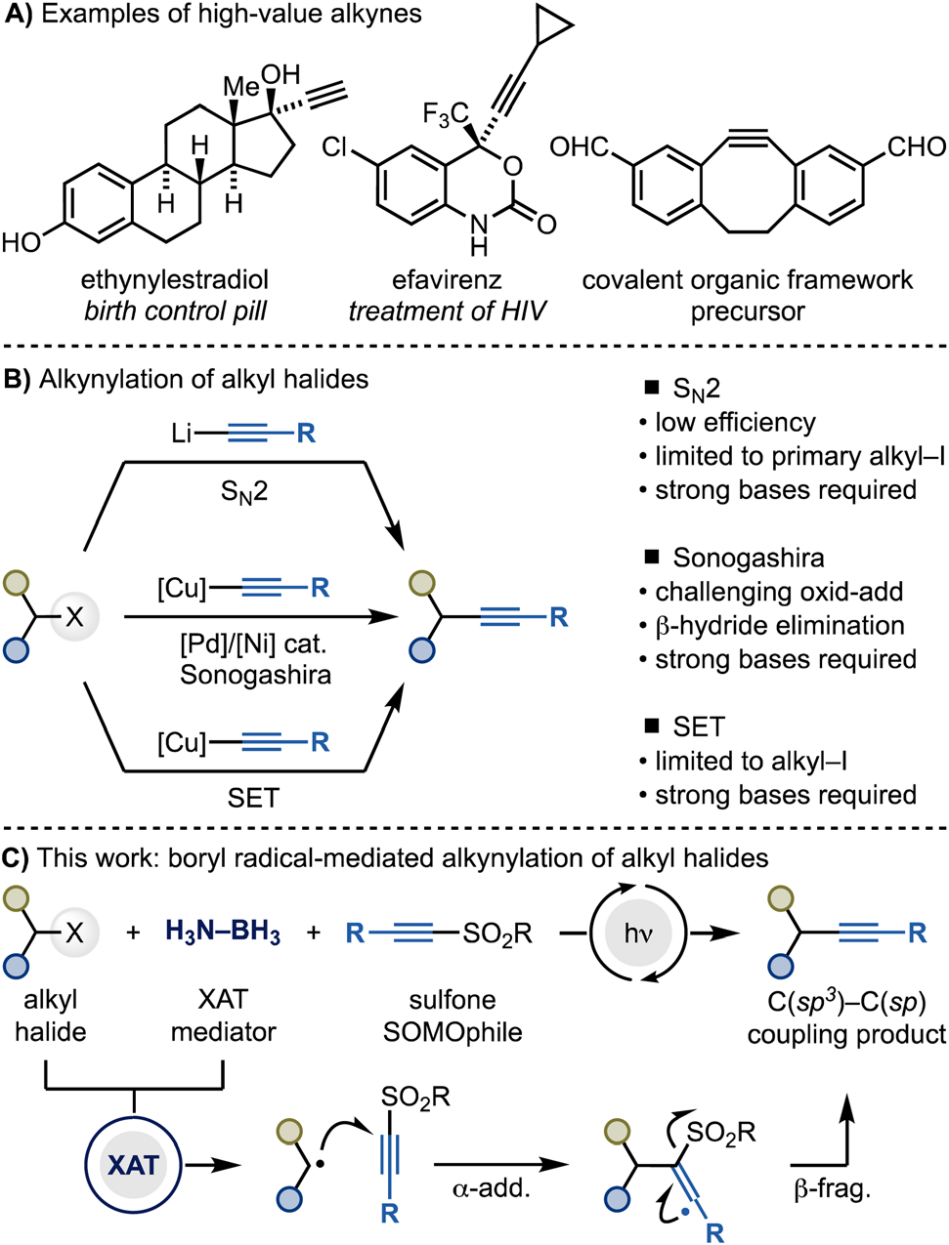
(A) Examples of high-value alkyne-containing materials. (B) General methods for the alkynylation of alkyl halides and their limitations. (C) This work demonstrates a radical approach based on XAT for the alkynylation of alkyl halides (iodides and bromides).

Alkynes play key roles in chemical synthesis, medicinal chemistry and materials science (Scheme 1A).^1,2^ For instance, alkynes are widely used as dipolarophiles in [3 + 2] cycloadditions, a pivotal transformation within the bioconjugation toolbox.^3^ Additionally, they are often employed as bioisosteres for various functionalities, such as carbonyls, 1,4-disubstituted phenyls, and cyclopropyl groups.^4^ Consequently, the development of methods for the modular introduction of alkyne functionalities onto organic molecules is still a highly sought-after goal in synthesis and catalysis.

Within this context, the alkynylation of alkyl halides would be particularly useful considering the large amounts of derivatives commercially available (Scheme 1B). However, engaging these species in C(sp^3^)–C(sp) bond formations is still synthetically challenging. Current methods match the intrinsic electrophilic nature of alkyl halides with nucleophilic acetylene sources for S_N_2 reactivity (*e.g.* alkynyl organolithiums), Sonogashira cross-couplings (*e.g.* alkynyl organocoppers) and radical manifolds (also alkynyl organocoppers). However, these strategies are often synthetically restricted to primary alkyl iodides due to challenges in either substitution reactivity (S_N_2 methods),^5^ oxidative addition and β-hydride elimination (Sonogashira cross-coupling)^6^ or C(sp^3^)–halogen bond reduction (radical methods).^7,8^

Herein, we demonstrate an alternative and general approach for C(sp^3^)–C(sp) bond formation of alkyl halides using alkynyl sulfones and Me_3_N–BH_3_ under photocatalytic conditions (Scheme 1C). This method exploits amine-ligated boryl radicals for the conversion of alkyl halides into the corresponding radicals, followed by their cascade α-addition–β-elimination reactions with the SOMOphilic sulfone reagent. This strategy enables the utilization of both alkyl iodides and bromides and provide a metal-free option to the synthesis of high-value alkyne materials.

### Reaction design

**Scheme 2:**
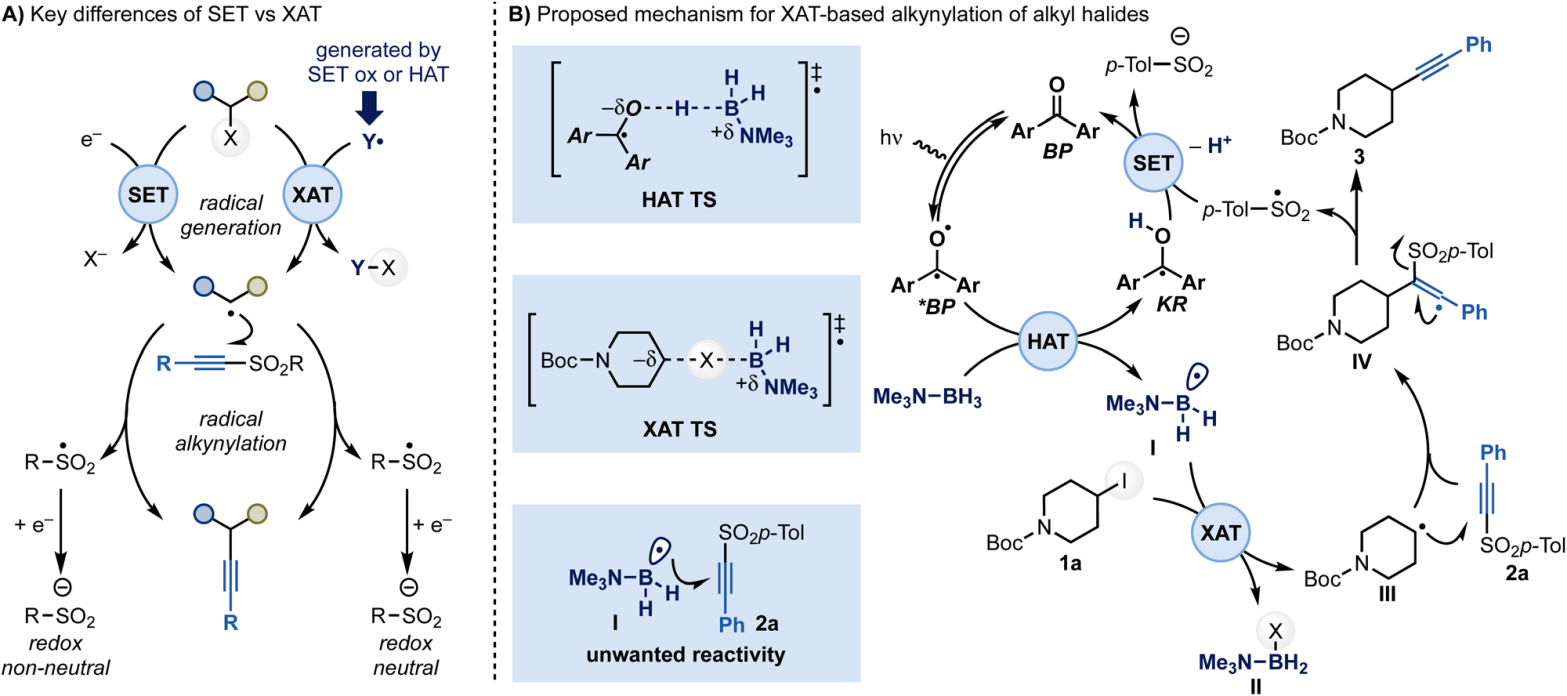
(A) Key mechanistic differences in the use of SET *vs.* XAT reactivity for radical alkynylation. (B) Proposed mechanism for the XAT-based alkynylation of alkyl halides *via* amine-ligated boryl radicals. LB: Lewis base.

In approaching the development of a general alkynylation reaction of alkyl halides, we identified a strategic advantage in using alkynyl SOMOphiles instead of nucleophilic acetylenes as coupling partners.^9,10^ Among various potential reagents, alkynyl sulfones stood out due to their ease of preparation (one step) and their stability as solid compounds.^11^ However, their intrinsic electrophilic nature transforms the reaction with alkyl halides into a cross-electrophile coupling,^12^ presenting two key challenges for any redox-based system.^13^ Firstly, alkynyl sulfones are more easily reduced than alkyl halides (*E*_red_ ∼ −1.5 V *vs. E*_red_ < −2.0 V *vs.* SCE, respectively),^14^ which complicates radical generation by single-electron transfer (SET). Secondly, the addition of a radical to a sulfone leads to the extrusion of a sulfinyl radical, which must be reduced to a stable sulfinate. This means that two sequential reductions are required along the reaction line, making the overall process redox imbalanced (Scheme 2A).^10,13^

To overcome the first challenge, we considered a halogen atom transfer (XAT)-based approach. Indeed, by using an appropriate abstracting radical (Y·), could achieve selective activation of alkyl halides (Scheme 2A).^15^ However, because the halogen-abstraction step naturally favors the use of nucleophilic radical due to polar effects, it would be crucial to suppress any Giese-type reactions between Y· and the sulfone.^16^

Adopting a XAT-based approach also provides a solution to the second challenge. Indeed, as XAT-mediators can be generated either by oxidation or H-atom transfer (HAT), they provide options for redox-neutral manifolds.^15,17^

Notably, a pioneering application of this concept was developed by Fuchs in 1998,^18^ using alkyl iodides and equimolar di-tin reagents under UV light in benzene. In this system, photochemical homolysis of the Sn–Sn σ-bond generated a tin radical, sustaining a radical chain through XAT on alkyl iodides, followed by radical addition to the alkynyl sulfone and regeneration of the tin-radical. However, the harsh reaction conditions and toxic reagents limited the method's applicability, especially since the more common alkyl bromides could not be activated.

Given our interest in XAT-based methodologies,^15,17*d*,*g*,19^ we hypothesized that the bench stable, non-toxic and inexpensive Lewis acid-base adduct Me_3_N–BH_3_, in combination with diaryl ketone photocatalysis, could address these mechanistic and practical issues.^20^ Specifically, we proposed a catalytic cycle featuring a diarylketone photocatalyst, leveraging HAT and SET events to generate the required amine-ligated boryl radical and enable redox neutral catalysis (Scheme 2B).

Triplet ketones, which are strong HAT mediators, have previously been shown to activate Me_3_N–BH_3_ leading to the formation of I (Scheme 2B).^20*a*,21^ This species could then homolytically activate the alkyl halide by XAT.^21*a*^ The resulting radical III could then undergo α-addition, followed by β-fragmentation, with the sulfone reagent.^10^ This process would yield the desired product 3 and a sulfinate radical, which could close the photocatalytic cycle by SET and deprotonation with the ketyl radical (KR).

### Reaction development

**Scheme 3:**
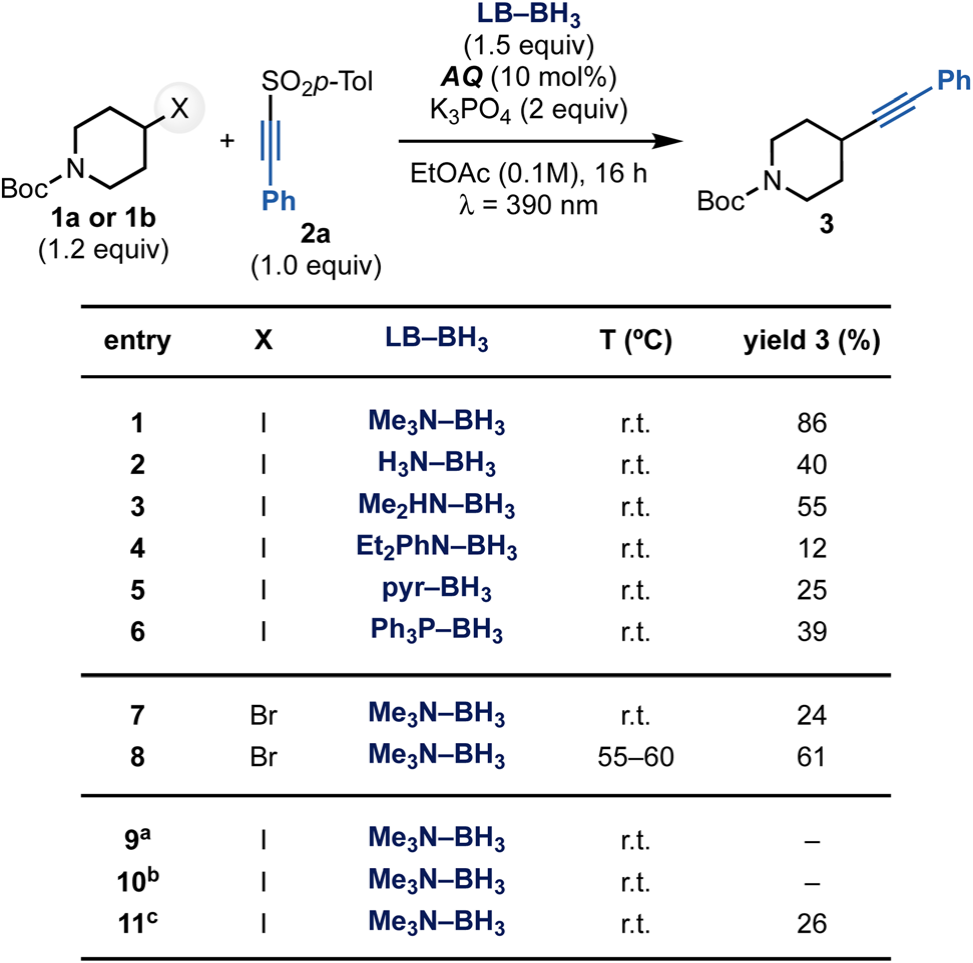
Optimization for the alkynylation of 1a with 2a. ^*a*^Reaction run in the dark. ^*b*^Reaction run without AQ. ^*c*^Reaction run without K_3_PO_4_.

To validate this mechanistic hypothesis, we evaluated the reaction of alkyl iodide 1a and sulfone 2a (Scheme 3). Pleasingly, the use of Me_3_N–BH_3_ as XAT mediator in the presence of K_3_PO_4_ as the base and anthraquinone (AQ) as the photocatalyst under purple LEDs (*λ* = 390 nm) irradiation in EtOAc gave 3 in 86% yield (entry 1, see the ESI† for further optimization studies). Other amine-ligated boranes as well as pyr–BH_3_ and Ph_3_P–BH_3_ could be used in the process but they were all significantly less effective (entries 2–6). This can be mostly rationalized with their lower nucleophilic character that might retard the XAT process. Control experiments confirmed that all components as well as light irradiation are essential for reactivity (entries 9–10), while the base is mostly important to achieve high yields (entry 11). When the same reaction conditions were applied to the alkynylation of bromide 1b, the desired product 3 was obtained in poor conversion (entry 7, see the ESI†). However, conducting the reactions at 55–60 °C restored reactivity, providing 3 in 61% yield (entry 8). We believe the higher temperature might be necessary to facilitate the XAT on the stronger C(sp^3^)–Br bond.^22^

### Reaction scope

**Scheme 4:**
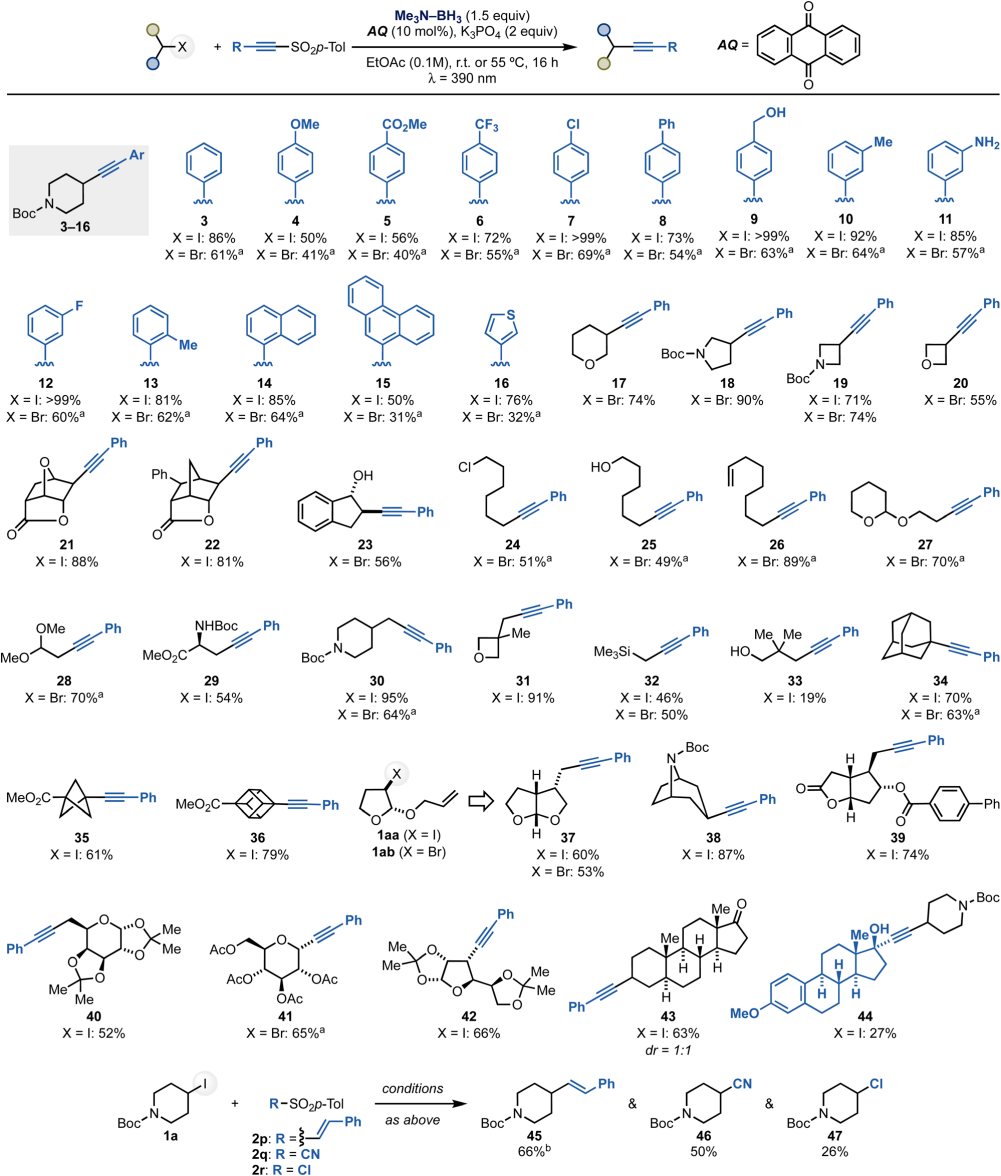
Scope of the process. Unless otherwise noted, reactions were carried out at room temperature using a fan. ^*a*^Reaction run at 55 °C. ^*b*^Reaction run using the SO_2_Ph instead of SO_2_*p*-Tol reagent.

With a method for radical alkynylation of alkyl halides in hand, we proceeded to explore the scope of the process. We initially examined a series of aryl-substituted alkynyl sulfones using 1a and 1b as the coupling partners (Scheme 4). This investigation demonstrated the methodology's compatibility with aromatic units substituted with both electron-donating and electron-withdrawing substituents at the *para* (4–9), *meta* (10–12) and *ortho* (13) positions. Notably, the reactions tolerated ester (5), CF_3_ (6), aryl chloride and fluoride (7 and 12) as well as free alcohol (9) and free aniline (11) functionalities.

Next, we valuated reagents based on (hetero)aromatic systems and successfully extended the reactivity to sulfones containing 1-naphthyl (14), 9-phenanthrenyl (15) and 3-thienyl (16) groups.

The scope of alkyl halides was then explored using 2a as the sulfone coupling partner. Pleasingly, we successfully engaged a range of saturated heterocyclic fragments commonly found in medicinal chemistry libraries.^23^ These included 3-tetrahydropyran (17), 3-*N*-Boc-pryrrolidine (18) as well as 2-*N*-Boc-azetidine (19), 2-oxetane (20) and 2-indenol (23) derivatives. Complex alkyl halides can be conveniently accessed through halo-lactonization processes, which upon alkynylation yielded tricyclic systems 21 and 22 in high diastereoselectivity (dr *exo* : *endo* > 20 : 1).

We then screened primary alkyl halides and demonstrated the chemistry's compatibility with alkyl chloride (24), free alcohol (25), olefin (26) as well as acetal (27–28) and amino acid (29) functionalities. We were then keen on determining if the method could be used for the functionalization of hindered halides. This was demonstrated by engaging a series of derivatives of neo-pentyl nature (31, 33). Moreover, we showcased the reactivity on a series of tertiary halides featuring adamantyl (34), bicyclo[1.1.0]pentyl (35) and cubyl (36) units. The formation of 35 and 36 is particularly noteworthy, given the growing importance of these scaffolds as bioisosteric replacement units in modern medicinal chemistry campaigns.^24^

The radical nature of the transformation was also harnessed as part of radical 5-*exo*-trig cyclization–alkynylation sequences using alkyl halides with tethered alkene functionalities (37). These processes took place in good yield and high diastereoselectivity (see the ESI†).

As a final element of substrate scope, we attempted the functionalization of complex alkyl halides. These species were obtained from the corresponding alcohol in one step by Appel reaction. Pleasingly, we successfully applied this method to the alkynylation of carbohydrate derivatives based on α-d-galactopyranose (40), d-glucose (41) and α-d-glucofuranose (42) moieties. The chemistry was also applied to a derivative of Corey's lactone (39), the alkaloid nortropine (38) and the neurosteroid androsterone (43). Finally, we prepared a sulfonylated ethynylestradiol reagent that was successfully coupled with 1a to give 44. These examples highlight the ability of the method to engage alkyl-substituted acetylenic sulfones as reactive coupling partners.

Notably, this boryl radical-mediated XAT approach can also be used with other sulfone reagents for the transfer of additional functional groups. A preliminary demonstration of this potential is demonstrated by the reaction of 1a with 2p–r under identical reaction conditions to give products of olefination (45), cyanation (46) and chlorination (47).

## Conclusions

The alkynylation of alkyl halides remains a challenging transformation. The results presented here demonstrate that by converting alkyl halides into the corresponding radicals, efficient C(sp^3^)–C(sp) bond formation can be achieved using alkynyl sulfones as the coupling partners. This method leverages amine-ligated boryl radicals for halide activation by halogen-atom transfer (XAT), enabling effective functionalization of primary, secondary and tertiary sites. The broad applicability of this approach has been showcased with the successful alkynylation of alkyl bromides, as well as the functionalization of complex and high-value molecules. We anticipate that this strategy will find utility in the preparation of alkyne-containing materials and further stimulate the development of novel methodologies employing boryl radical-mediated XAT.

## Author contributions

J. C.: conceptualization, investigation, writing – original draft. M. A.: investigation. D. L.: conceptualization, supervision, funding, writing – review & editing.

## Conflicts of interest

There are no conflicts to declare.

## Supplementary Material

SC-015-D4SC06516F-s001

## Data Availability

The data that support the findings of this study are available in the ESI† of this article.
