# Supplementary material for: Boryl radical-mediated halogen-atom transfer (XAT) enables the Sonogashira-like alkynylation of alkyl halides
Source: Chem Sci. 2024 Oct 24;15(45):19113–8. doi: 10.1039/d4sc06516f (PMC11521202; doi:10.1039/d4sc06516f)
Supplement: SC-015-D4SC06516F-s001 [file SC-015-D4SC06516F-s001.pdf]

Supplementary Information (SI) for Chemical Science.
This journal is © The Royal Society of Chemistry 2024

Boryl Radical-Mediated Halogen-Atom Transfer (XAT) Enables the Sonogashira-Like Alkynylation of Alkyl Halides

Javier Corpas,[a] Maialen Alonso, [a] and Daniele Leonori*[a]

[a] *Institute of Organic Chemistry, RWTH Aachen University, Aachen 52056, Germany*

daniele.leonori@rwth-aachen.de

Table of Contents

**1.**	**General experimental details**	**3**
**2.**	**Synthesis of starting materials**	**4**
**3.**	**Reaction optimization**	**7**
**4.**	**General procedures for alknylation of alkyl halides**	**10**
**5.**	**Pictures of reaction set-up**	**12**
**6.**	**Spectra of lamps**	**13**
**7.**	**Starting materials**	**14**
**8.**	**Substrate scope**	**15**
**9.**	**Copies of NMR spectra for new compounds**	**31**
**10.**	**References**	**75**

1. General experimental details

All chemicals were used directly without purification. All air and moisture sensitive reactions were carried out under nitrogen atmosphere using standard Schlenk manifold technique. All solvents were bought from Acros as 99.8% purity. ^{1}H and ^{13}C Nuclear Magnetic Resonance (NMR) spectra were acquired at various field strengths as indicated and were referenced to $CHCl_3$ (7.26 and 77.16 ppm for ^{1}H and ^{13}C respectively). ^{1}H NMR coupling constants are reported in Hertz and refer to apparent multiplicities and not true coupling constants. Data are reported as follows: chemical shift, integration, multiplicity (s = singlet, bs = broad singlet, d = doublet, bd = broad doublet, t = triplet, q = quartet, p = pentet, m = multiplet, dd = doublet of doublets, etc.). ^{19}F NMR spectra were recorded and reported unreferenced. High-resolution mass spectra were obtained using a JEOL JMS-700 spectrometer or a Fissions VG Trio 2000 quadrupole mass spectrometer. Spectra were obtained using electron impact ionization (EI), positive electrospray (ESI) or atmospheric-pressure chemical ionization (APCI). Analytical TLC: aluminum backed plates pre-coated (0.25 mm) with Merck Silica Gel 60 F254. Compounds were visualized by exposure to UV-light or by dipping the plates in permanganate ($KMnO_4$) stain followed by heating. Flash column chromatography was performed using Merck Silica Gel 60 (40–63 μm). All mixed solvent eluents are reported as v/v solutions.

The following alkyl halides were prepared according to reported procedures:[1] **1aa**, **1ab**, **1ac**, **1ad**, **1ae**, **1ag**, and **1ah**. Alkynyl sulfones were prepared following a reported procedure[2] and some of them are known compounds: **2a**,[2] **2b**,[2] **2c**,[3] **2d**,[3] **2e**,[4] **2f**,[4] **2h**,[5] **2j**,[2] **2k**,[3] **2l**,[6] and **2n**.[3]

2. Synthesis of starting materials

General procedure for the synthesis of alkynyl sulfones – GP1.[2]

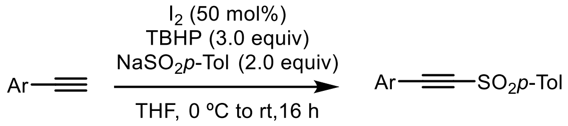

In a 100 mL round-bottom flask provided with a stir bar the terminal alkyne (5.6 mmol, 1.0 equiv), sodium *p*-toluenesulfinate (2 g, 11.2 mmol, 2.0 equiv) and I_2 (710 mg, 2.8 mmol, 0.5 equiv) were dissolved in THF (20 mL) under air. The solution was cooled down to 0 °C using an ice bath, followed by the dropwise addition of TBHP (2.4 mL, 16.8 mmol, 3.0 equiv., 70% aq. sol. in H_2O). The solution was allowed to stir for 16 h ar room temperature and then it was treated with sat. aq. $Na_2S_2O_3$ (50 mL) and stirred for 15 min. The layers were separated and the aqueous phase was extracted with EtOAc (2 x 50 mL). The layers were separated and the organic layer was washed with sat. aq. NaCl (3 x 50 mL). The organic layer was dried ($MgSO_4$), filtered and evaporated. The crude was purified via column chromatography on silica gel eluting with *n*-pentane–EtOAc.

(4-(Tosylethynyl)phenyl)methanol (2g)

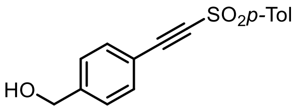

Following **GP1** using (4-ethynylphenyl)methanol (740 mg, 5.6 mmol, 1.0 equiv) gave the titled compound as a solid (74%). R_f 0.32 [*n*-pentane:EtOAc (3:1)]. ^{1}H NMR (600 MHz, $CDCl_3$) δ 7.95 (2H, d, *J* = 8.4 Hz), 7.55–7.48 (2H, m), 7.39 (2H, d, *J* = 8.0 Hz), 7.37 (2H, d, *J* = 8.0 Hz), 4.73 (2H, s), 2.47 (3H, s). ^{13}C NMR (151 MHz, $CDCl_3$) δ 145.5, 144.8, 139.0, 133.1, 130.1, 127.7, 126.9, 117.1, 93.1, 85.7, 64.6, 21.9. HRMS (EI) calculated $C_{16}H_{14}O_3SK$ [M+K$^+$] 325.02952 found 325.02917.

3-(Tosylethynyl)aniline (2l)

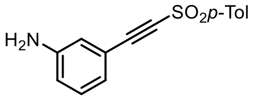

Following **GP1** using 3-ethynylaniline (656 mg, 5.6 mmol, 1.0 equiv) gave the titled compound as an oil (51%). R_f 0.35 [*n*-pentane: EtOAc (4:1)]. ^{1}H NMR (400 MHz, $CDCl_3$) δ 7.93 (2H, d, *J* = 8.0 Hz), 7.37 (2H, d, *J* = 8.0 Hz), 7.10 (1H, t, *J* = 7.8 Hz), 6.86 (1H, d, *J* = 7.6 Hz), 6.79–6.61 (2H, m), 3.80 (2H, s), 2.45 (3H, s). ^{13}C NMR (101 MHz, $CDCl_3$, one quaternary carbon

does not appear due to long relaxation time) δ 146.8, 145.4, 138.9, 130.0, 129.6, 127.4, 122.6, 118.3, 118.1, 93.9, 84.8, 21.7. HRMS (EI) calculated for $C_{15}H_{13}NO_2S$ [M^+] 271.0660 found 271.0665.

9-(Tosylethynyl)phenanthrene (2m)

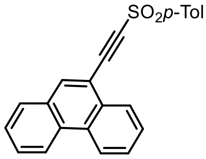

Following **GP1** using 9-ethynylphenanthrene (1.13 g, 5.6 mmol, 1.0 equiv) gave the titled compound as a solid (62%). R_f 0.43 [*n*-pentane: EtOAc (6:1)]. ^{1}H NMR (600 MHz, $CDCl_3$) δ 8.67 (1H, d, *J* = 8.2 Hz), 8.64 (1H, d, *J* = 8.3Hz), 8.17 (1H, dd, *J* = 7.9, 1.4 Hz), 8.12 (1H, s), 8.08 – 8.03 (2H, m), 7.85 (1H, dd, *J* = 8.1, 1.4 Hz), 7.76–7.70 (2H, m), 7.67 (1H, ddd, *J* = 8.2, 7.0, 1.3 Hz), 7.63 (1H, ddd, *J* = 8.0, 6.9, 1.1 Hz), 7.43 (2H, d, *J* = 8.1 Hz), 2.49 (3H, s). ^{13}C NMR (151 MHz, $CDCl_3$) δ 145.6, 139.3, 135.9, 131.6, 130.4, 130.2, 130.0, 129.4, 127.9, 127.9, 127.7, 127.5, 126.4, 123.1, 122.9, 114.6, 92.3, 89.8, 21.9. HRMS (EI) calculated $C_{23}H_{16}O_2S$ [M^+] 356.0871 found 356.0871.

Synthesis of the synthesis of (*8R,9S,13S,14S,17S*)-3-methoxy-13-methyl-17-(tosylethynyl)-7,8,9,11,12,13,14,15,16,17-decahydro-6*H*-cyclopenta[*a*]phenanthren-17-ol (2o).

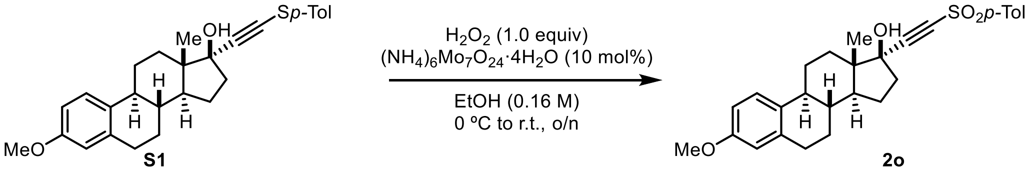

Mestranol sulfide **S1** was prepared according to a known procedure.[7] In a 25 mL round-bottom flask provided with a stir bar, the sulfide **S1** (606 mg, 1.4 mmol, 1.0 equiv) was dissolved in EtOH (0.16 M). To this solution, ammonium heptamolybdate (173 mg, 0.14 mmol, 10 mol%) and H_2O_2 (43 µL, 1.4 mmol, 1.0 equiv, 30% in H_2O) were added at 0 °C and the reaction was stirred at room temperature. After 16 h the solvent was evaporated under vacuo and the crude was dissolved in H_2O (20 ml) and EtOAc (15 mL). The organic layer was separated and the aqueous layer was extracted with EtOAc (15 ml x 2). The organic layer was dried ($MgSO_4$), filtered and evaporated. The crude product was purified by column chromatography to obtain the desired sulfone **2o** (95%) as a solid. R_f 0.45 [*n*-pentane: EtOAc (4:1)]. ^{1}H NMR (400 MHz, $CDCl_3$) δ 7.90 (2H, d, *J* = 8.0 Hz), 7.37 (2H, d, *J* = 8.0 Hz), 7.16 (1H, d, *J* = 8.6 Hz), 6.72 (1H,

dd, *J* = 8.6, 2.8 Hz), 6.63 (1H, d, *J* = 2.8 Hz), 3.78 (3H, s), 2.93–2.71 (2H, m), 2.42 (3H, s), 2.37–2.20 (2H, m), 2.10–1.89 (3H, m), 1.92–1.72 (1H, m), 1.68 (1H, d, *J* = 12.3 Hz), 1.53–1.22 (6H, m), 0.85 (3H, s). ^{13}C NMR (101 MHz, $CDCl_3$) δ 157.7, 138.0, 132.2, 130.1, 127.5, 126.4, 114.0, 111.7, 98.3, 80.2, 77.4, 55.4, 50.2, 48.7, 43.4, 39.4, 38.5, 33.0, 29.9, 27.3, 26.3, 23.1, 21.9, 12.8. HRMS (APCI) calculated for $C_{28}H_{33}O_4S$ [M+H^+] 465.2100 found 465.2099.

3. Reaction optimization

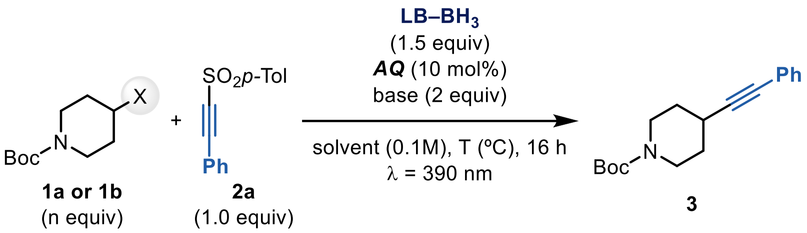

General procedure for optimization reactions: An oven-dried 8 mL microwave provided with a stir bar was charged with the corresponding alkyl halide (1.0–2.0 equiv), **2a** (26 mg, 0.1 mmol, 1.0 equiv), the base (0.2 mmol, 2.0 equiv), the ligated amine-borane (0.15 mmol, 1.5 equiv) and anthraquinone (2 mg, 0.01 mmol, 10 mol%). The vial was capped with a Supelco aluminium crimp seal with septum (PTFE/butyl), evacuated and refilled with N_2 (x 3). Then, degassed solvent (1.0 mL), was added. The vial was sealed with parafilm and the reaction was placed approximately 4 cm from purple LEDs (390 nm Kessil lamp). The LEDs were switched on and the mixture was stirred under irradiation at the specified temperature for 16 h. The tube was opened and the mixture was diluted with brine (10 mL) and EtOAc (5 mL). The layers were separated and the aqueous layer was extracted with EtOAc (2 × 5 mL). The combined organic layers were dried ($MgSO_4$), filtered and evaporated. A solution of trichloroethylene (0.1 mL, 1.0 M in $CDCl_3$) was added, the crude was solubilised in $CDCl_3$ (0.6 mL) and the mixture analysed by ^1H NMR spectroscopy to obtain the reaction NMR yield.

Table S1. Optimization of amine-borane.

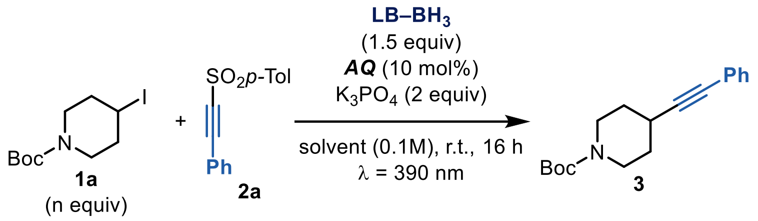

Entry	LB–BH3	Solvent	1a (n equiv)	Yield 3 (%)[a]
**1**	H_3N–BH_3	EtOAc	1.2	40
**2**	Me_2HN–BH_3	EtOAc	1.2	55
**3**[a]	Me_3N–BH_3	EtOAc	1.2	86
**4**	Et_2PhN–BH_3	EtOAc	1.2	12
**5**	pyr–BH_3	EtOAc	1.2	25
**6**	Ph_3P–BH_3	EtOAc	1.2	39
**7**	Me_3N–BH_3	CH_2Cl_2	1.2	–
**8**	Me_3N–BH_3	DCE	1.2	–
**9**	Me_3N–BH_3	THF	1.2	57
**10**	Me_3N–BH_3	1,4-dioxane	1.2	62
**11**	Me_3N–BH_3	Toluene	1.2	50
**12**	Me_3N–BH_3	$PhCF_3$	1.2	41
**13**	Me_3N–BH_3	CH_3CN	1.2	69
**14**	Me_3N–BH_3	MeOH	1.2	35
**15**	Me_3N–BH_3	EtOAc	1.0	70
**16**	Me_3N–BH_3	EtOAc	2.0	88
**17**	–	EtOAc	1.2	–
**18**[b]	Me_3N–BH_3	EtOAc	1.2	–
**19**[c]	Me_3N–BH_3	EtOAc	1.2	–
**20**	Me_3N–BH_3	EtOAc	1.2	26

[a]Determined in the reaction crude by ^{1}H NMR spectroscopy employing trichloroethylene as an internal standard.
[b]Reaction run in the dark. [c]No AQ was added. [d]Reaction run in the absence of base.

Table S2. Optimization of base.

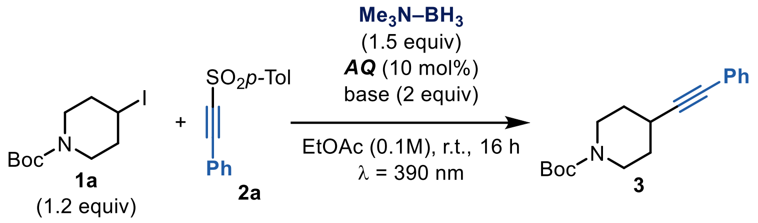

Entry	base	Yield 3 (%)
**1**	K_3PO_4	86
**2**	pyridine	46
**3**	2,6-lutidine	57
**4**	Cs_2CO_3	69
**5**	K_2CO_3	75
**6**	Na_2CO_3	69
**7**	K_2HPO_4	77
**8**	DBU	–
**9**	TMG	–

Table S3. Optimization for alkyl bromides.

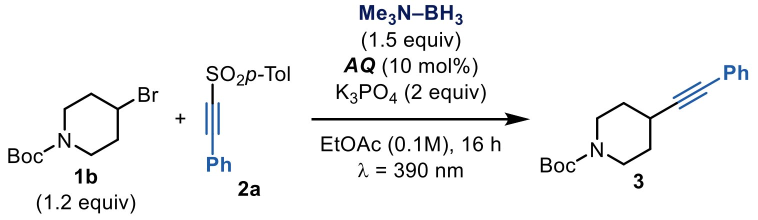

Entry	T (ºC)	Yield 3 (%)
**1**	25	24
**2**	35	38
**3**	55–60	61
**4**	80	41

4. General procedures for alknylation of alkyl halides

General procedure for the alkynylation at room temperature – GP2

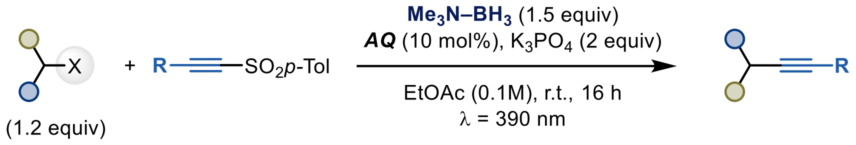

An oven-dried 8 mL microwave provided with a stir bar was charged with the alkyl halide if solid (0.12 mmol, 1.2 equiv), the corresponding sulfone (0.1 mmol, 1.0 equiv), K_3PO_4 (43 mg, 0.2 mmol, 2.0 equiv), $Me_3N–BH_3$ (11 mg, 0.15 mmol, 1.5 equiv) and anthraquinone (2 mg, 0.01 mmol, 10 mol%). The vial was capped with a Supelco aluminium crimp seal with septum (PTFE/butyl), evacuated and refilled with N_2 (x 3). Then, degassed EtOAc (1.0 mL), and the alkyl halide if liquid (0.12 mmol, 1.2 equiv) were added. The vial was sealed with parafilm and the reaction was placed approximately 4 cm from purple LEDs (390 nm Kessil lamp). The LEDs were switched on and the mixture was stirred under irradiation at room temperature for 16 h with a fan. The tube was opened and the mixture was diluted with brine (10 mL) and EtOAc (5 mL). The layers were separated and the aqueous layer was extracted with EtOAc (2 × 5 mL). The combined organic layers were dried ($MgSO_4$), filtered and evaporated. The crude was purified by flash column chromatography on silica gel to give the corresponding product.

General procedure for the alkynylation at 55 ºC – GP3

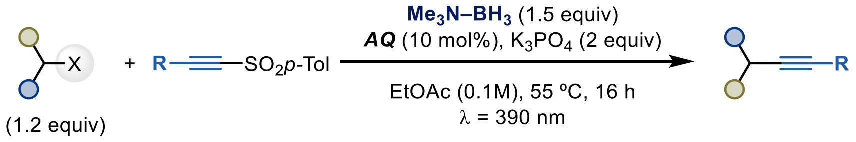

An oven-dried 8 mL microwave provided with a stir bar was charged with the alkyl halide if solid (0.12 mmol, 1.2 equiv), the corresponding sulfone (0.1 mmol, 1.0 equiv), K_3PO_4 (43 mg, 0.2 mmol, 2.0 equiv), $Me_3N–BH_3$ (11 mg, 0.15 mmol, 1.5 equiv) and anthraquinone (2 mg, 0.01 mmol, 10 mol%). The vial was capped with a Supelco aluminium crimp seal with septum (PTFE/butyl), evacuated and refilled with N_2 (x 3). Then, degassed EtOAc (1.0 mL), and the alkyl halide if liquid (0.12 mmol, 1.2 equiv) were added. The vial was sealed with parafilm and the reaction was placed approximately 4 cm from purple LEDs (390 nm Kessil lamp). The LEDs were switched on and the mixture was stirred under irradiation, reaching a constant temperature of 55 ºC. The tube was allowed to cool down to room temperature and then was

opened, diluting the mixture with brine (10 mL) and EtOAc (5 mL). The layers were separated and the aqueous layer was extracted with EtOAc (2 × 5 mL). The combined organic layers were dried ($MgSO_4$), filtered and evaporated. The crude was purified by flash column chromatography on silica gel to give the corresponding product.

5. Pictures of reaction set-up

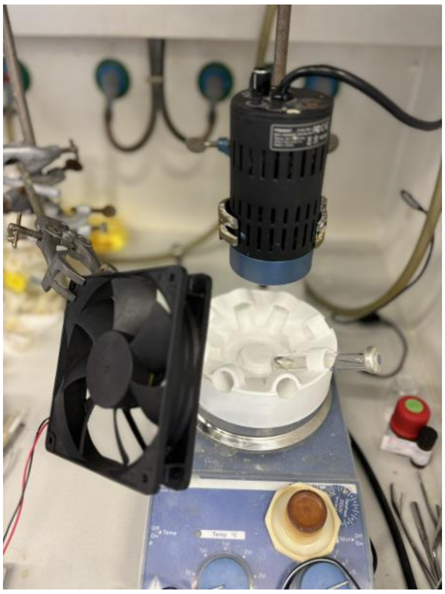

Figure S1. Set up for photochemical reactions under 390 nm with a Kessil lamp.

6. Spectra of lamps

The spectra of the Kessil lamps were taken from the Kessil website: https://www.kessil.com/products/science_PR160L.php

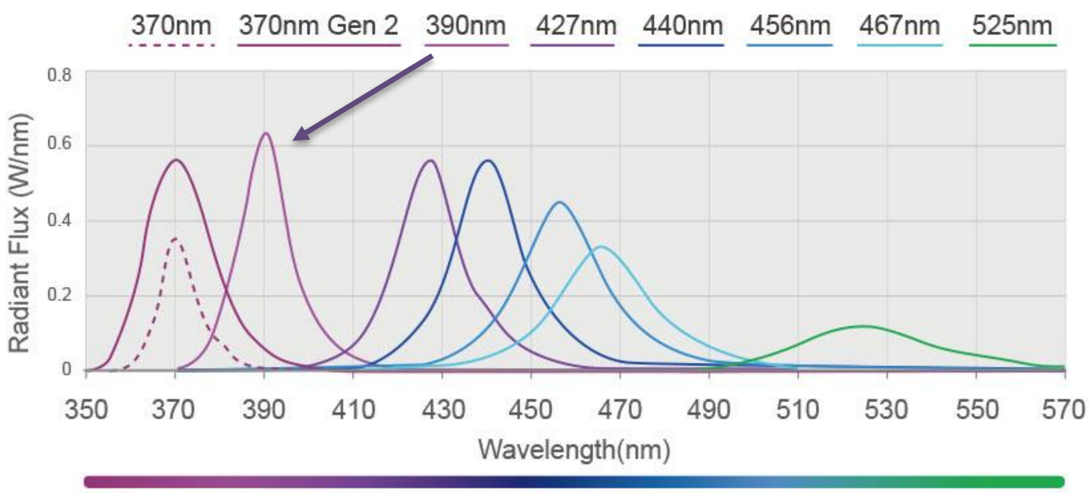

Figure S2.

7. Starting materials

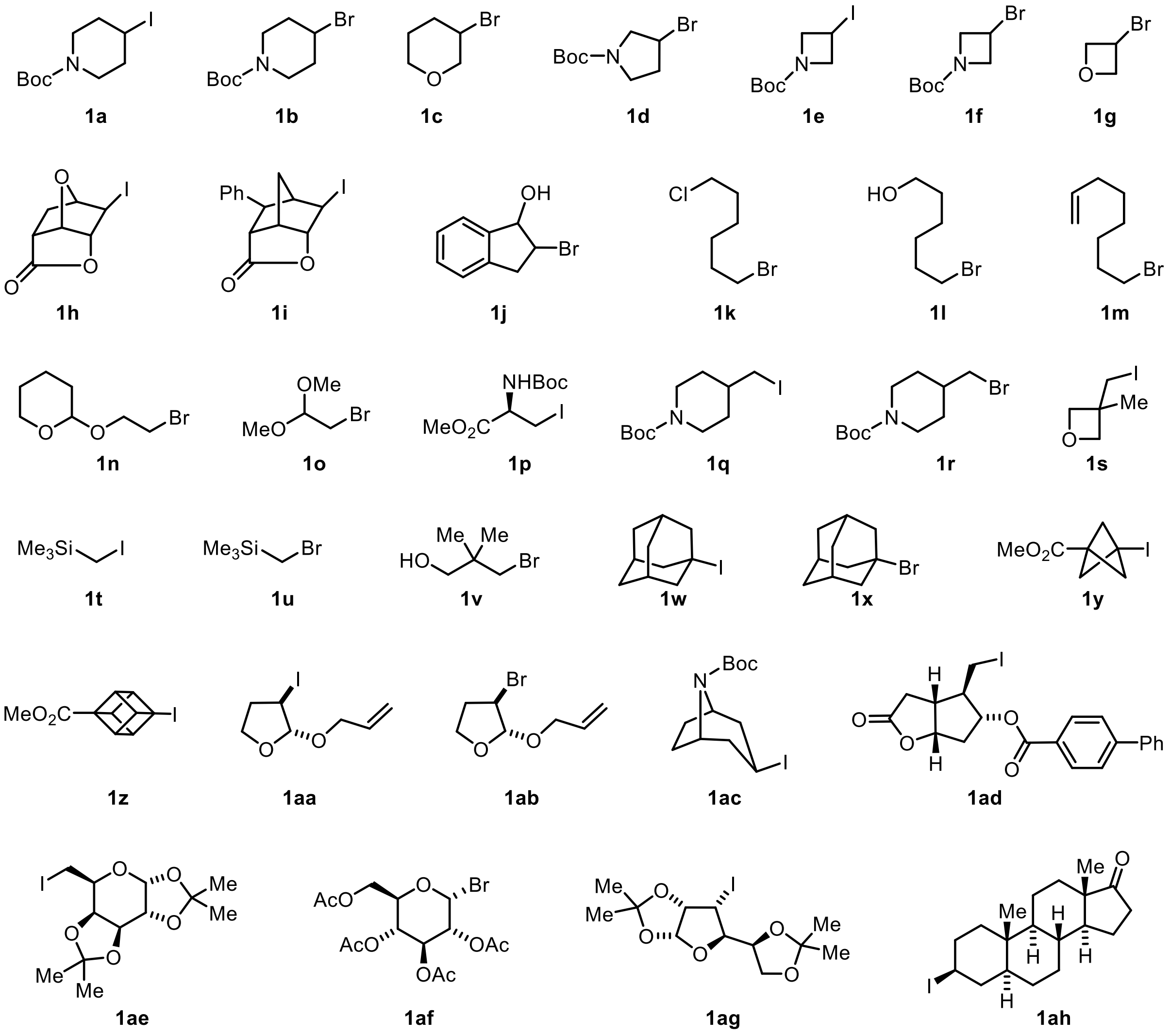

Figure S3. Alkyl halides employed for the substrate scope.

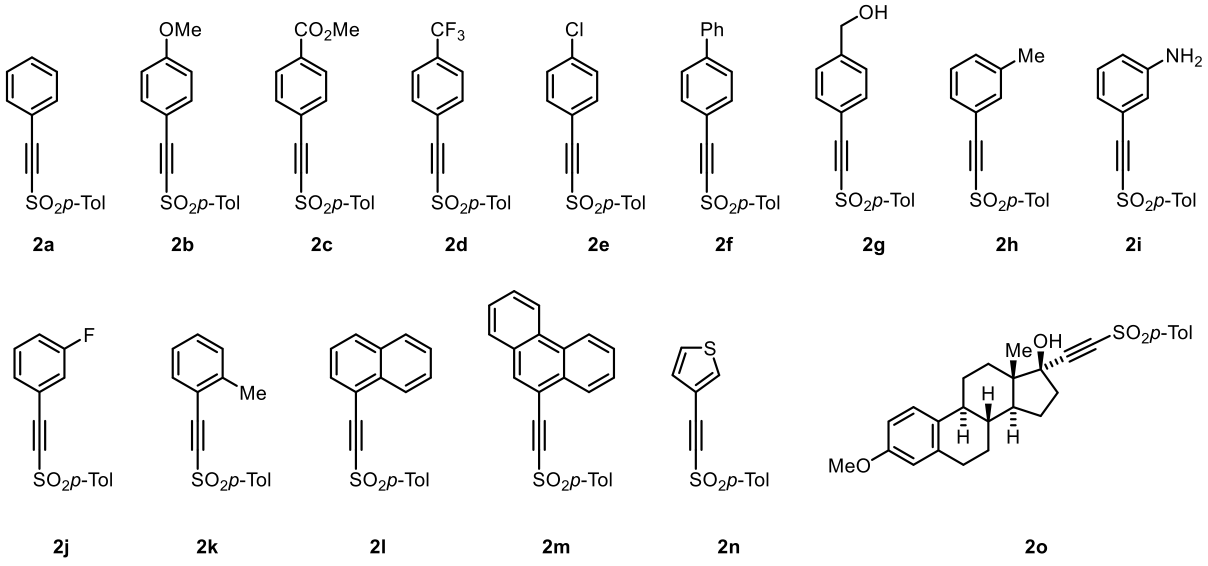

Figure S4. Alkynyl sulfones employed for the substrate scope.

8. Substrate scope

***tert*-Butyl 4-(phenylethynyl)piperidine-1-carboxylate (3)**

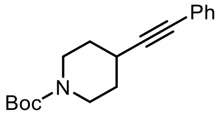

Following **GP2** using **1a** (37 mg, 0.12 mmol, 1.2 equiv) and **2a** (26 mg, 0.1 mmol, 1 equiv) gave **3** (86%) as a solid. Following **GP3** using **1b** (26 mg, 0.12 mmol, 1.2 equiv) gave **3** (61%). R_f 0.50 [*n*-pentane: EtOAc (15:1)]. ^{1}H NMR (600 MHz, $CDCl_3$) δ 7.42–7.37 (2H, m), 7.29–7.26 (3H, m), 3.78–3.70 (2H, m), 3.28–3.21 (2H, m), 2.82–2.76 (1H, m), 1.89–1.82 (2H, m), 1.71–1.63 (2H, m), 1.47 (9H, s); ^{13}C NMR (151 MHz, $CDCl_3$) δ 154.8, 131.6, 128.2, 127.8, 123.6, 91.8, 82.0, 79.4, 43.2 (bs) 31.4, 28.5, 27.6. Data in accordance with the literature.[8]

***tert*-Butyl 4-((4-methoxyphenyl)ethynyl)piperidine-1-carboxylate (4)**

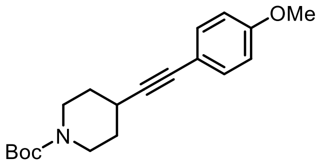

Following **GP2** using **1a** (37 mg, 0.12 mmol, 1.2 equiv) and **2b** (29 mg, 0.1 mmol, 1 equiv) gave **4** (50%) as a solid. Following **GP3** using **1b** (26 mg, 0.12 mmol, 1.2 equiv) gave **4** (41%). R_f 0.50 [*n*-pentane: EtOAc (15:1)]. ^{1}H NMR (600 MHz, $CDCl_3$) δ 7.32 (2H, d, *J*=8.8 Hz), 6.81 (2H, d, *J*=8.8 Hz), 3.79 (3H, s), 3.75–3.72 (2H, m), 3.26–3.21 (2H, m), 2.80–2.75 (1H, m), 1.86–1.82 (2H, m), 1.69–1.62 (2H, m), 1.46 (9H, s); ^{13}C NMR (151 MHz, $CDCl_3$) δ 159.3, 154.9, 133.0, 115.8, 113.9, 90.3, 81.8, 79.5, 55.3, 42.6, 42.0, 31.6, 28.5, 27.7. Data in accordance with the literature.[9]

***tert*- Butyl 4-((4-(methoxycarbonyl)phenyl)ethynyl)piperidine-1-carboxylate (5)**

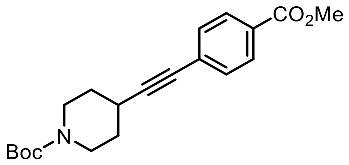

Following **GP2** using **1a** (37 mg, 0.12 mmol, 1.2 equiv) and **2c** (31 mg, 0.1 mmol, 1 equiv) gave **5** (56%) as a solid. Following **GP3** using **1b** (26 mg, 0.12 mmol, 1.2 equiv) gave **5** (40%). R_f 0.43 [*n*-pentane: EtOAc (15:1)]. ^{1}H NMR (600 MHz, $CDCl_3$) δ 7.96 (2H, d, *J*=8.4 Hz), 7.44 (2H, d, *J*=8.4 Hz), 3.91 (3H, s), 3.81–3.67 (2H, m), 3.24 (2H, ddd, *J*=13.2, 8.5, 3.3 Hz), 2.81 (1H, tt, *J*=8.0, 3.9 Hz), 1.93–1.81 (2H, m), 1.73–1.62 (2H, m), 1.46 (9H, s); ^{13}C NMR (151 MHz, $CDCl_3$) δ 166.8, 154.9, 131.7, 129.6, 128.5, 127.3, 95.3, 81.6, 79.7, 52.3, 35.4, 31.4, 28.6, 27.9; HRMS (ESI) calculated $C_{20}H_{25}O_4NNa$ [M+Na^+] 366.1676 found 366.1672.

***tert*-Butyl 4-((4-(trifluoromethyl)phenyl)ethynyl)piperidine-1-carboxylate (6)**

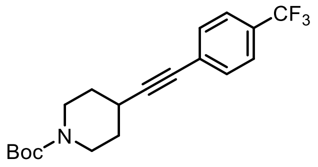

Following **GP2** using **1a** (37 mg, 0.12 mmol, 1.2 equiv) and **2d** (32 mg, 0.1 mmol, 1 equiv) gave **4** (72%) as an oil. Following **GP3** using **1b** (26 mg, 0.12 mmol, 1.2 equiv) gave **6** (55%). R_f 0.48 [*n*-pentane: EtOAc (15:1)]. ^{1}H NMR (600 MHz, $CDCl_3$) δ 7.54 (2H, d, *J* = 8.1 Hz), 7.49 (2H, d, *J* = 8.1 Hz), 3.77–3.68 (2H, m), 3.24 (2H, ddd, *J* = 13.4, 8.5, 3.4 Hz), 2.81 (1H, tt, *J* = 8.1, 4.0 Hz), 1.90–1.81 (2H, m), 1.74–1.63 (2H, m), 1.47 (9H, s). ^{13}C NMR (151 MHz, $CDCl_3$, quaternary carbons were not observed due to the relaxation time) δ 154.9, 133.7, 132.0, 125.3 (q, *J* = 4.0 Hz), 94.7, 80.9, 79.7, 31.4, 28.6, 27.8; ^{19}F NMR (565 MHz, $CDCl_3$) δ -62.78; HRMS (APCI) calculated $C_{19}H_{23}F_3NO_2$ [M+H$^+$] 353.1603 found 353.1603.

***tert*-Butyl 4-((4-chlorophenyl)ethynyl)piperidine-1-carboxylate (7)**

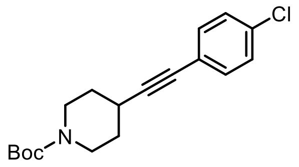

Following **GP2** using **1a** (37 mg, 0.12 mmol, 1.2 equiv) and **2e** (29 mg, 0.1 mmol, 1 equiv) gave **7** (>99%) as an oil. Following **GP3** using **1b** (26 mg, 0.12 mmol, 1.2 equiv) gave **7** (69%). R_f 0.50 [*n*-pentane: EtOAc (15:1)]. ^{1}H NMR (600 MHz, $CDCl_3$) δ 7.31 (2H, d, *J*=8.5 Hz), 7.25 (2H, d, *J*=8.5 Hz), 3.77–3.70 (2H, m), 3.23 (2H, ddd, *J*=13.2, 8.5, 3.3 Hz), 2.78 (1H, tt, *J*=8.0, 3.9 Hz), 1.88–1.81 (2H, m), 1.71–1.61 (2H, m), 1.46 (9H, s); ^{13}C NMR (151 MHz, $CDCl_3$) δ 154.9, 133.9, 133.0, 128.7, 122.2, 93.0, 81.0, 79.7, 42.9, 31.5, 28.6, 27.8; HRMS (ESI) calculated $C_{18}H_{22}O_2NClNa$ [M+Na$^+$] 342.1231 found 342.1227.

***tert*-Butyl 4-([1,1'-biphenyl]-4-ylethynyl)piperidine-1-carboxylate (8)**

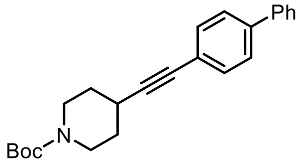

Following **GP2** using **1a** (37 mg, 0.12 mmol, 1.2 equiv) and **2f** (33 mg, 0.1 mmol, 1 equiv) gave **8** (73%) as an oil. Following **GP3** using **1b** (26 mg, 0.12 mmol, 1.2 equiv) gave **8** (54%). R_f 0.48 [*n*-pentane: EtOAc (20:1)]. ^{1}H NMR (600 MHz, $CDCl_3$) δ 7.60–7.57 (2H, m), 7.53 (2H, d, *J*=8.4 Hz), 7.47 (2H, d, *J*=8.4 Hz), 7.44 (2H, t, *J*=7.7 Hz), 7.37–7.33 (1H, m), 3.79–3.69 (2H, m), 3.26 (2H, ddd, *J*=12.0, 8.4, 3.3 Hz), 2.82 (1H, tt, *J*=8.0, 3.9 Hz), 1.91–1.78 (2H,

m), 1.73–1.63 (2H, m), 1.47 (9H, s); ^{13}C NMR (151 MHz, $CDCl_3$) δ 155.0, 140.7, 140.6, 132.1, 129.0, 127.7, 127.1, 127.0, 122.6, 92.7, 82.0, 79.6, 42.6, 31.6, 28.6, 27.8; HRMS (ESI) calculated $C_{24}H_{27}O_2Na$ [M+Na$^+$] 384.1934 found 384.1926.

***tert*-Butyl 4-((4-(hydroxymethyl)phenyl)ethynyl)piperidine-1-carboxylate (9)**

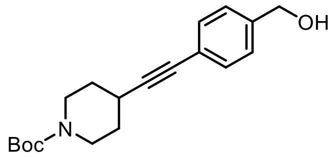

Following **GP2** using **1a** (37 mg, 0.12 mmol, 1.2 equiv) and **2g** (29 mg, 0.1 mmol, 1 equiv) gave **9** (>99%) as a solid. Following **GP3** using **1b** (26 mg, 0.12 mmol, 1.2 equiv) gave **9** (54%). R_f 0.31 [*n*-pentane: EtOAc (3:1). ^{1}H NMR (600 MHz, $CDCl_3$) δ 7.39 (2H, d, *J*=8.2 Hz), 7.29 (2H, d, *J*=8.1 Hz), 4.68 (2H, s), 3.77–3.69 (2H, m), 3.24 (2H, ddd, *J*=13.2, 8.4, 3.4 Hz), 2.79 (1H, tt, *J*=8.0, 3.9 Hz), 1.90–1.80 (2H, m), 1.70–1.63 (2H, m), 1.46 (9H, s); ^{13}C NMR (151 MHz, $CDCl_3$) δ 155.0, 140.6, 131.9, 126.9, 123.0, 92.0, 81.9, 79.6, 65.1, 42.0, 31.6, 28.6, 27.8; HRMS (ESI) calculated $C_{19}H_{25}O_3NNa$ [M+Na $^+$] 338.1727 found 338.1723.

***tert*-Butyl 4-(*m*-tolylethynyl)piperidine-1-carboxylate (10)**

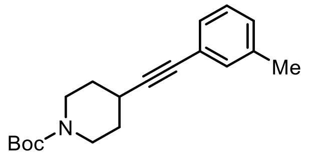

Following **GP2** using **1a** (37 mg, 0.12 mmol, 1.2 equiv) and **2h** (27 mg, 0.1 mmol, 1 equiv) gave **10** (92%) as an oil. Following **GP3** using **1b** (26 mg, 0.12 mmol, 1.2 equiv) gave **10** (63%). R_f 0.51 [*n*-pentane: EtOAc (15:1)]. ^{1}H NMR (600 MHz, $CDCl_3$) δ 7.23 (1H, bs), 7.20 (1H, bd, *J*=7.7 Hz), 7.17 (1H, t, *J*=7.5 Hz), 7.09 (1H, bd, *J*=7.4 Hz), 3.77–3.68 (2H, m), 3.25 (2H, ddd, *J*=13.2, 8.3, 3.4 Hz), 2.79 (1H, tt, *J*=7.9, 3.9 Hz), 2.31 (3H, s), 1.87–1.81 (2H, m), 1.71–1.62 (2H, m), 1.47 (9H, s); ^{13}C NMR (151 MHz, $CDCl_3$) δ 155.0, 138.0, 132.4, 128.8, 128.8, 128.3, 123.5, 91.6, 82.3, 79.6, 63.2, 31.6, 28.6, 27.7, 21.3; HRMS (ESI) calculated $C_{19}H_{25}O_2NNa$ [M+Na $^+$] 322.1778 found 322.1778.

***tert*-Butyl 4-((3-aminophenyl)ethynyl)piperidine-1-carboxylate (11)**

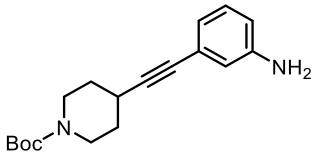

Following **GP2** using **1a** (37 mg, 0.12 mmol, 1.2 equiv) and **2i** (27 mg, 0.1 mmol, 1 equiv) gave **11** (85%) as an oil. Following **GP3** using **1b** (26 mg, 0.12 mmol, 1.2 equiv) gave **11** (57%). R_f 0.46 [*n*-pentane: EtOAc (2:1)]. ^{1}H NMR (600 MHz, $CDCl_3$) δ 7.06 (1H, t, *J*=7.8 Hz),

6.80 (1H, d, *J*=7.6 Hz), 6.72 (1H, bs), 6.61 (1H, dd, *J*=8.0, 2.1 Hz), 3.76–3.67 (2H, m), 3.64 (2H, bs), 3.25 (2H, ddd, *J*=13.1, 8.3, 3.3 Hz), 2.82–2.74 (1H, m), 1.87–1.80 (2H, m), 1.70–1.62 (2H, m), 1.46 (9H, s); ^{13}C NMR (151 MHz, $CDCl_3$) δ 154.8, 146.2, 129.2, 124.2, 122.0, 117.9, 114.8, 91.2, 82.1, 79.4, 63.6, 31.4, 28.4, 27.5; HRMS (ESI) calculated $C_{18}H_{25}O_2N_2$ [M+H$^+$] 301.1911 found 301.1905.

***tert*-Butyl 4-((3-fluorophenyl)ethynyl)piperidine-1-carboxylate (12)**

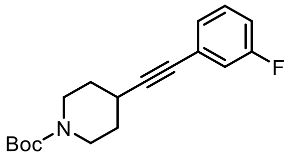

Following **GP2** using **1a** (37 mg, 0.12 mmol, 1.2 equiv) and **2j** (27 mg, 0.1 mmol, 1 equiv) gave **12** (>99%) as an oil. Following **GP3** using **1b** (26 mg, 0.12 mmol, 1.2 equiv) gave **12** (60%). R_f 0.50 [*n*-pentane: EtOAc (15:1)]. ^{1}H NMR (600 MHz, $CDCl_3$) δ 7.25–7.22 (1H, m), 7.16 (1H, dt, *J*=7.7, 1.2 Hz), 7.08 (1H, ddd, *J*=9.6, 2.4, 1.4 Hz), 7.00–6.96 (1H, m), 3.77–3.68 (2H, m), 3.27–3.19 (2H, m), 2.79 (1H, tt, *J*=8.0, 3.9 Hz), 1.88–1.81 (2H, m), 1.69–1.63 (2H, m), 1.46 (9H, s); ^{13}C NMR (151 MHz, $CDCl_3$) δ 162.5 (d, *J*=246.0 Hz), 154.9, 129.9 (d, *J*=8.8 Hz), 127.6 (d, *J*=2.9 Hz), 125.6 (d, *J*=9.7 Hz), 118.6 (d, *J*=22.6 Hz), 115.3 (d, *J*=21.2 Hz), 93.0, 81.0, 79.7, 62.6, 31.5, 28.6, 27.7; ^{19}F NMR (565 MHz, $CDCl_3$) δ -113.30; HRMS (ESI) calculated $C_{18}H_{22}O_2NFNa$ [M+Na$^+$] 326.1527 found 326.1512.

***tert*-Butyl 4-(*o*-tolylethynyl)piperidine-1-carboxylate (13)**

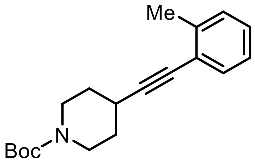

Following **GP2** using **1a** (37 mg, 0.12 mmol, 1.2 equiv) and **2k** (27 mg, 0.1 mmol, 1 equiv) gave **13** (81%) as an oil. Following **GP3** using **1b** (26 mg, 0.12 mmol, 1.2 equiv) gave **13** (62%). R_f 0.48 [*n*-pentane: EtOAc (15:1)]. ^{1}H NMR (600 MHz, $CDCl_3$) δ 7.36 (1H, d, *J*=7.5 Hz), 7.22–7.14 (2H, m), 7.11–7.09 (1H, m), 3.77–3.64 (2H, m), 3.31 (2H, ddd, *J*=13.2, 8.0, 3.4 Hz), 2.87 (1H, tt, *J*=7.8, 4.0 Hz), 2.41 (3H, s), 1.91–1.84 (2H, m), 1.76–1.65 (2H, m), 1.47 (9H, s); ^{13}C NMR (151 MHz, $CDCl_3$) δ 155.0, 140.1, 131.9, 129.5, 127.9, 125.6, 123.4, 96.0, 81.0, 79.6, 62.7, 31.7, 28.6, 27.8, 21.0; HRMS (ESI) calculated $C_{19}H_{25}O_2NNa$ [M+Na$^+$] 322.1778 found 322.1778.

***tert*-Butyl 4-(naphthalen-1-ylethynyl)piperidine-1-carboxylate (14)**

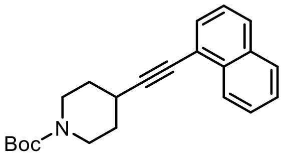

Following **GP2** using **1a** (37 mg, 0.12 mmol, 1.2 equiv) and **2l** (31 mg, 0.1 mmol, 1 equiv) gave **14** (85%) as an oil. Following **GP3** using **1b** (26 mg, 0.12 mmol, 1.2 equiv) gave **14** (64%). R_f 0.43 [*n*-pentane: EtOAc (15:1)]. ^{1}H NMR (600 MHz, $CDCl_3$) δ 8.30 (1H, d, *J*=8.3 Hz), 7.84 (1H, d, *J*=8.1 Hz), 7.79 (1H, d, *J*=8.3 Hz), 7.63 (1H, d, *J*=7.1 Hz), 7.58–7.54 (1H, m), 7.52–7.49 (1H, m), 7.43–7.38 (1H, m), 3.85–3.74 (2H, m), 3.32 (2H, ddd, *J*=13.1, 8.3, 3.4 Hz), 2.97 (1H, tt, *J*=8.0, 4.0 Hz), 2.00–1.92 (2H, m), 1.85–1.73 (2H, m), 1.48 (9H, s); ^{13}C NMR (151 MHz, $CDCl_3$) δ 155.0, 133.5, 133.3, 130.3, 128.4, 128.4, 126.8, 126.4, 126.2, 125.3, 121.3, 97.0, 80.1, 79.7, 42.2, 31.8, 28.6, 28.1; HRMS (ESI) calculated $C_{22}H_{25}O_2NNa$ [M+Na$^+$] 358.1778 found 358.1763.

***tert*-Butyl 4-(phenanthren-9-ylethynyl)piperidine-1-carboxylate (15)**

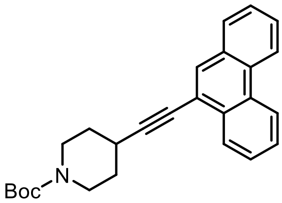

Following **GP2** using **1a** (37 mg, 0.12 mmol, 1.2 equiv) and **2m** (36 mg, 0.1 mmol, 1 equiv) gave **15** (50%) as a solid. Following **GP3** using **1b** (26 mg, 0.12 mmol, 1.2 equiv) gave **15** (31%). R_f 0.42 [*n*-pentane: EtOAc (20:1)]. ^{1}H NMR (600 MHz, $CDCl_3$) δ 8.70–8.67 (1H, m), 8.65 (1H, d, *J*=8.2 Hz), 8.41 (1H, dd, *J*=7.7, 1.5 Hz), 7.95 (1H, s), 7.83 (1H, d, *J*=7.8 Hz), 7.71–7.63 (3H, m), 7.61–7.56 (1H, m), 3.88–3.79 (2H, m), 3.34 (2H, ddd, *J*=13.1, 8.4, 3.3 Hz), 3.00 (1H, tt, *J*=7.9, 3.9 Hz), 2.04–1.92 (2H, m), 1.87–1.80 (2H, m), 1.49 (9H, s); ^{13}C NMR (151 MHz, $CDCl_3$) δ 155.0, 131.6, 131.5, 131.4, 130.3, 130.2, 128.5, 127.4, 127.1, 127.1, 127.0, 127.0, 122.9, 122.7, 120.0, 96.7, 80.3, 79.7, 42.1, 31.8, 28.6, 28.2; HRMS (ESI) calculated $C_{26}H_{27}O_2NNa$ [M+Na$^+$] 408.1934 found 408.1929.

***tert*-Butyl 4-(thiophen-3-ylethynyl)piperidine-1-carboxylate (16)**

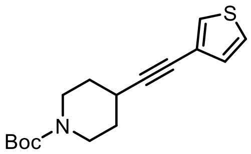

Following **GP2** using **1a** (37 mg, 0.12 mmol, 1.2 equiv) and **2n** (26 mg, 0.1 mmol, 1 equiv) gave **16** (76%) as a solid. Following **GP3** using **1b** (26 mg, 0.12 mmol, 1.2 equiv) gave **16** (32%). R_f 0.50 [*n*-pentane: EtOAc (15:1). ^{1}H NMR (600 MHz, $CDCl_3$) δ 7.36 (1H, dd, *J* = 3.0, 1.2 Hz), 7.24 (1H, dd, *J* = 5.0, 3.0 Hz), 7.07 (1H, dd, *J* = 5.0, 1.2 Hz), 3.79–3.66 (2H, m), 3.22 (2H, ddd, *J* = 13.5, 8.5, 3.4 Hz), 2.77 (1H, tt, *J* = 8.1, 4.0 Hz), 1.89–1.79 (2H, m), 1.71–1.60 (2H, m), 1.46 (9H, s). ^{13}C NMR (151 MHz, $CDCl_3$) δ 155.0, 130.1, 128.0, 125.3, 122.7, 91.5, 79.6, 77.1, 31.6, 28.6, 28.6, 27.8. HRMS (ESI) calculated $C_{16}H_{21}NO_2S$ [M $^+$] 291.1293 found 291.1293.

3-(Phenylethynyl)tetrahydro-2*H*-pyran (17)

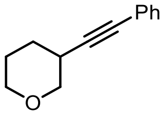

Following **GP2** using **1c** (14 µL, 0.12 mmol, 1.2 equiv) and **2a** (26 mg, 0.1 mmol, 1 equiv) gave **17** (74%) as an oil. R_f 0.52 [*n*-pentane: EtOAc (20:1)]. ^{1}H NMR (600 MHz, $CDCl_3$) δ 7.43–7.36 (2H, m), 7.32–7.18 (3H, m), 4.02 (1H, dd, *J*=11.0, 3.4 Hz), 3.90–3.81 (1H, m), 3.51–3.45 (1H, m), 3.47–3.43 (1H, m), 2.77 (1H, tt, *J*=9.6, 4.2 Hz), 2.16–2.09 (1H, m), 1.72–1.63 (3H, m); ^{13}C NMR (151 MHz, $CDCl_3$) δ 131.8, 128.3, 128.0, 123.6, 90.0, 82.0, 71.8, 68.3, 30.2, 29.7, 25.1; HRMS (EI) calculated $C_{13}H_{14}ONa$ [M+Na $^+$] 209.0934 found 209.0937.

***tert*-Butyl 3-(phenylethynyl)pyrrolidine-1-carboxylate (18)**

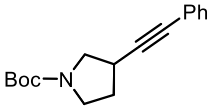

Following **GP2** using **1d** (30 mg, 0.12 mmol, 1.2 equiv) and **2a** (26 mg, 0.1 mmol, 1 equiv) gave **18** (90%) as an oil. R_f 0.50 [*n*-pentane: EtOAc (20:1)]. ^{1}H NMR (600 MHz, $CDCl_3$) δ 7.40–7.28 (5H, m), 3.74–3.15 (5H, m), 3.24–3.19 (1H, m), 2.06–1.98 (1H, m), 1.47 (9H, m); ^{13}C NMR (151 MHz, $CDCl_3$) δ 154.5, 131.8, 128.4, 128.1, 123.4, 89.6, 82.0, 79.6, 51.9, 45.1, 33.0, 30.7, 28.7. Data in accordance with the literature.[10]

***tert*-Butyl 3-(phenylethynyl)azetidine-1-carboxylate (19)**

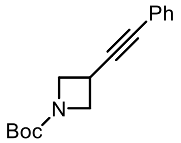

Following **GP2** using **1e** (21 μL, 0.12 mmol, 1.2 equiv) and **2a** (26 mg, 0.1 mmol, 1 equiv) gave **19** (71%) as an oil. Following **GP2** using **1f** (20 μL, 0.12 mmol, 1.2 equiv) gave **19** (74%). R_f 0.53 [*n*-pentane: EtOAc (10:1)]. ^{1}H NMR (600 MHz, $CDCl_3$) δ 7.43–7.39 (2H, m), 7.32–7.28 (3H, m), 4.21 (2H, t, *J*=8.5 Hz), 4.02 (2H, dd, *J*=8.2, 6.5 Hz), 3.53 (1H, tt, *J*=8.7, 6.4 Hz), 1.45 (9H, s); ^{13}C NMR (151 MHz, $CDCl_3$) δ 156.2, 131.7, 128.4, 128.3, 123.1, 89.4, 83.7, 79.9, 63.1, 28.5, 20.0; HRMS (ESI) calculated $C_{16}H_{19}NO_2Na$ [M+Na$^+$] 280.1313 found 280.1313.

3-(Phenylethynyl)oxetane (20)

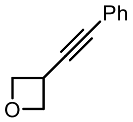

Following **GP2** using **1g** (10 μL, 0.12 mmol, 1.2 equiv) and **2a** (26 mg, 0.1 mmol, 1 equiv) gave **20** (55%) as an oil. R_f 0.50 [*n*-pentane: EtOAc (20:1)]. ^{1}H NMR (400 MHz, $CDCl_3$) δ 7.44–7.41 (2H, m), 7.32–7.30 (3H, m), 4.88 (2H, dd, *J* = 8.5, 5.5 Hz), 4.82 (2H, dd, *J* = 7.4, 5.6 Hz), 4.12–4.04 (1H, m); ^{13}C NMR (101 MHz, $CDCl_3$) δ 131.5, 128.3, 128.1, 123.0, 88.2, 84.1, 76.7, 26.5. Data in accordance with the literature.[11]

3-(Phenylethynyl)tetrahydro-2,6-methanofuro[3,2-*b*]furan-5(2*H*)-one (21)

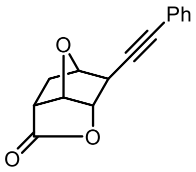

Following **GP2** using **1h** (27 mg, 0.12 mmol, 1.2 equiv) and **2a** (26 mg, 0.1 mmol, 1 equiv) gave **21** (88%) as an oil. R_f 0.61 [*n*-pentane: EtOAc (3:1)]. ^{1}H NMR (600 MHz, $CDCl_3$) δ 7.45–7.38 (2H, m), 7.34–7.27 (3H, m), 5.44 (1H, t, *J*=4.9 Hz), 4.89 (1H, d, *J*=4.9 Hz), 4.83 (1H, d, *J*=4.8 Hz), 2.95 (1H, s), 2.84–2.67 (1H, m), 2.26 (1H, ddd, *J*=13.2, 11.5, 4.9 Hz), 2.07 (1H, dd, *J*=13.3, 1.7 Hz); ^{13}C NMR (151 MHz, $CDCl_3$) δ 176.9, 131.9, 128.5, 128.4, 122.7, 86.5, 84.8, 83.7, 82.0, 81.7, 43.6, 39.0, 35.8; HRMS (EI) calculated $C_{15}H_{12}O_3$ [M$^+$] 240.0781 found 240.0780.

7-Phenyl-6-(phenylethynyl)hexahydro-2*H*-3,5-methanocyclopenta[*b*]furan-2-one (22)

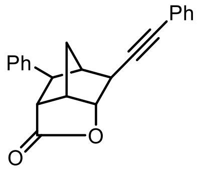

Following **GP2** using **1i** (34 mg, 0.12 mmol, 1.2 equiv) and **2a** (26 mg, 0.1 mmol, 1 equiv) gave **22** (88%) as an oil. R_f 0.40 [*n*-pentane: EtOAc (6:1)]. ^{1}H NMR (600 MHz, $CDCl_3$) δ 7.39 (2H, dd, *J*=6.6, 3.0 Hz), 7.35 (2H, t, *J*=7.7 Hz), 7.32–7.29 (3H, m), 7.27–7.22 (3H, m), 4.87 (1H, d, *J*=4.8 Hz), 3.33 (1H, t, *J*=4.5 Hz), 3.26 (1H, bs), 2.95–2.90 (1H, m), 2.86 (2H, bs), 2.15 (1H, d, *J*=11.4 Hz), 1.99 (1H, d, *J*=11.4 Hz); ^{13}C NMR (151 MHz, $CDCl_3$) δ 179.5, 141.7, 131.7, 128.9, 128.5, 128.4, 127.2, 127.0, 123.0, 88.2, 85.8, 84.2, 51.0, 49.2, 46.3, 46.1, 43.0, 33.7; HRMS (EI) calculated $C_{22}H_{18}O_2Na$ [M+Na$^+$] 337.1199 found 337.1191.

***rac*-(*1R,2R*)-2-(Phenylethynyl)-2,3-dihydro-1*H*-inden-1-ol (23)**

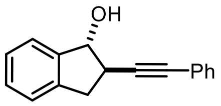

Following **GP2** using **1j** (21 mg, 0.12 mmol, 1.2 equiv) and **2a** (26 mg, 0.1 mmol, 1 equiv) gave **23** (56%) as an oil. R_f 0.39 [*n*-pentane: EtOAc (6:1)]. ^{1}H NMR (600 MHz, $CDCl_3$) δ 7.44 (2H, dd, *J*=6.6, 3.0 Hz), 7.42 (1H, dd, *J*=5.0, 3.6 Hz), 7.35–7.26 (5H, m), 7.24 (1H, dd, *J*=5.2, 3.6 Hz), 5.32 (1H, t, *J*=6.4 Hz), 3.37 (1H, dd, *J*=15.4, 8.2 Hz), 3.25–3.17 (1H, m), 3.04 (1H, dd, *J*=15.4, 9.1 Hz); ^{13}C NMR (151 MHz, $CDCl_3$) δ 143.3, 140.5, 131.9, 128.6, 128.4, 128.1, 127.3, 124.8, 124.1, 123.6, 90.6, 82.4, 82.2, 42.2, 37.3; HRMS (ESI) calculated $C_{17}H_{14}ONa$ [M+Na$^+$] 257.0937 found 257.0938.

(8-Chlorooct-1-yn-1-yl)benzene (24)

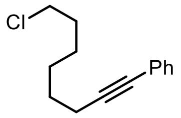

Following **GP3** using **1k** (18 μL, 0.12 mmol, 1.2 equiv) and **2a** (26 mg, 0.1 mmol, 1 equiv) gave **24** (51%) as an oil. R_f 0.52 [*n*-pentane]. ^{1}H NMR (600 MHz, $CDCl_3$) δ 7.41–7.36 (2H, m), 7.30 – 7.26 (3H, m), 3.57 (2H, t, *J*=6.7 Hz), 2.44 (2H, t, *J*=6.7 Hz), 1.84 (2H, p, *J*=6.8 Hz), 1.69–1.58 (4H, m); ^{13}C NMR (151 MHz, $CDCl_3$) δ 131.7, 128.3, 127.7, 124.0, 89.9, 81.1, 63.5, 45.1, 32.3, 28.1, 26.3, 19.4; HRMS (EI) calculated $C_{13}H_{15}Cl$ [M$^+$] 206.0857 found 206.0857.

8-Phenyloct-7-yn-1-ol (25)

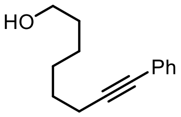

Following **GP3** using **1l** (16 μL, 0.12 mmol, 1.2 equiv) and **2a** (26 mg, 0.1 mmol, 1 equiv) gave **25** (49%) as an oil. R_f 0.50 [*n*-pentane:EtOAc]. ^{1}H NMR (600 MHz, $CDCl_3$) δ 7.43–7.34 (2H, m), 7.30–7.21 (3H, m), 3.64 (2H, t, *J* = 6.7 Hz), 2.41 (2H, t, *J* = 7.0 Hz), 1.68–1.53 (4H, m), 1.56–1.33 (4H, m); ^{13}C NMR (151 MHz, $CDCl_3$) δ 131.6, 128.2, 127.5, 124.0, 90.3, 80.7, 62.9, 32.7, 28.7, 28.7, 25.3, 19.4. Data in accordance with the literature.[12]

Dec-9-en-1-yn-1-ylbenzene (26)

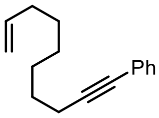

Following **GP3** using **1m** (20 μL, 0.12 mmol, 1.2 equiv) and **2a** (26 mg, 0.1 mmol, 1 equiv) gave **26** (89%) as an oil. R_f 0.50 [*n*-pentane]. ^{1}H NMR (600 MHz, $CDCl_3$) δ 7.42–7.38 (2H, m), 7.23–7.33 (3H, m), 5.81 (1H, ddt, *J* = 17.0, 10.1, 6.7 Hz), 5.05–5.00 (1H, m), 4.95 (1H, dm, *J* = 10.1 Hz), 2.40 (2H, t, *J* = 7.1 Hz), 2.06 (2H, q, *J* = 7.1 Hz), 1.60 (2H, p, *J* = 7.1 Hz), 1.30–1.53 (6H, m); ^{13}C NMR (151 MHz, $CDCl_3$) δ 139.2, 131.6, 128.3, 127.6, 124.1, 114.4, 90.5, 80.7, 33.9, 28.9, 28.9, 28.8, 28.7, 18.5. Data in accordance with the literature.[13]

2-((4-Phenylbut-3-yn-1-yl)oxy)tetrahydro-2H-pyran (27)

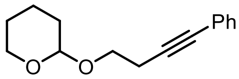

Following **GP3** using **1n** (18 μL, 0.12 mmol, 1.2 equiv) and **2a** (26 mg, 0.1 mmol, 1 equiv) gave **27** (70%) as an oil. R_f 0.45 [*n*-pentane:EtOAc (20:1)]. ^{1}H NMR (400 MHz, $CDCl_3$) δ 7.47–7.34 (2H, m), 7.33–7.21 (3H, m), 4.69 (1H, t, *J* = 3.3), 4.01–3.83 (2H, m), 3.65 (1H, dtd, *J* = 9.5, 7.1, 1.0), 3.56–3.48 (1H, m), 2.72 (2H, t, *J* = 7.2), 1.95–1.78 (1H, m), 1.78–1.68 (1H, m), 1.68–1.47 (4H, m); ^{13}C NMR (101 MHz, $CDCl_3$) δ 131.5, 128.2, 127.7, 123.7, 98.2, 86.1, 81.0, 65.1, 62.1, 30.6, 25.4, 20.9, 19.4. Data in accordance with the literature.[14]

(4,4-Dimethoxybut-1-yn-1-yl)benzene (28)

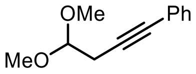

Following **GP3** using **1o** (14 µL, 0.12 mmol, 1.2 equiv) and **2a** (26 mg, 0.1 mmol, 1 equiv) gave **28** (70%) as an oil. R_f 0.50 [*n*-pentane: EtOAc (20:1)]. ^{1}H NMR (400 MHz, $CDCl_3$) δ 7.46–7.36 (2H, m), 7.32–7.19 (3H, m), 4.63 (1H, t, *J*=5.6 Hz), 3.42 (6H, s), 2.75 (2H, d, *J*=5.6 Hz); ^{13}C NMR (101 MHz, $CDCl_3$) δ 131.8, 128.3, 128.0, 123.7, 102.9, 85.0, 82.2, 53.7, 25.0; HRMS (APCI) calculated $C_{12}H_{15}O_2$ [M+H$^+$] 191.1072 found 191.1070.

Methyl (*S*)-2-((*tert*-butoxycarbonyl)amino)-5-phenylpent-4-ynoate (29)

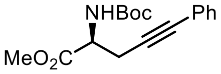

Following **GP2** using **1o** (33 mg, 0.12 mmol, 1.2 equiv) and **2a** (26 mg, 0.1 mmol, 1 equiv) gave **29** (54%) as an oil. R_f 0.49 [*n*-pentane: EtOAc (10:1)]. ^{1}H NMR (600 MHz, $CDCl_3$) δ 7.41–7.36 (2H, m), 7.31–7.26 (3H, m), 5.38 (1H, d, *J*=7.7 Hz), 4.56 (1H, dt, *J*=8.4, 4.3 Hz), 3.80 (3H, s), 2.98 (1H, dd, *J*=17.0, 4.5 Hz), 2.92 (1H, dd, *J*=17.0, 4.9 Hz), 1.46 (9H, s); ^{13}C NMR (151 MHz, $CDCl_3$) δ 171.5, 155.2, 131.9, 128.4, 128.3, 123.1, 84.0, 83.8, 80.3, 52.8, 52.4, 28.5, 24.1; HRMS (APCI) calculated $C_{17}H_{22}NO_4$ [M+H$^+$] 304.1549 found 304.1564.

***tert*-Butyl 4-(3-phenylprop-2-yn-1-yl)piperidine-1-carboxylate (30)**

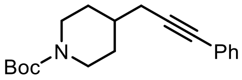

Following **GP2** using **1q** (33 mg, 0.12 mmol, 1.2 equiv) and **2a** (26 mg, 0.1 mmol, 1 equiv) gave **30** (95%) as an oil. Following **GP3** using **1r** (28 mg, 0.12 mmol, 1.2 equiv) gave **30** (64%). R_f 0.32 [*n*-pentane: EtOAc (10:1)]. ^{1}H NMR (400 MHz, $CDCl_3$) δ 7.43–7.39 (2H, m), 7.32–7.26 (3H, m), 4.15 (2H, bs), 2.74 (2H, t, *J* = 11.8 Hz), 2.39 (2H, d, *J* = 6.6 Hz), 1.83 (2H, d, *J* = 13.2 Hz), 1.76–1.73 (1H, m), 1.48 (9H, s), 1.33–1.27 (2H, m); ^{13}C NMR (101 MHz, $CDCl_3$) δ 155.0, 131.4, 128.0, 127.5, 123.6,87.8, 81.9, 79.2, 35.7, 31.4, 28.3, 26.2. Data in accordance with the literature.[15]

3-Methyl-3-(3-phenylprop-2-yn-1-yl)oxetane (31)

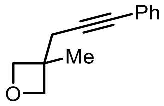

Following **GP2** using **1s** (15 µL, 0.12 mmol, 1.2 equiv) and **2a** (26 mg, 0.1 mmol, 1 equiv) gave **31** (91%) as an oil. R*f* 0.52 [*n*-pentane: EtOAc (20:1)]. ^{1}H NMR (600 MHz, $CDCl_3$) δ 7.42–7.36 (2H, m), 7.30–7.25 (3H, m), 4.57 (2H, d, *J*=5.8 Hz), 4.43 (2H, d, *J*=5.8 Hz), 2.72 (2H, s), 1.45 (3H, s); ^{13}C NMR (151 MHz, $CDCl_3$) δ 131.8, 128.4, 128.0, 123.6, 86.6, 82.5, 81.9, 39.3, 29.8, 23.6; HRMS (ESI) calculated $C_{13}H_{14}ONa$ [M+Na$^+$] 209.0942 found 209.0937.

Trimethyl(3-phenylprop-2-yn-1-yl)silane (32)

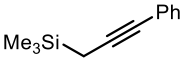

Following **GP2** using **1t** (18 µL, 0.12 mmol, 1.2 equiv) and **2a** (26 mg, 0.1 mmol, 1 equiv) gave **32** (46%) as an oil. R*f* 0.50 [*n*-pentane: EtOAc (30:1)]. Following **GP3** using **1u** (17 µL, 0.12 mmol, 1.2 equiv) gave **32** (50%). ^{1}H NMR (400 MHz, $CDCl_3$) δ 7.38–7.34 (m, 2H), 7.29–7.22 (m, 3H), 1.70 (s, 2H), 0.17 (s, 9H). Data in accordance with the literature.[16]

2,2-Dimethyl-5-phenylpent-4-yn-1-ol (33)

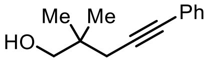

Following **GP2** using **1v** (15 µL, 0.12 mmol, 1.2 equiv) and **2a** (26 mg, 0.1 mmol, 1 equiv) gave **33** (19%) as an oil. R*f* 0.36 [*n*-pentane: EtOAc (10:1)]. ^{1}H NMR (400 MHz, $CDCl_3$) δ 7.39–7.41 (2H, m), 7.27–7.30 (3H, m), 3.49 (2H, d, *J* = 5.4 Hz), 2.38 (s, 2H), 1.59 (1H, bs), 1.04 (6H, s). Data in accordance with the literature.[17]

(*3r,5r,7r*)-1-(Phenylethynyl)adamantane (34)

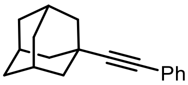

Following **GP2** using **1w** (26 mg, 0.12 mmol, 1.2 equiv) and **2a** (26 mg, 0.1 mmol, 1 equiv) gave **34** (70%) as an oil. R*f* 0.51 [*n*-pentane]. Following **GP3** using **1x** (22 mg, 0.12 mmol, 1.2 equiv) gave **34** (63%). ^{1}H NMR (400 MHz, $CDCl_3$) δ 7.36–7.40 (2H, m), 7.24–7.27 (3H, m), 1.99 (6H, bs), 1.96 (6H, bs), 1.72 (3H, bs); ^{13}C NMR (151 MHz, $CDCl_3$) δ 131.6, 128.1, 127.4, 124.1, 98.4, 79.4, 42.9, 36.4, 30.1, 28.1. Data in accordance with the literature.[18]

Methyl 3-(phenylethynyl)bicyclo[1.1.1]pentane-1-carboxylate (35)

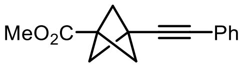

Following **GP2** using **1y** (25 mg, 0.12 mmol, 1.2 equiv) and **2a** (26 mg, 0.1 mmol, 1 equiv) gave **35** (61%) as an oil. R_f 0.50 [*n*-pentane: EtOAc (20:1)]. ^{1}H NMR (600 MHz, $CDCl_3$) δ 7.46–7.35 (2H, m), 7.33–7.18 (3H, m), 3.69 (3H, s), 2.40 (6H, s); ^{13}C NMR (151 MHz, $CDCl_3$) δ 170.0, 131.9, 128.4, 128.4, 122.9, 87.8, 80.7, 56.0, 51.9, 40.0, 29.9; HRMS (APCI) calculated $C_{15}H_{15}O_2$ [M+H $^+$] 227.1072 found 227.1082.

Methyl 4-(phenylethynyl)cubane-1-carboxylate (36)

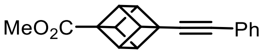

Following **GP2** using **1z** (29 mg, 0.12 mmol, 1.2 equiv) and **2a** (26 mg, 0.1 mmol, 1 equiv) gave **36** (79%) as a solid. R_f 0.50 [*n*-pentane: EtOAc (20:1)]. ^{1}H NMR (400 MHz, $CDCl_3$) δ 7.42–7.35 (2H, m), 7.32–7.27 (3H, m), 4.28–4.21 (3H, m), 4.19–4.14 (3H, m), 3.72 (3H, s); ^{13}C NMR (101 MHz, $CDCl_3$) δ 172.3, 131.5, 128.4, 128.0, 123.7, 89.8, 88.7, 56.2, 51.7, 49.7, 47.1, 46.7, 29.9; HRMS (EI) calculated $C_{18}H_{14}O_2$ [M $^+$] 262.0988 found 262.0987.

***rac*-(*3S,3aR,6aS*)-3-(3-Phenylprop-2-yn-1-yl)hexahydrofuro[2,3-b]furan (37)**

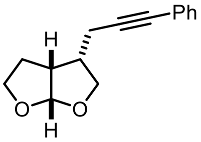

Following **GP2** using **1aa** (25 mg, 0.12 mmol, 1.2 equiv) and **2a** (26 mg, 0.1 mmol, 1 equiv) gave **37** (60%) as an oil. Following **GP3** using **1ab** (21 mg, 0.12 mmol, 1.2 equiv) gave **37** (53%). R_f 0.46 [*n*-pentane: EtOAc (4:1)]. ^{1}H NMR (600 MHz, $CDCl_3$) δ 7.43–7.34 (2H, m), 7.33–7.23 (3H, m), 5.77 (1H, d, *J*=4.9 Hz), 4.07 (1H, dd, *J* = 8.4, 7.6 Hz), 3.98–3.79 (2H, m), 3.56 (1H, dd, *J*=11.1, 8.7 Hz), 2.96 (1H, tt, *J*=9.7, 5.1 Hz), 2.7– 2.63 (1H, m), 2.53 (1H, dd, *J*=17.1, 1.5 Hz), 2.50 (1H, dd, *J*=17.0, 3.0 Hz), 2.04–1.90 (2H, m); ^{13}C NMR (151 MHz, $CDCl_3$) δ 131.5, 128.3, 127.9, 123.4, 109.8, 87.2, 81.6, 72.0, 69.3, 45.4, 41.4, 25.3, 18.0; HRMS (ESI) calculated $C_{15}H_{16}O_2Na$ [M+Na$^+$] 251.1043 found 251.1045.

tert-Butyl (_1R,3s,5S_)-3-(phenylethynyl)-8-azabicyclo[3.2.1]octane-8-carboxylate (38)

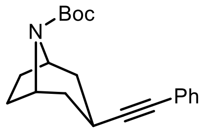

Following **GP2** using **1ac** (34 mg, 0.12 mmol, 1.2 equiv) and **2a** (26 mg, 0.1 mmol, 1 equiv) gave **38** (87%) as an oil. R_f 0.60 [*n*-pentane: EtOAc (10:1)]. ^{1}H NMR (600 MHz, $CDCl_3$, rotamers) δ 7.39–7.32 (2H, m), 7.29–7.20 (3H, m), 4.31–4.12 (2H, m), 3.07–2.96 (1H, m), 2.02–1.96 (2H, m), 1.96–1.86 (1H, m), 1.86–1.80 (3H, m), 1.66 (2H, q, *J*=6.9 Hz), 1.48 (9H, s); ^{13}C NMR (151 MHz, $CDCl_3$, rotamers) δ 153.2, 131.6, 128.2, 127.7, 123.6, 92.4, 80.6, 79.3, 53.5, 52.7, 37.7, 37.0, 28.5, 28.2, 27.6, 21.9; HRMS (EI) calculated $C_{20}H_{25}NO_2$ [M^+] 311.1878 found 311.1882.

(_3aR,4R,5R,6aS_)-2-Oxo-4-(3-phenylprop-2-yn-1-yl)hexahydro-2_H_-cyclopenta[_b_]furan-5-yl [1,1'-biphenyl]-4-carboxylate (39)

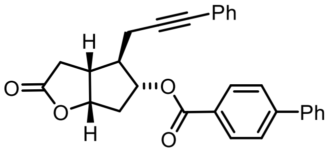

Following **GP2** using **1ad** (46 mg, 0.12 mmol, 1.2 equiv) and **2a** (26 mg, 0.1 mmol, 1 equiv) gave **39** (74%) as a solid. R_f 0.50 [*n*-pentane: EtOAc (2:1)]. ^{1}H NMR (600 MHz, $CDCl_3$) δ 8.08 (2H, d, *J*=8.3 Hz), 7.66 (2H, d, *J*=8.3 Hz), 7.62 (2H, d, *J*=7.4 Hz), 7.47 (2H, t, *J*=7.6 Hz), 7.41–7.39 (1H, m), 7.39–7.36 (2H, m), 7.32–7.27 (3H, m), 5.43–5.35 (1H, m), 5.12 (1H, t, *J*=6.1 Hz), 3.03–2.93 (2H, m), 2.74–2.69 (2H, m), 2.68–2.65 (1H, m), 2.62 (1H, dd, *J*=17.0, 7.1 Hz), 2.48–2.44 (1H, m), 2.35 (1H, dd, *J*=15.6, 3.7 Hz); ^{13}C NMR (151 MHz, $CDCl_3$) δ 176.7, 166.0, 146.2, 140.1, 131.7, 130.4, 130.3, 129.1, 128.5, 128.4, 128.3, 127.4, 127.3, 123.1, 86.0, 84.3, 83.1, 79.4, 51.1, 42.6, 38.3, 36.0, 22.5; HRMS (APCI) calculated $C_{29}H_{25}O_4$ [$M+H^+$] 437.1753 found 437.1751.

(_3aR,5R,5aS,8aS,8bR_)-2,2,7,7-Tetramethyl-5-(3-phenylprop-2-yn-1-yl)tetrahydro-5_H_-bis([1,3]dioxolo)[4,5-_b_:4',5'-_d_]pyran (40)

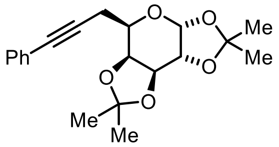

Following **GP2** using **1ae** (37 mg, 0.12 mmol, 1.2 equiv) and **2a** (26 mg, 0.1 mmol, 1 equiv) gave **40** (52%) as a solid. R_f 0.48 [*n*-pentane: EtOAc (20:1)]. ^{1}H NMR (600 MHz, $CDCl_3$) δ 7.43–7.37 (2H, m), 7.29–7.26 (3H, m), 5.54 (1H, d, *J*=5.0 Hz), 4.65 (1H, dd, *J*=7.9, 2.3 Hz),

4.42 (1H, dd, *J*=7.9, 1.7 Hz), 4.32 (1H, dd, *J*=5.0, 2.3 Hz), 4.00 (1H, ddd, *J*=8.3, 6.0, 1.5 Hz), 2.82 (1H, dd, *J*=16.4, 8.9 Hz), 2.72 (1H, dd, *J*=16.4, 6.0 Hz), 1.57 (3H, s), 1.48 (3H, s), 1.38 (3H, s), 1.35 (3H, s); ^{13}C NMR (151 MHz, $CDCl_3$) δ 131.8, 128.3, 127.9, 123.8, 109.4, 108.8, 96.7, 85.8, 82.1, 71.5, 71.0, 70.8, 67.0, 26.3, 26.1, 25.1, 24.6, 21.5; HRMS (EI) calculated $C_{20}H_{24}O_5$ [M $^+$] 344.1618 found 344.1618.

(*2R,3R,4R,5S,6R*)-2-(Acetoxymethyl)-6-(phenylethynyl)tetrahydro-2*H*-pyran-3,4,5-triyl triacetate (41)

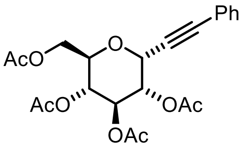

Following **GP2** using **1af** (49 mg, 0.12 mmol, 1.2 equiv) and **2a** (26 mg, 0.1 mmol, 1 equiv) gave **42** (65%) as a solid. R_f 0.49 [*n*-pentane: EtOAc (5:2)]. ^{1}H NMR (600 MHz, $CDCl_3$) δ 7.56–7.46 (2H, m), 7.43–7.33 (3H, m), 5.57 (1H, t, *J*=9.7 Hz), 5.26 (1H, d, *J*=5.8 Hz), 5.08 (1H, t, *J*=9.6 Hz), 5.01 (1H, dd, *J*=10.1, 5.8 Hz), 4.35–4.25 (2H, m), 4.19–4.07 (1H, m), 2.10 (3H, s), 2.09 (3H, s), 2.04 (6H, bs); ^{13}C NMR (151 MHz, $CDCl_3$) δ 170.9, 170.3, 170.1, 169.8, 132.2, 129.4, 128.6, 121.6, 90.8, 81.4, 71.3, 71.0, 70.1, 68.5, 66.2, 62.1, 20.9 (3C), 20.8; HRMS (ESI) calculated $C_{22}H_{24}O_9Na$ [M+Na$^+$] 455.1313 found 455.1312.

(*3aR,5S,6R,6aR*)-5-((*S*)-2,2-Dimethyl-1,3-dioxolan-4-yl)-2,2-dimethyl-6-(phenylethynyl)tetrahydrofuro[2,3-*d*][1,3]dioxole (42)

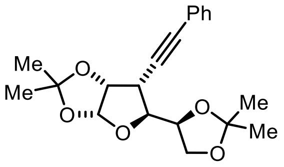

Following **GP2** using **1ag** (37 mg, 0.12 mmol, 1.2 equiv) and **2a** (26 mg, 0.1 mmol, 1 equiv) gave **42** (66%) as an oil. R_f 0.50 [*n*-pentane: EtOAc (20:1)]. ^{1}H NMR (600 MHz, $CDCl_3$) δ 7.46–7.38 (2H, m), 7.33–7.28 (3H, m), 5.95 (1H, d, *J*=3.5 Hz), 4.83 (1H, d, *J*=3.5 Hz), 4.44 (1H, dt, *J*=8.3, 5.7 Hz), 4.17 (1H, d, *J*=8.5 Hz), 4.16 (1H, dd, *J*=8.5, 1.5 Hz), 4.08 (1H, dd, *J*=8.6, 5.2 Hz), 3.43 (1H, d, *J*=4.2 Hz), 1.54 (3H, s), 1.45 (3H, s), 1.37 (3H, s), 1.34 (3H, s); ^{13}C NMR (151 MHz, $CDCl_3$) δ 131.7, 128.3, 128.3, 122.7, 112.1, 109.4, 105.4, 85.9, 85.2, 83.9, 79.9, 74.8, 67.4, 41.0, 26.9, 26.7, 26.2, 25.3; HRMS (ESI) calculated $C_{20}H_{25}O_5$ [M+H$^+$] 345.1697 found 345.1696.

(*5S,8R,9S,10S,13S,14S*)-10,13-Dimethyl-3-(phenylethynyl)hexadecahydro-17*H*-cyclopenta[*a*]phenanthren-17-one (43)

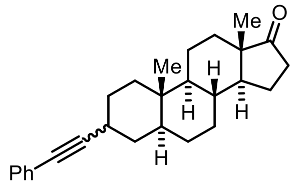

Following **GP2** using **1ag** (40 mg, 0.12 mmol, 1.2 equiv) and **2a** (26 mg, 0.1 mmol, 1 equiv) gave **43** (63%) as a mixture of stereoisomers (1:1).

Data for isomer A: ^{1}H NMR (600 MHz, $CDCl_3$) δ 7.44–7.40 (2H, m), 7.30–7.26 (3H, m), 3.02 (1H, p, *J*=3.9 Hz), 2.43 (1H, dd, *J*=19.4, 8.5 Hz), 2.11–2.01 (1H, m), 1.93 (1H, ddd, *J*=13.7, 8.2, 6.1 Hz), 1.84–1.75 (2H, m), 1.73 (2H, dt, *J*=9.5, 3.8 Hz), 1.71–1.67 (1H, m), 1.65 (1H, dt, *J*=12.3, 3.3 Hz), 1.59–1.53 (4H, m), 1.50–1.43 (3H, m), 1.34–1.22 (5H, m), 1.05 (1H, qd, *J*=12.7, 4.6 Hz), 0.86 (3H, s), 0.83 (3H, s); ^{13}C NMR (151 MHz, $CDCl_3$) δ 221.6, 131.7, 128.3, 127.6, 124.3, 94.3, 81.4, 54.6, 51.6, 48.0, 41.9, 36.5, 36.0, 35.2, 34.4, 33.4, 31.7, 30.9, 28.4, 28.0, 27.0, 21.9, 20.2, 14.0, 12.0; HRMS (EI) calculated $C_{27}H_{34}O$ [M $^{+}$] 374.2604 found 374.2603.

Data for isomer B: ^{1}H NMR (600 MHz, $CDCl_3$) δ 7.43–7.32 (2H, m), 7.30–7.23 (3H, m), 4.20–4.07 (1H, m), 2.47–2.39 (2H, m), 2.28–2.22 (1H, m), 2.15 (1H, qd, *J*=13.3, 4.0 Hz), 2.07 (2H, ddd, *J*=14.7, 9.5, 5.0 Hz), 2.03–1.97 (1H, m), 1.95–1.88 (2H, m), 1.84–1.76 (3H, m), 1.63–1.50 (5H, m), 1.34–1.17 (5H, m), 1.06 (1H, dd, *J*=13.3, 3.9 Hz), 0.88 (3H, s), 0.85 (3H, s); ^{13}C NMR (151 MHz, $CDCl_3$) δ 221.3, 131.7, 128.3, 127.6, 124.1, 94.5, 80.2, 54.6, 51.5, 49.6, 47.9, 46.5, 42.9, 41.2, 38.2, 36.5, 35.9, 31.6, 30.8, 30.0, 28.1, 21.9, 20.3, 13.9, 12.4; HRMS (EI) calculated $C_{27}H_{34}O$ [M $^{+}$] 374.2604 found 374.2603.

***tert*-Butyl 4-(((*8R,9S,13S,14S,17S*)-17-hydroxy-3-methoxy-13-methyl-7,8,9,11,12,13,14,15,16,17-decahydro-6*H*-cyclopenta[*a*]phenanthren-17-yl)ethynyl)piperidine-1-carboxylate (44)**

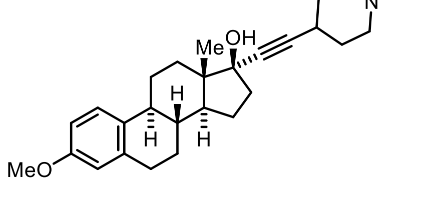

Following **GP2** using **1a** (27 mg, 0.12 mmol, 1.2 equiv) and **2o** (46 mg, 0.1 mmol, 1 equiv) gave **44** (27%) as an oil. R_f 0.43 [*n*-pentane: EtOAc (4:1)]. ^{1}H NMR (600 MHz, $CDCl_3$) 7.22 (1H, d, *J*=8.7 Hz), 6.72 (1H, dd, *J*=8.7, 2.3 Hz), 6.63 (1H, d, *J*=2.3 Hz), 3.78 (3H, s), 3.70–

3.59 (2H, m), 3.28–3.23 (2H, ddd, J=13.2, 8.5, 3.3 Hz), 2.88–2.81 (2H, m), 2.66 (1H, tt, *J*=8.0, 3.9 Hz), 2.36 (1H, dd, *J*=13.0, 2.8 Hz), 2.26 (1H, ddd, *J*=13.9, 9.4, 5.7 Hz), 2.17 (1H, td, *J*=11.6, 3.6 Hz), 2.07–1.96 (2H, m), 1.91–1.83 (2H, m), 1.82–1.65 (7H, m), 1.57 (3H, s), 1.45 (9H, s), 1.42–1.30 (3H, m); ^{13}C NMR (151 MHz, $CDCl_3$) δ 162.7, 157.6, 138.1, 132.7, 126.5, 113.9, 111.6, 87.7, 80.0, 74.1, 55.3, 49.6, 47.3, 43.6, 39.5, 39.1, 37.4, 32.9, 30.0, 28.5, 27.8, 27.4, 26.5, 22.9, 12.8; HRMS (EI) calculated $C_{31}H_{43}NO_4$ [M $^+$] 493.3192 found 493.3191.

***tert*-Butyl (*E*)-4-styrylpiperidine-1-carboxylate (45)**

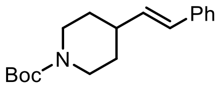

Following **GP2** using **1a** (37 mg, 0.12 mmol, 1.2 equiv) and (*E*)-(2-(phenylsulfonyl)vinyl)benzene (25 mg, 0.1 mmol, 1 equiv) gave **45** (66%). ^{1}H NMR (600 MHz, $CDCl_3$) δ 7.36–7.33 (2H, m), 7.31–7.28 (2H, m), 7.22–7.18 (1H, m), 6.39 (1H, dd, *J*= 16.0, 1.2 Hz), 6.15 (1H, dd, *J*=16.0, 6.9 Hz), 4.13 (2H, d, *J*=12.9 Hz), 2.78 (2H, t, *J*=12.5 Hz), 2.33–2.24 (1H, m), 1.79–1.73 (2H, m), 1.48 (9H, s), 1.44–1.33 (2H, m). Data in accordance ith the literature.[19]

***tert*-Butyl 4-cyanopiperidine-1-carboxylate (46)**

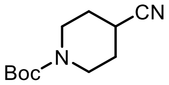

Following **GP2** using **1a** (37 mg, 0.12 mmol, 1.2 equiv) and *p*-toluenesulfonyl cyanide (20 mg, 0.1 mmol, 1 equiv) gave **46** (50%). ^{1}H NMR (600 MHz, $CDCl_3$) δ 3.65 (2H, ddd, *J*=13.8, 7.1, 3.8 Hz), 3.33 (2H, ddd, *J*=3.9, 7.8, 3.7 Hz), 2.79 (1H, tt, *J*=8.0, 4.2 Hz), 1.94–1.84 (2H, m), 1.83–1.71 (2H, m), 1.46 (9H, s). Data in accordance ith the literature.[20]

***tert*-Butyl 4-chloropiperidine-1-carboxylate (47)**

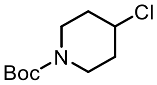

Following **GP2** using **1a** (37 mg, 0.12 mmol, 1.2 equiv) and *p*-toluenesulfonyl chloride (19 mg, 0.1 mmol, 1 equiv) gave **47** (26%). ^{1}H NMR (600 MHz, $CDCl_3$) δ 4.20 (1H, dq, *J*=7.7, 3.8 Hz), 3.72 (2H, ddd, *J*=13.7, 7.1, 3.7 Hz), 3.31 (2H, ddd, *J*=13.7, 7.8, 3.6 Hz), 2.05–1.99 (2H, m), 1.80 (2H, dtd, *J*=13.3, 7.7, 3.7 Hz), 1.48 (9H, s). Data in accordance ith the literature.[21]

9. Copies of NMR spectra for new compounds

2g – ^{1}H NMR (600 MHz, $CDCl_3$)

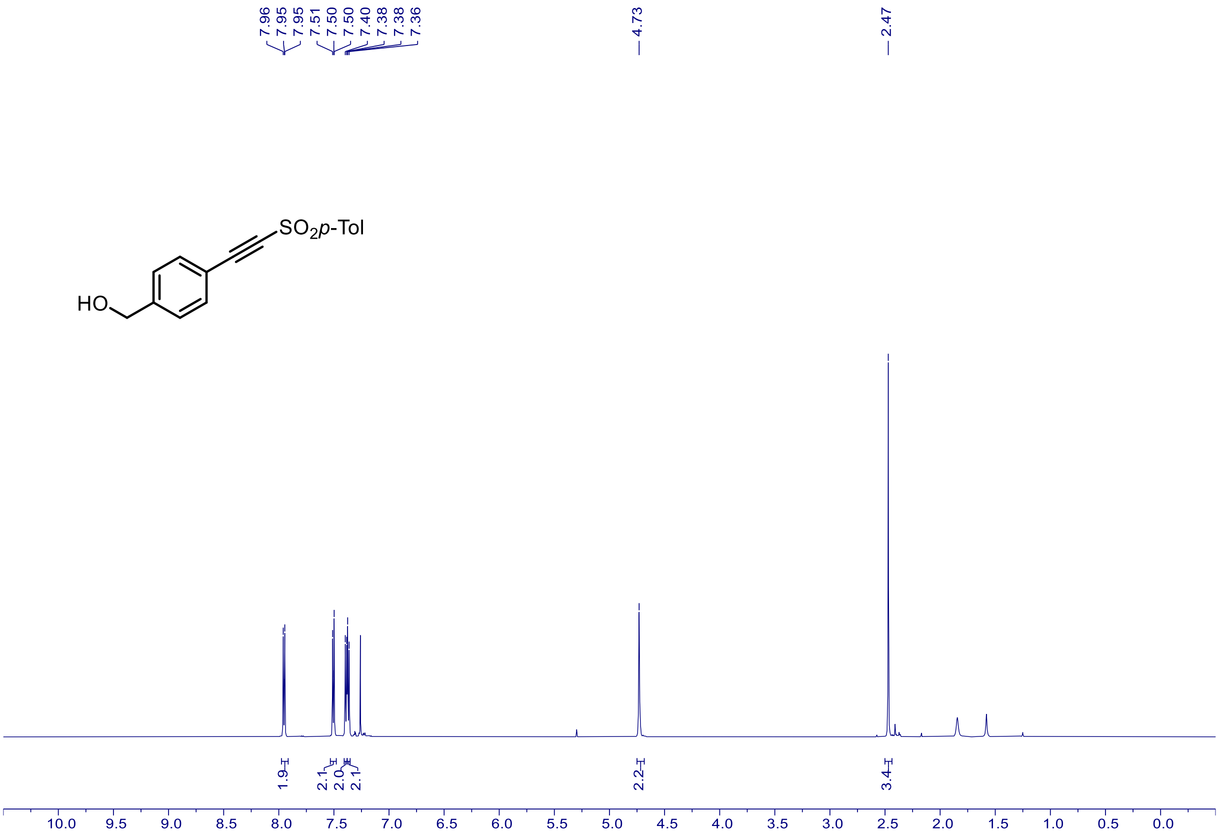

2g – ^{13}C NMR (151 MHz, $CDCl_3$)

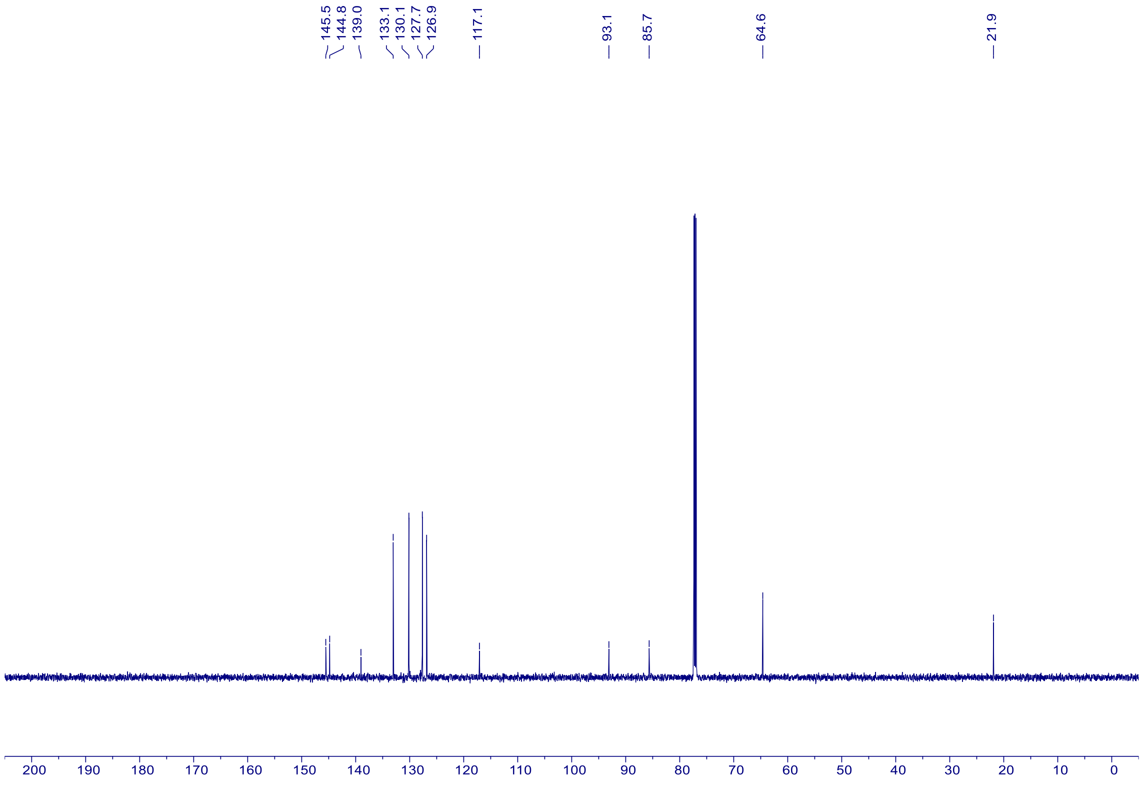

2l – ^{1}H NMR (600 MHz, $CDCl_3$)

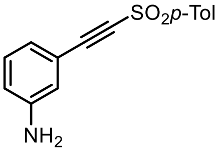

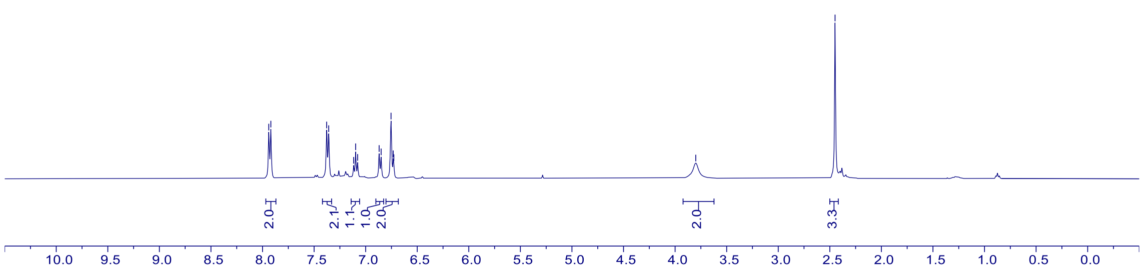

2l – ^{13}C NMR (151 MHz, $CDCl_3$)

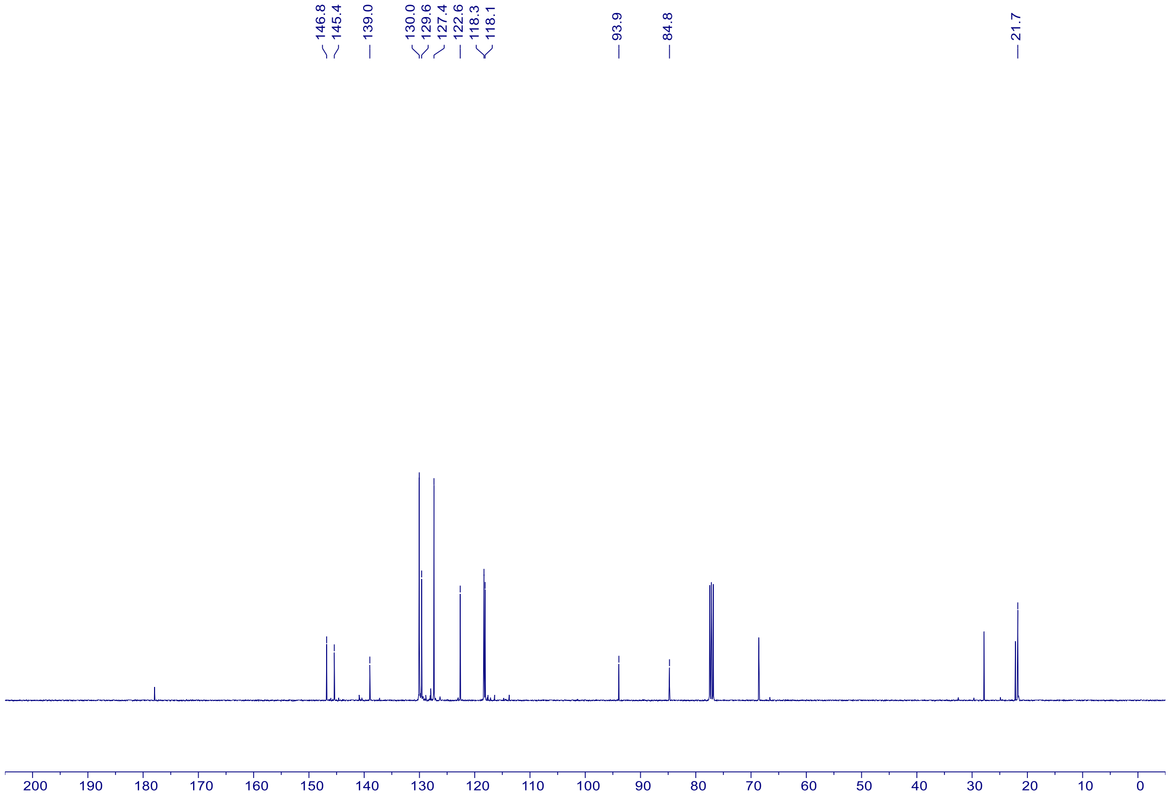

2m – ^{1}H NMR (600 MHz, $CDCl_3$)

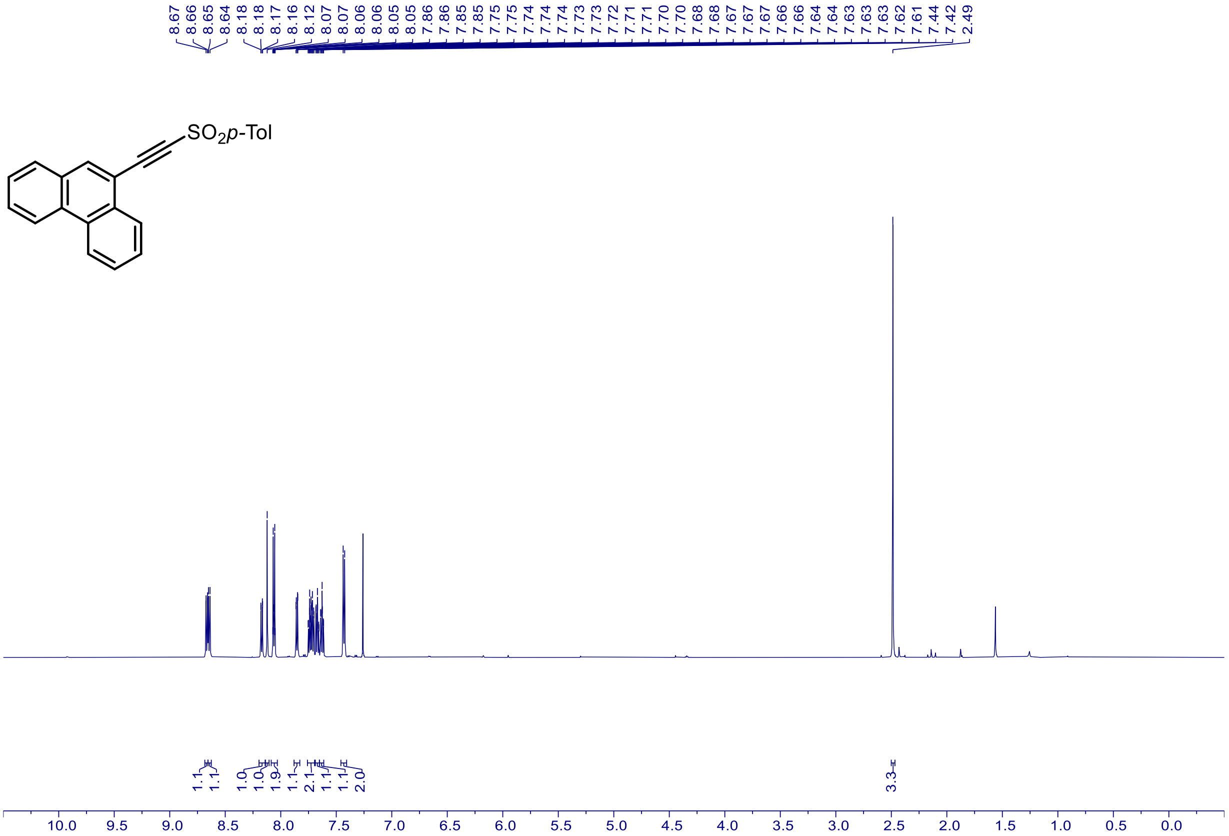

2m – ^{13}C NMR (151 MHz, $CDCl_3$)

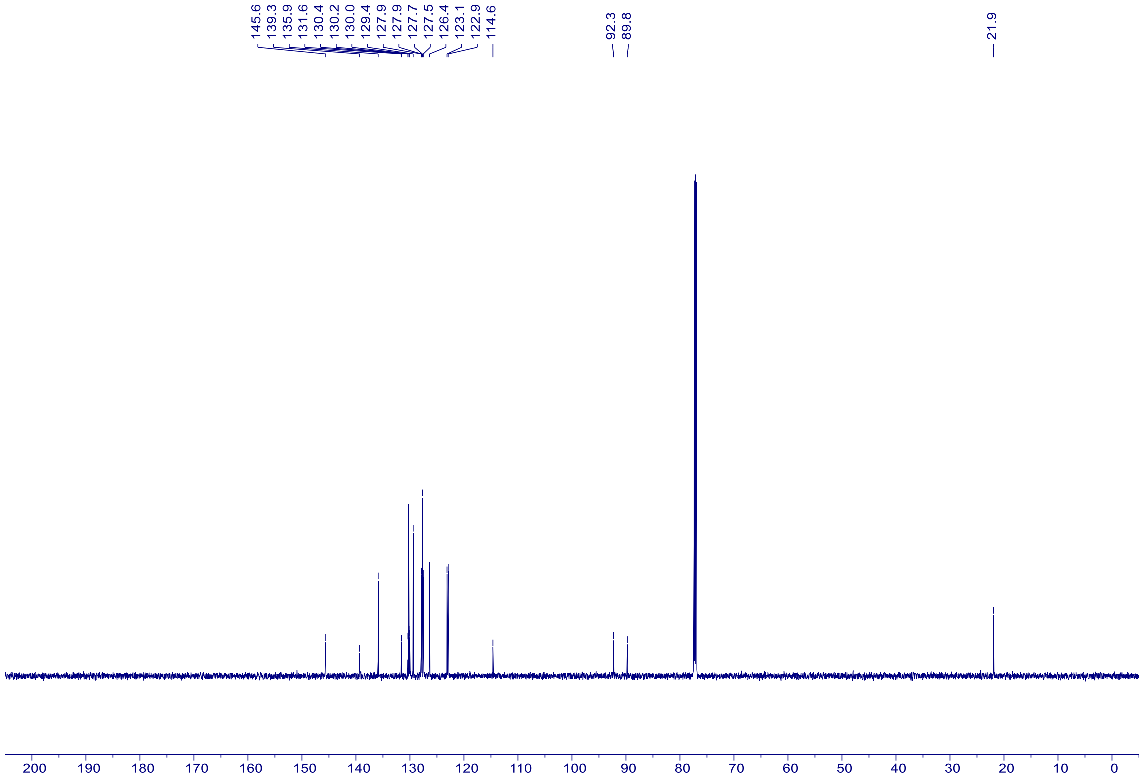

2o – ^{1}H NMR (600 MHz, $CDCl_3$)

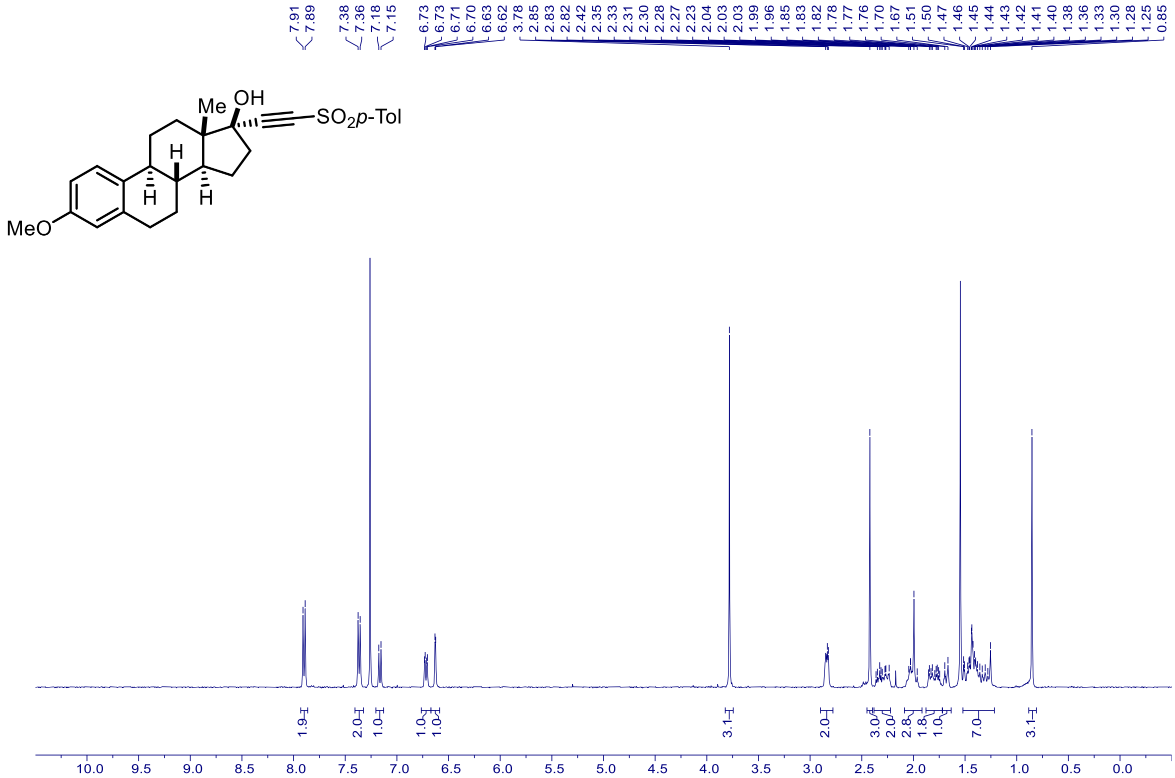

2o – ^{13}C NMR (151 MHz, $CDCl_3$)

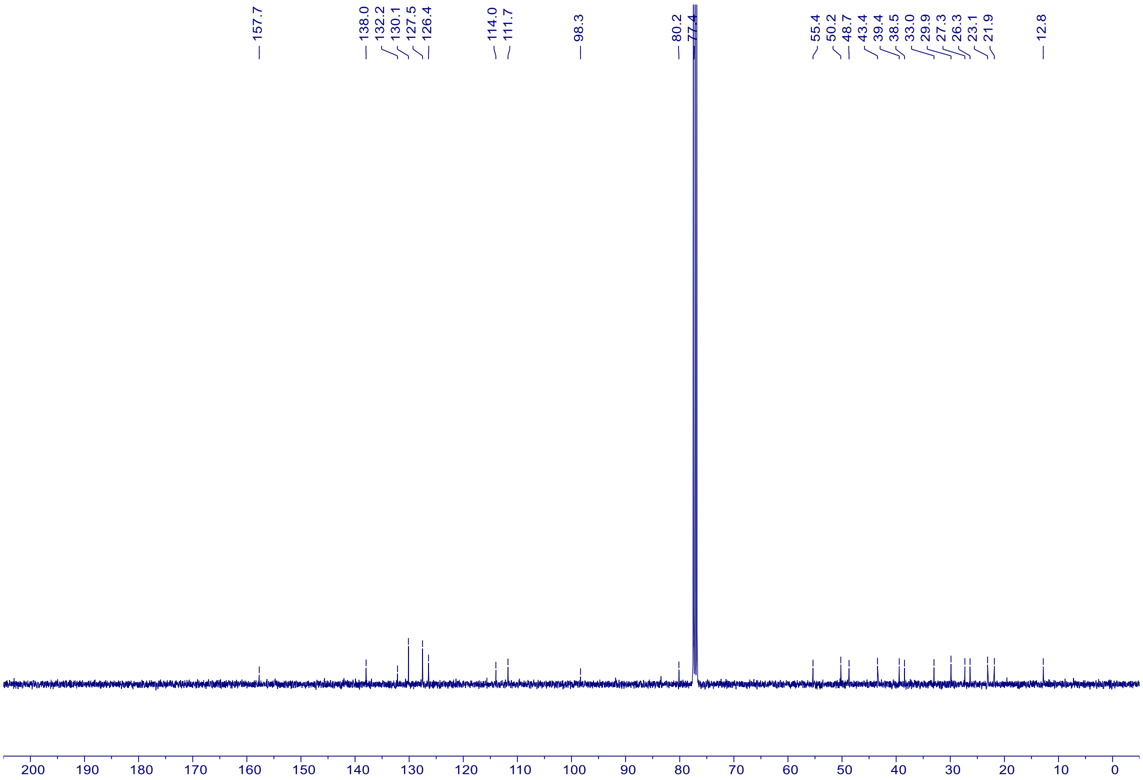

5 – ^{1}H NMR (600 MHz, $CDCl_3$)

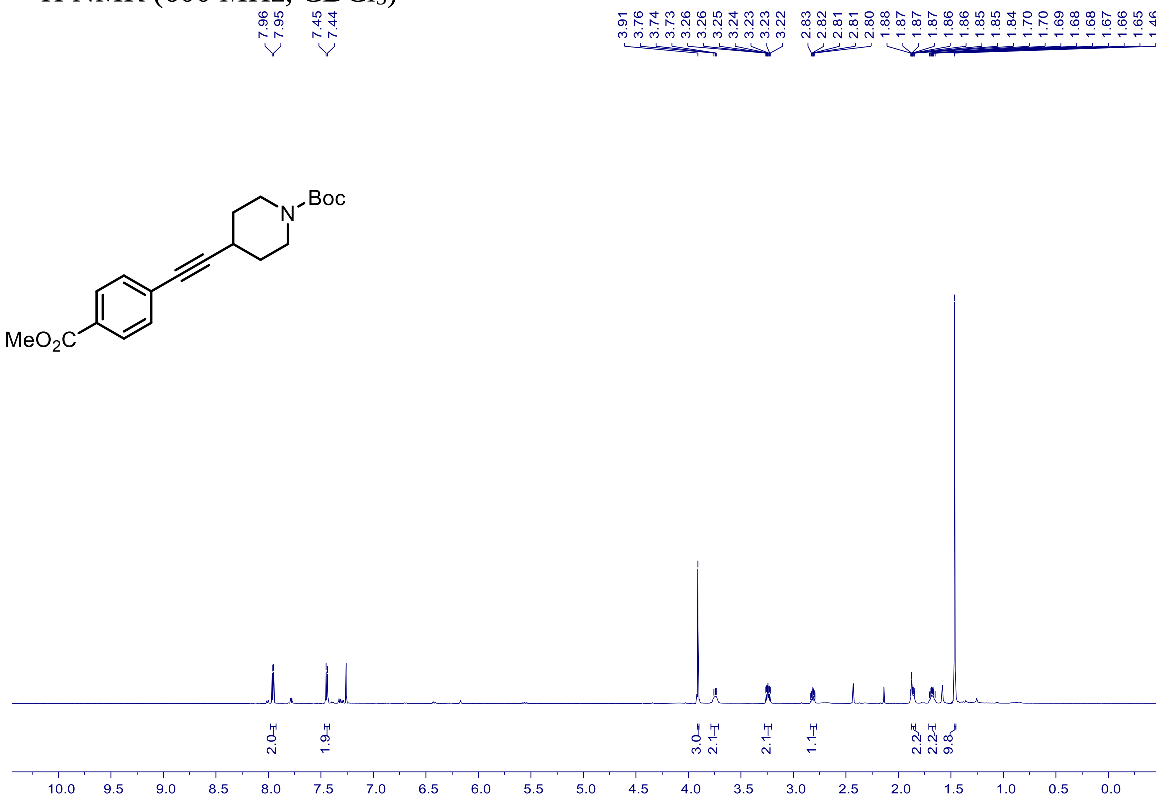

5 – ^{13}C NMR (151 MHz, $CDCl_3$)

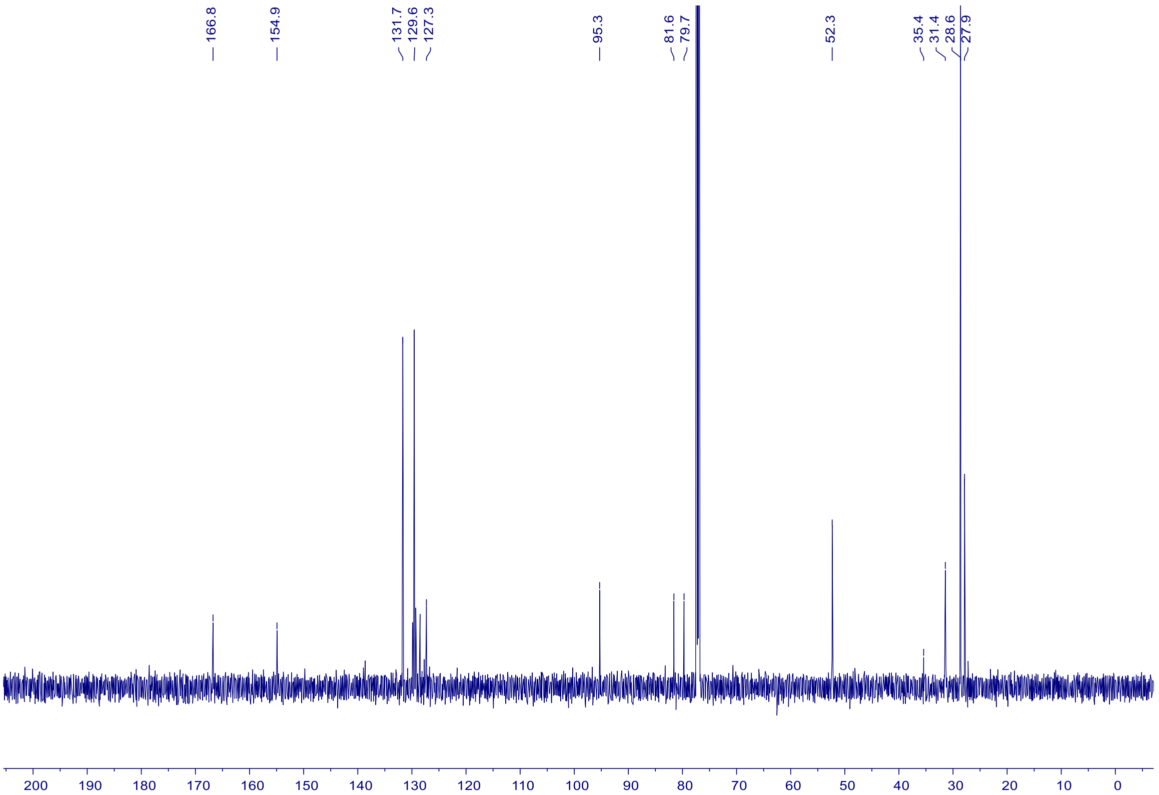

6 – ^{1}H NMR (600 MHz, CDCl$_3$)

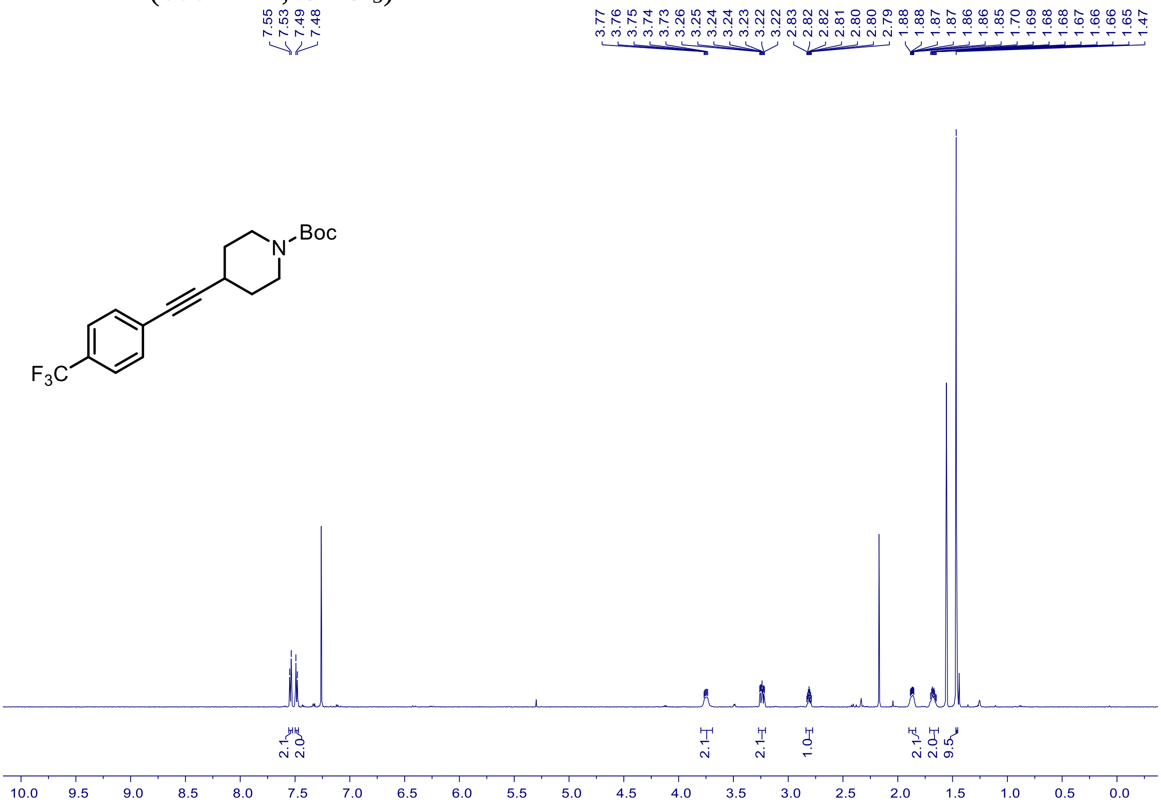

6 – ^{13}C NMR (151 MHz, CDCl$_3$)

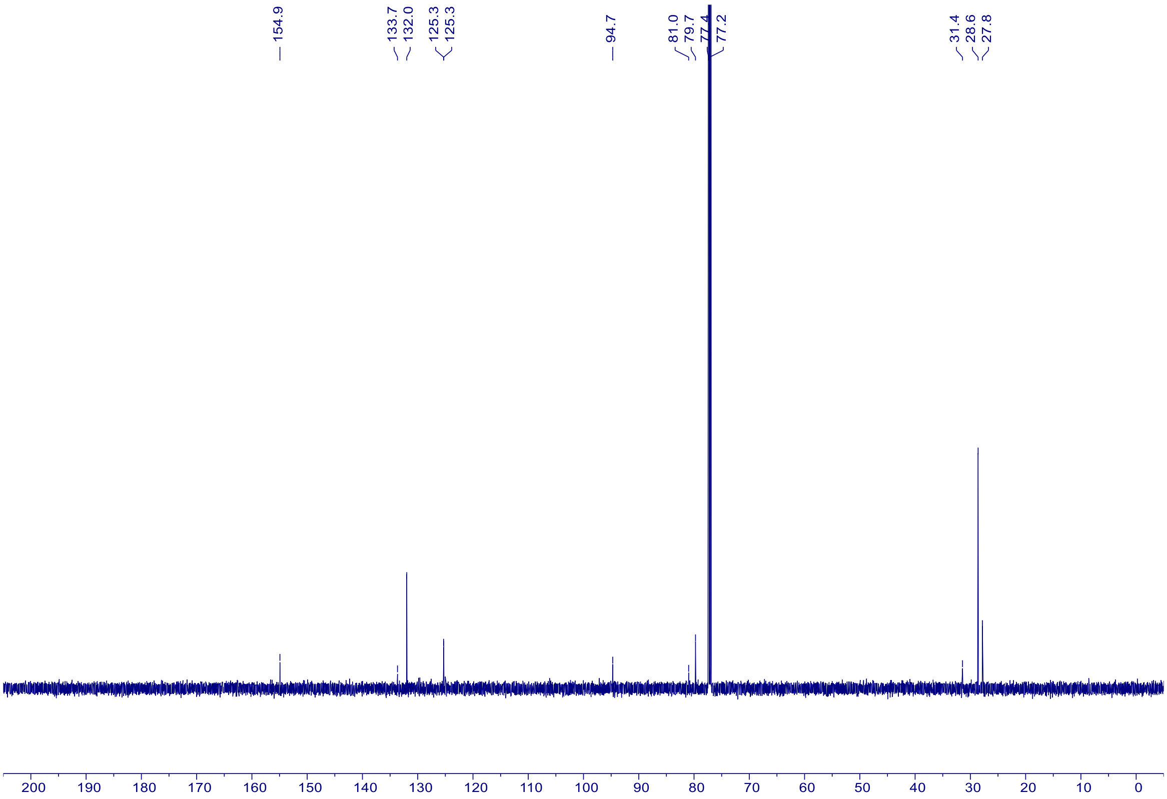

6 – ^{19}F NMR (565 MHz, $CDCl_3$)

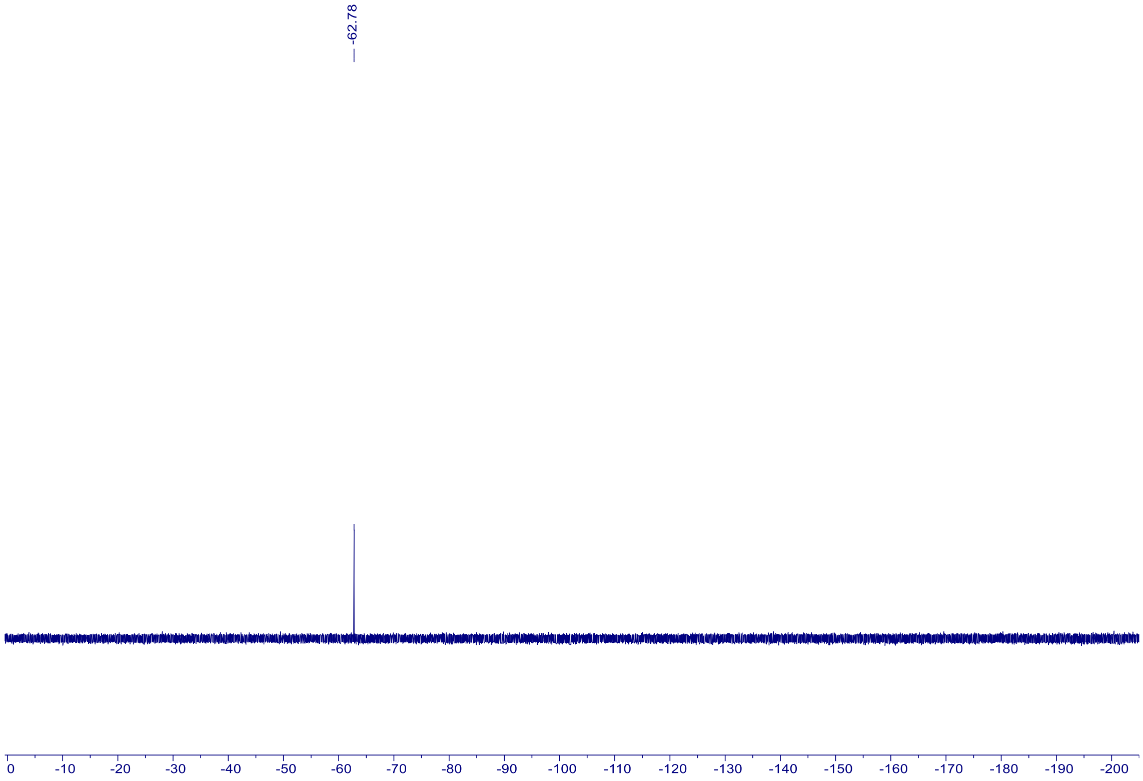

7 – ^{1}H NMR (600 MHz, $CDCl_3$)

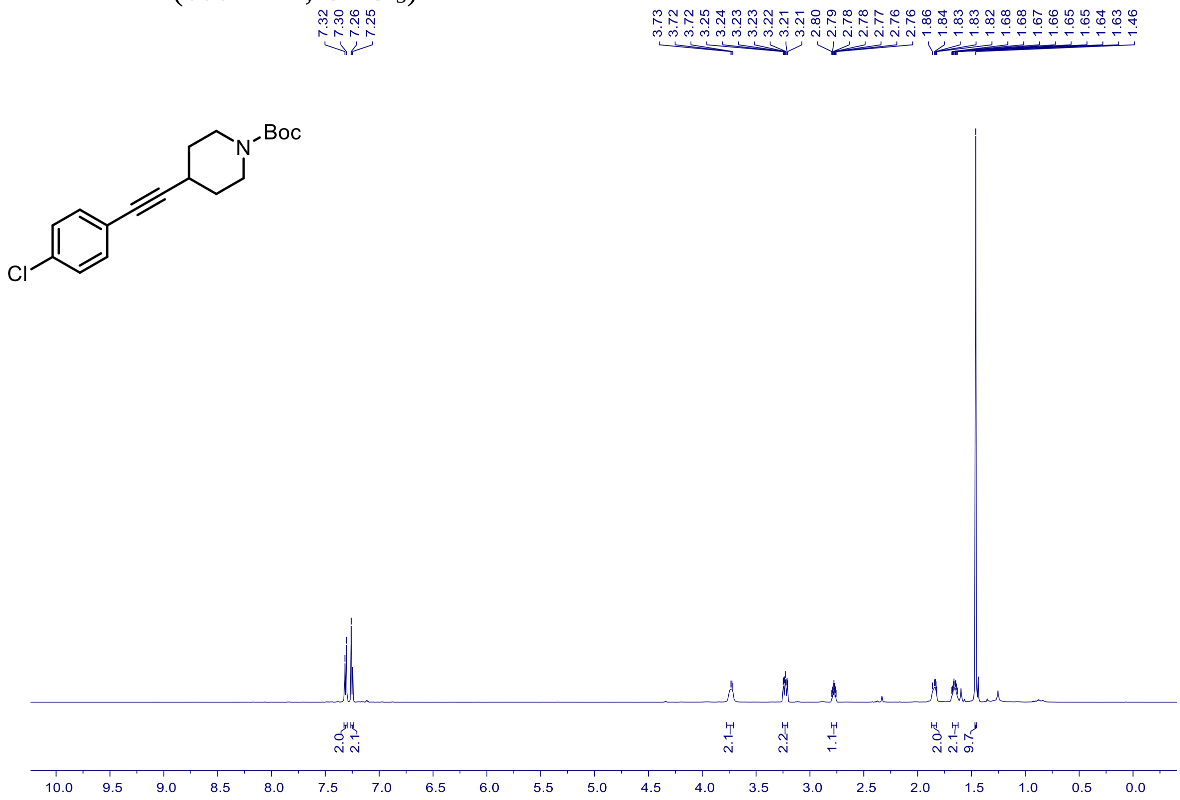

7 – ^{13}C NMR (151 MHz, $CDCl_3$)

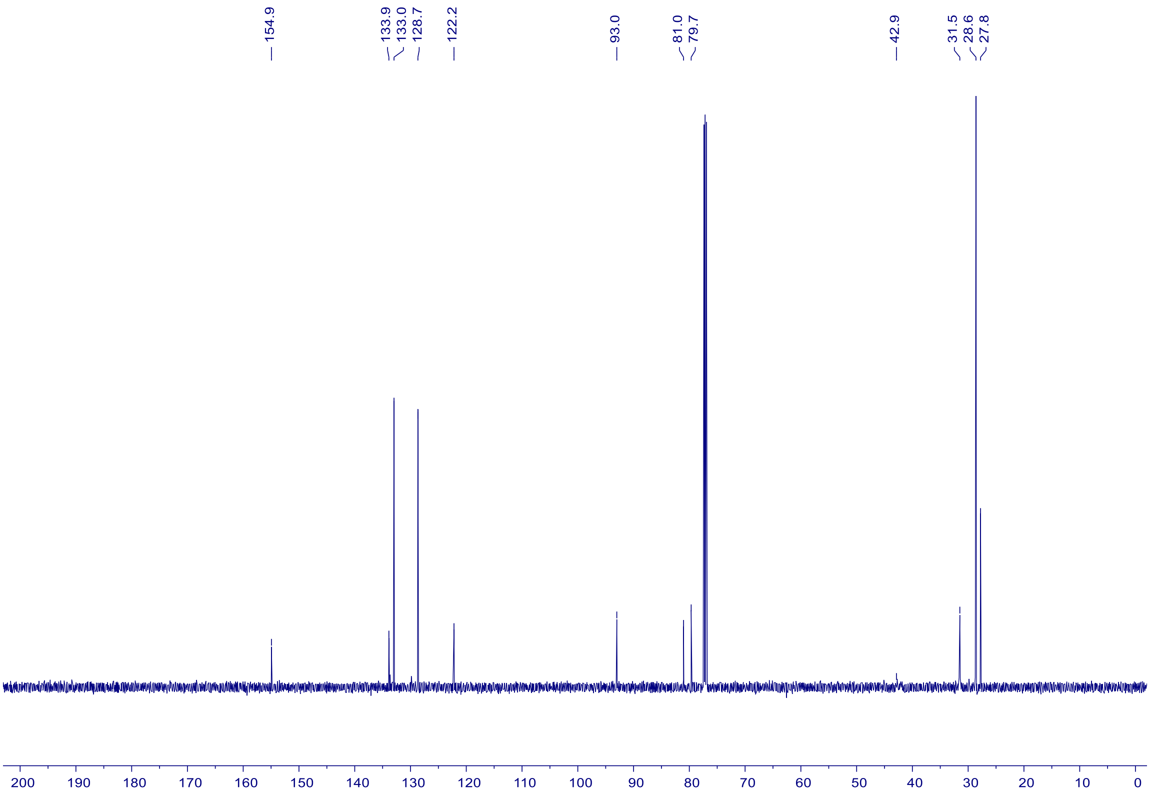

8 – ^{1}H NMR (600 MHz, CDCl$_3$)

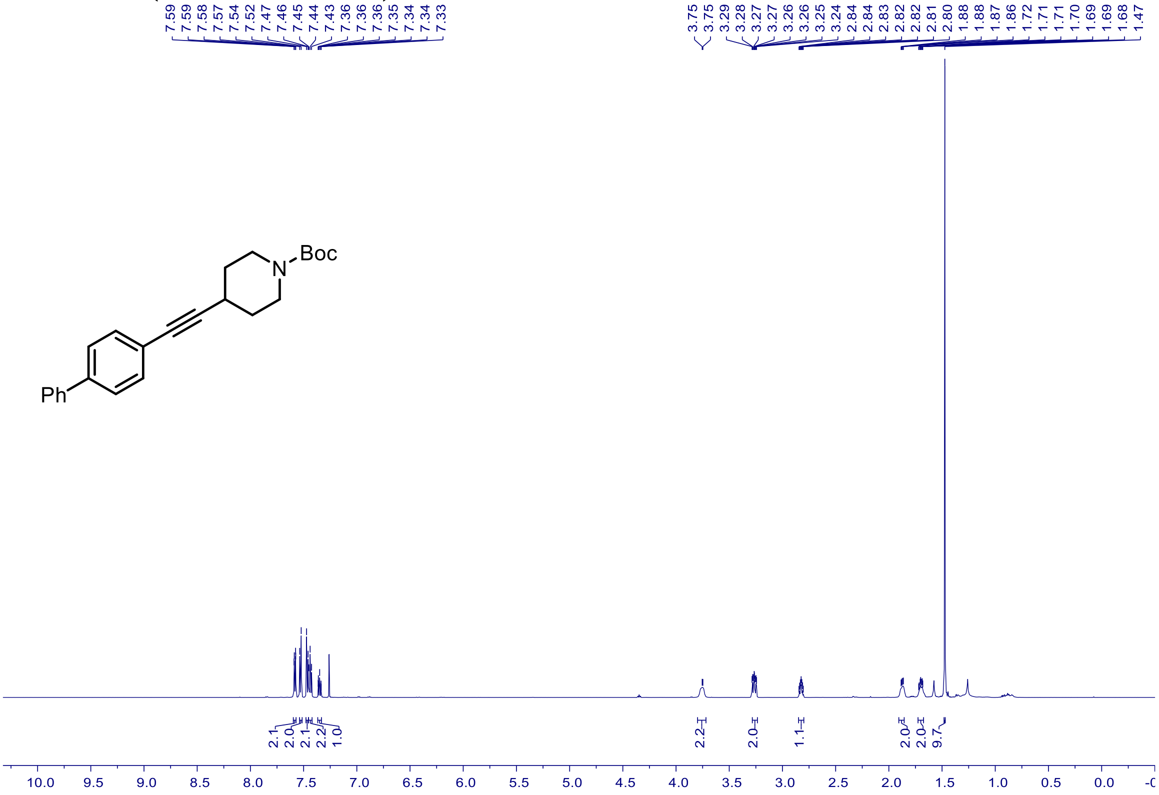

8 – ^{13}C NMR (151 MHz, CDCl$_3$)

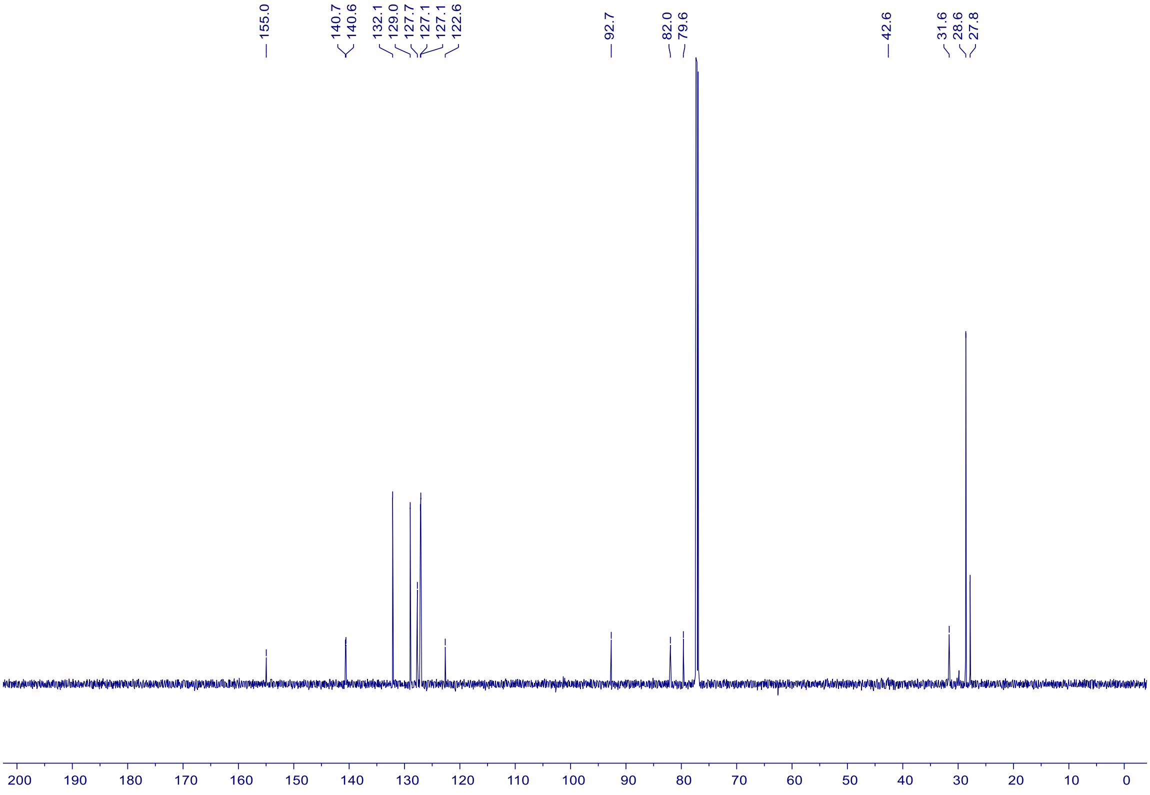

9 – ^{1}H NMR (600 MHz, $CDCl_3$)

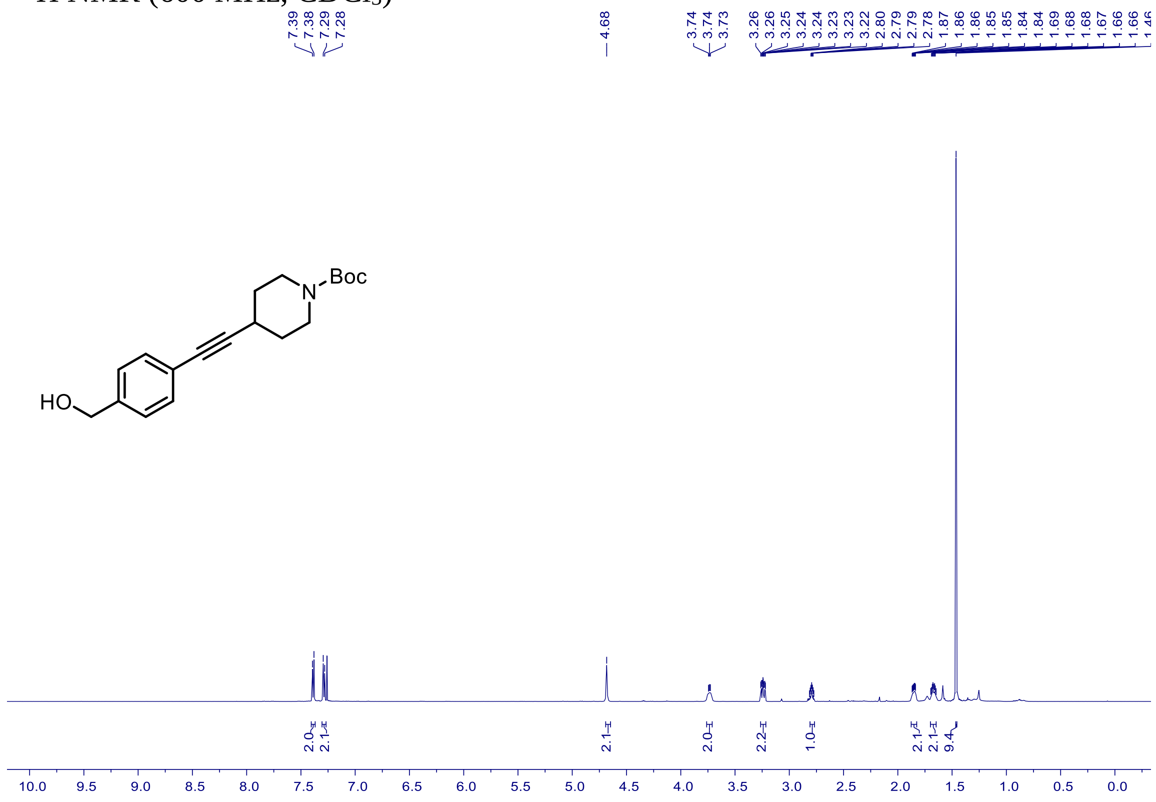

9 – ^{13}C NMR (151 MHz, $CDCl_3$)

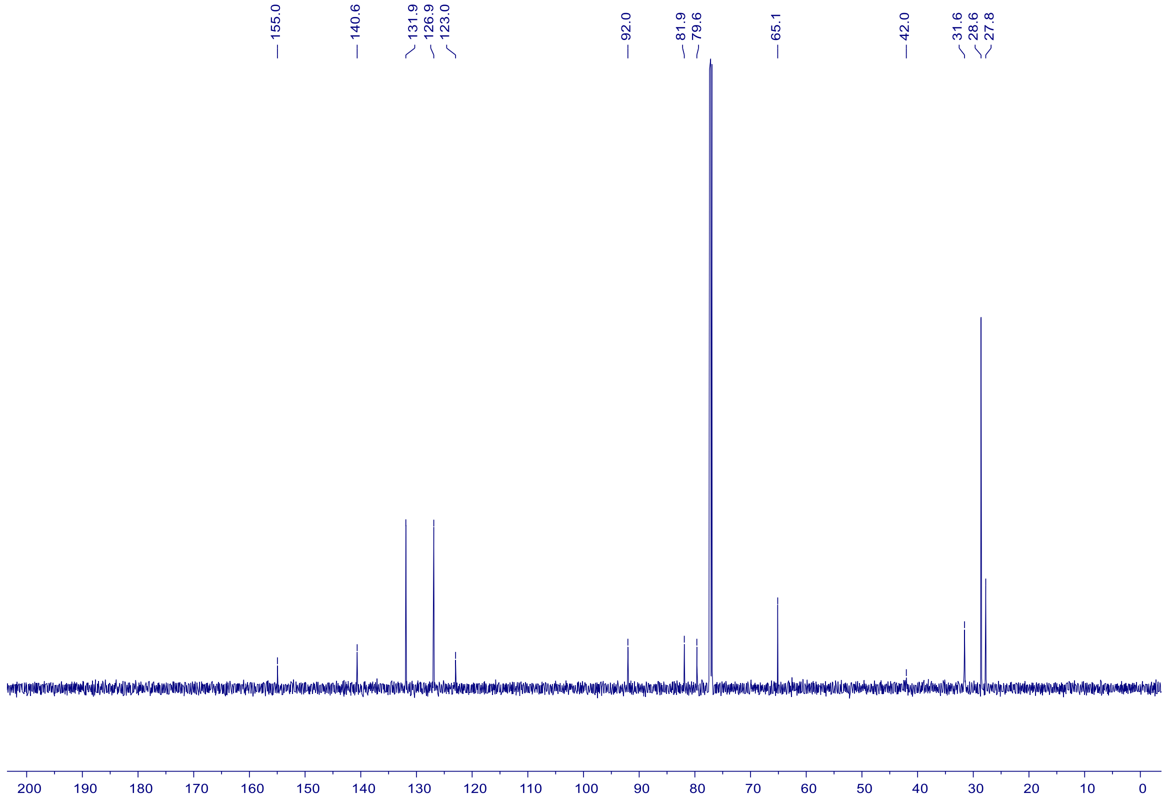

10 – 1H NMR (600 MHz, $CDCl_3$)

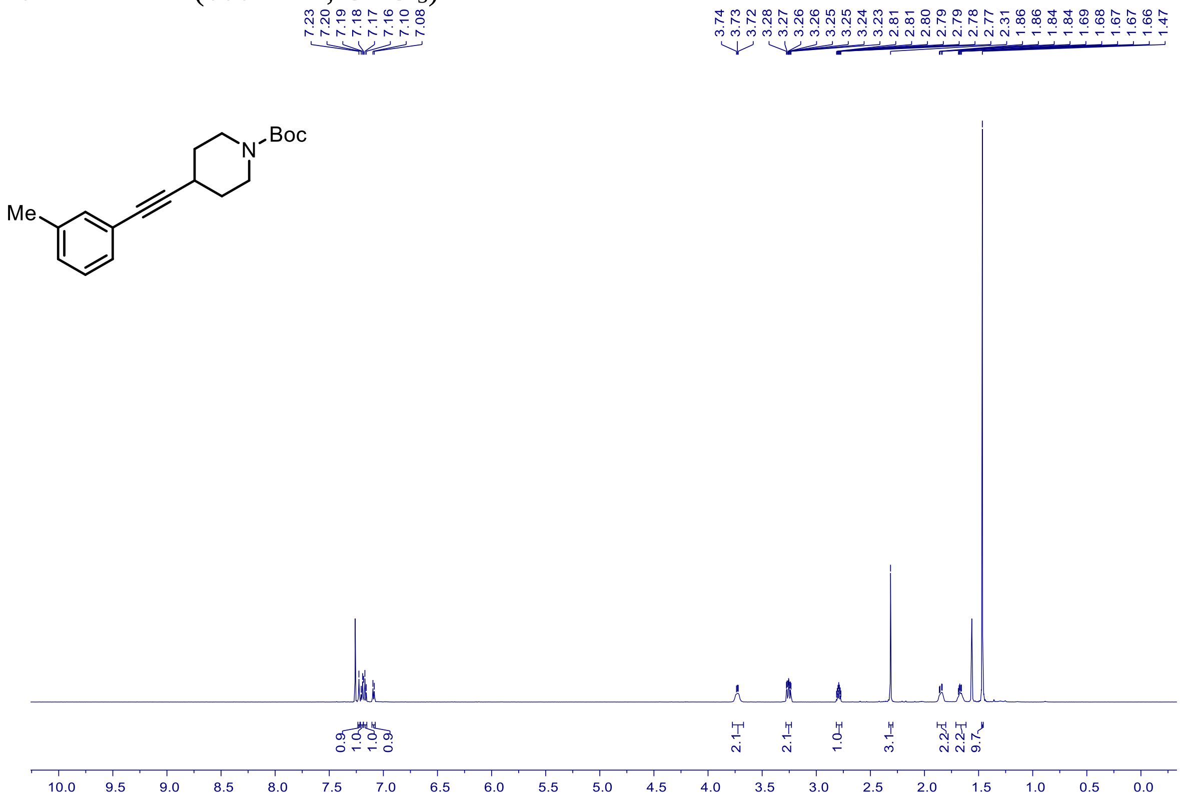

10 – ^{13}C NMR (151 MHz, $CDCl_3$)

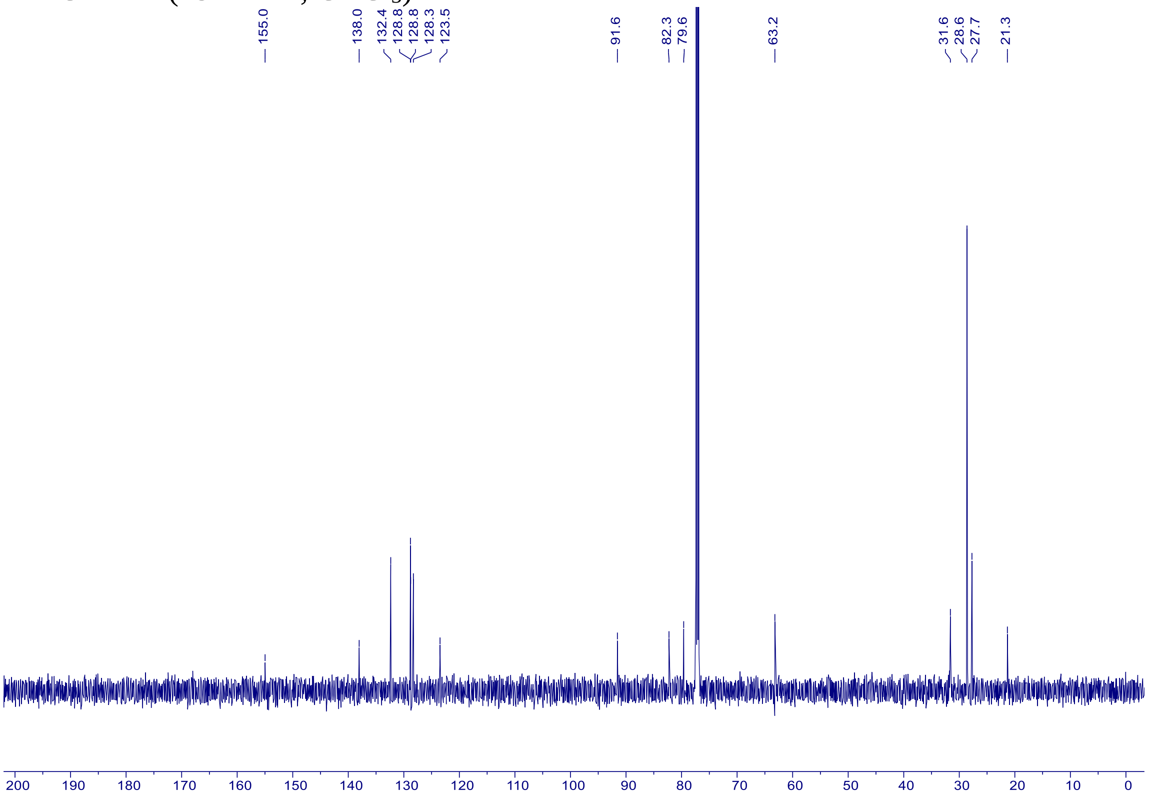

11 – ^{1}H NMR (600 MHz, $CDCl_3$)

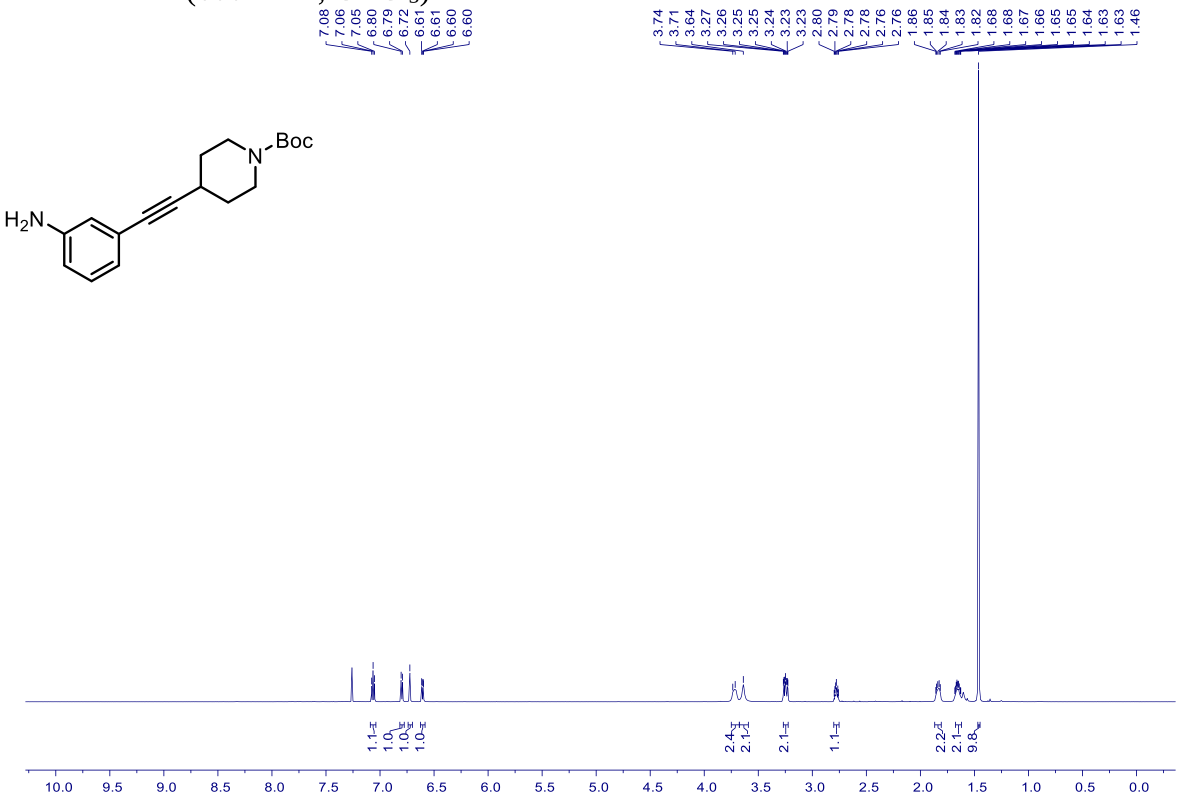

11 – ^{13}C NMR (151 MHz, $CDCl_3$)

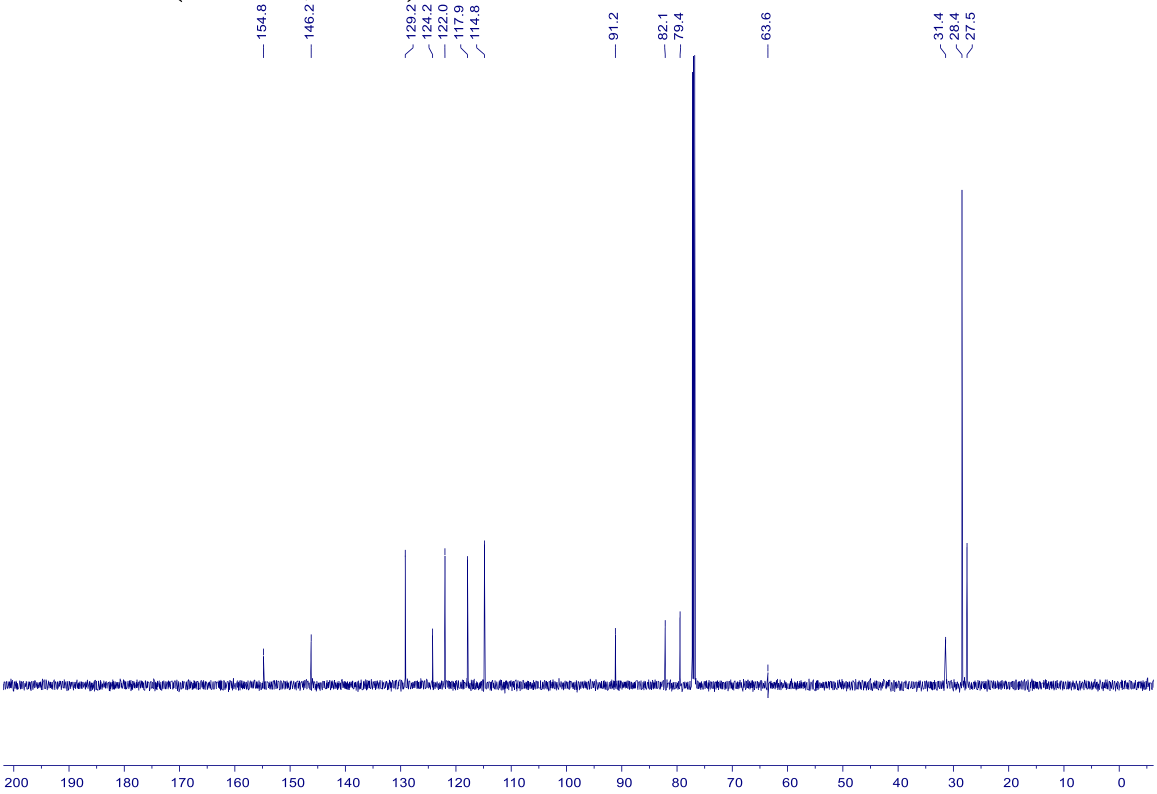

12 – ^{1}H NMR (600 MHz, $CDCl_3$)

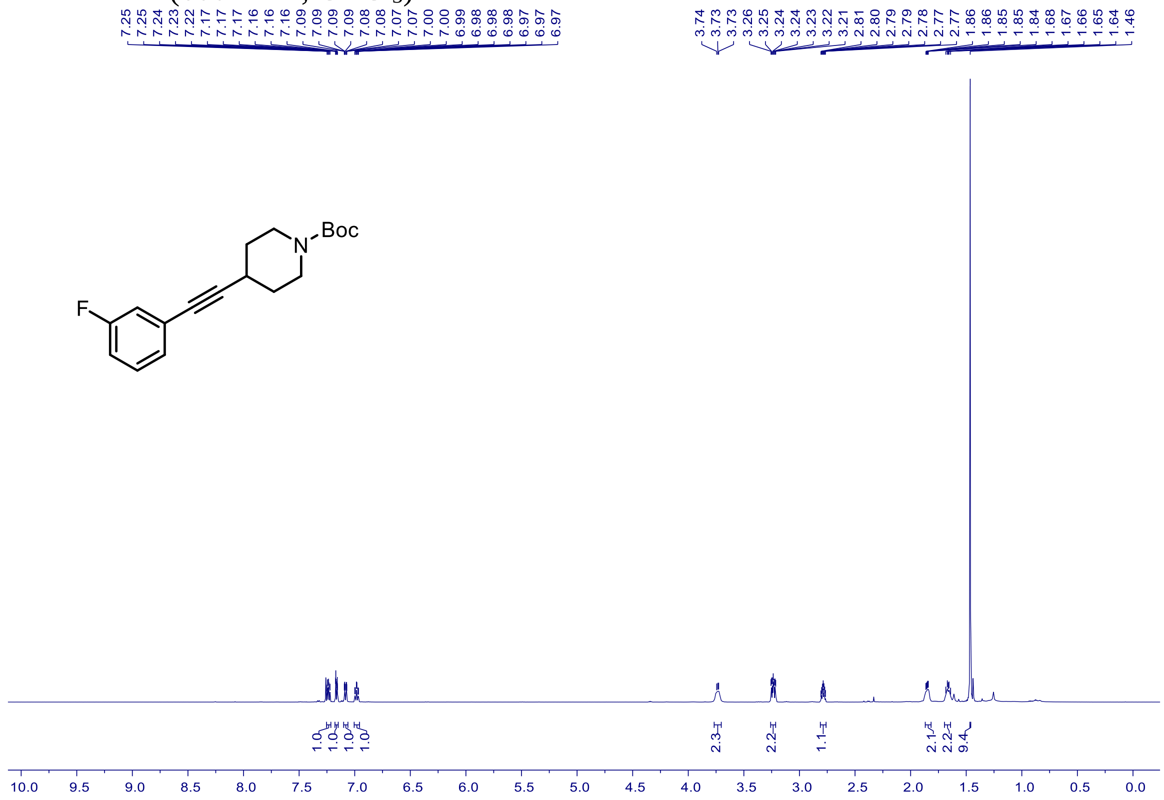

12 – ^{13}C NMR (151 MHz, $CDCl_3$)

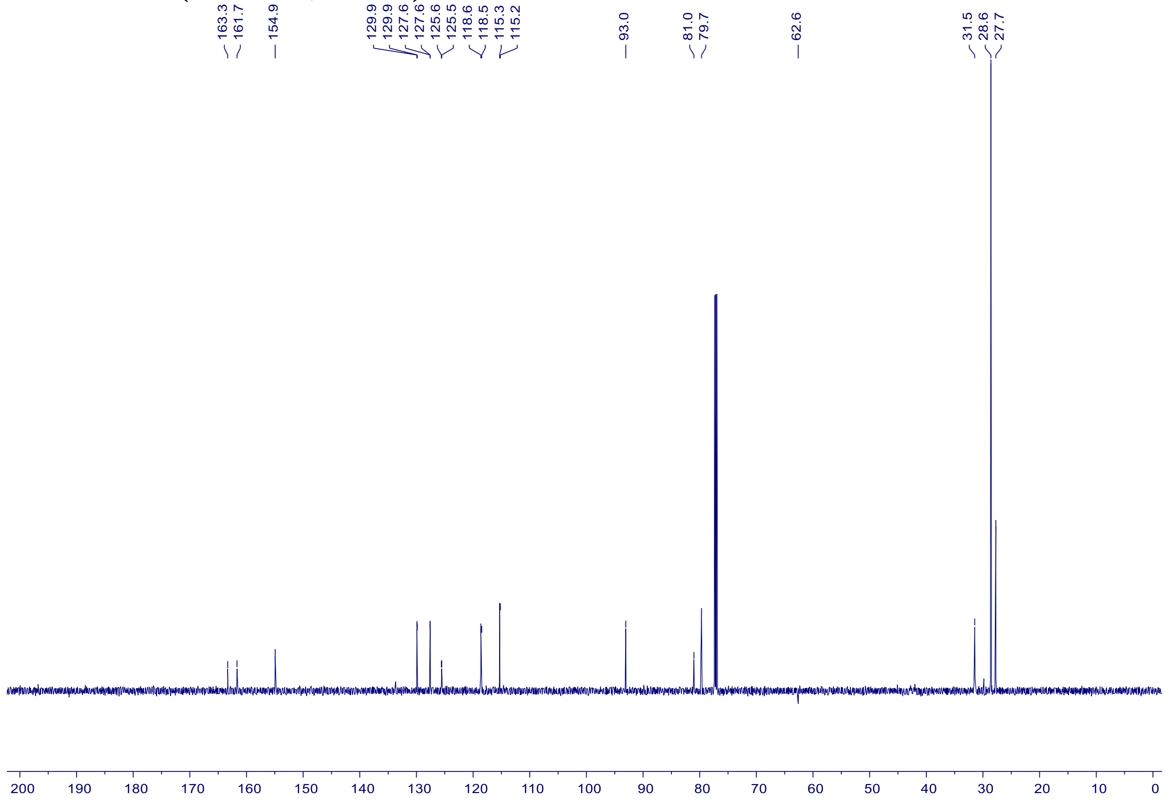

12 – ^{19}F NMR (565 MHz, $CDCl_3$)

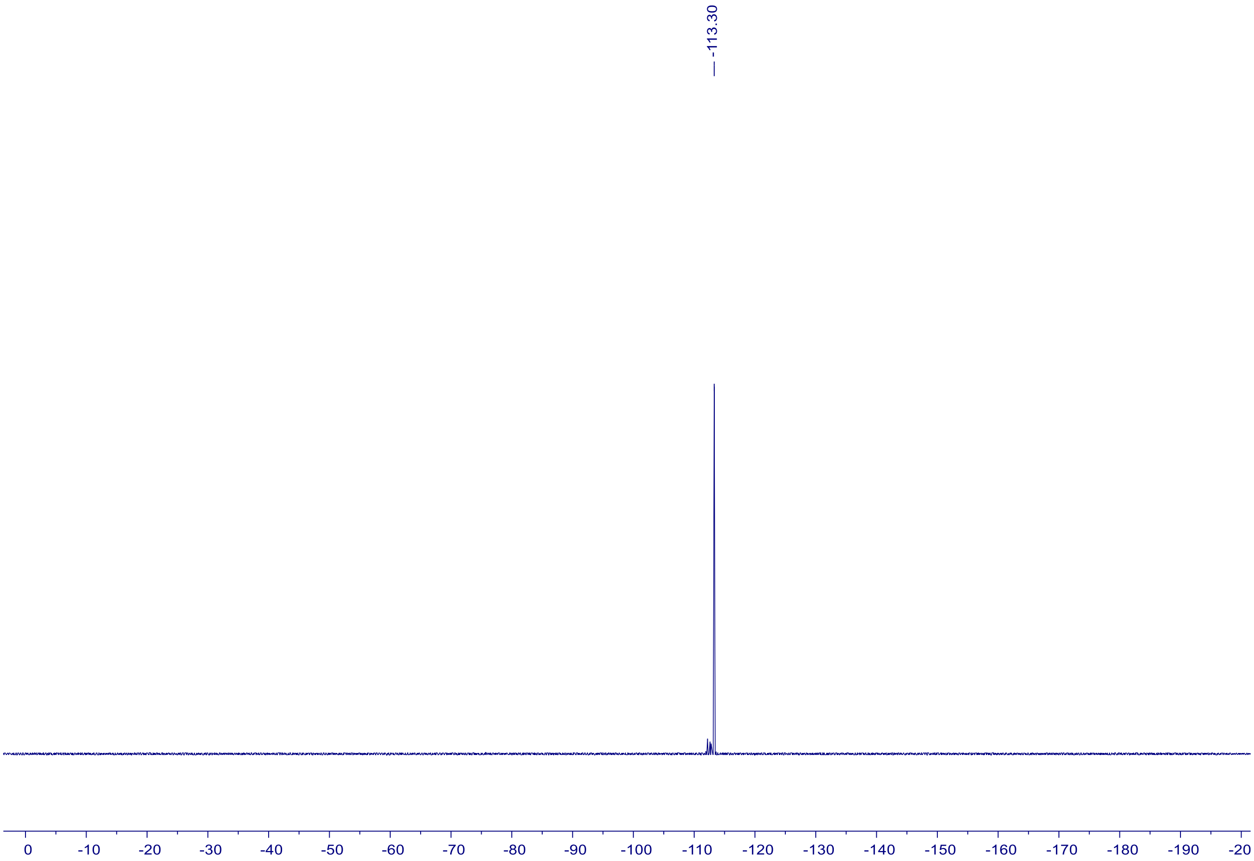

13 – ^{1}H NMR (600 MHz, $CDCl_3$)

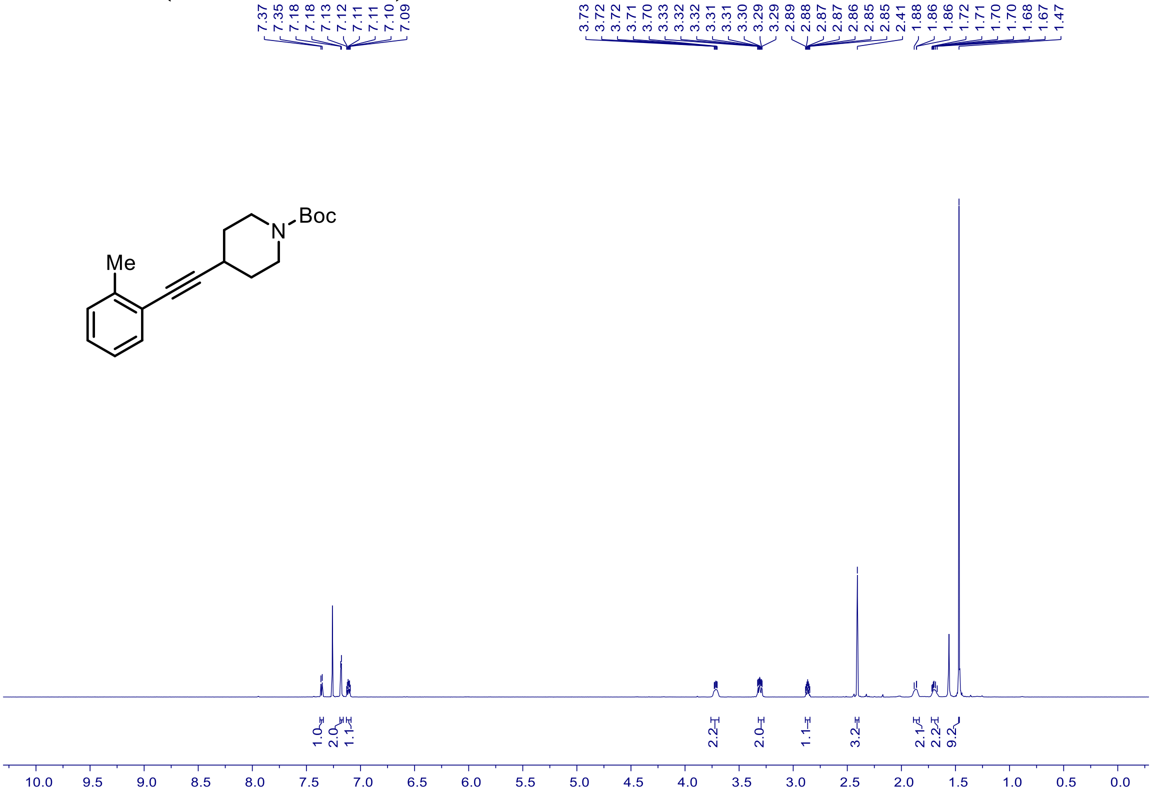

13 – ^{13}C NMR (151 MHz, $CDCl_3$)

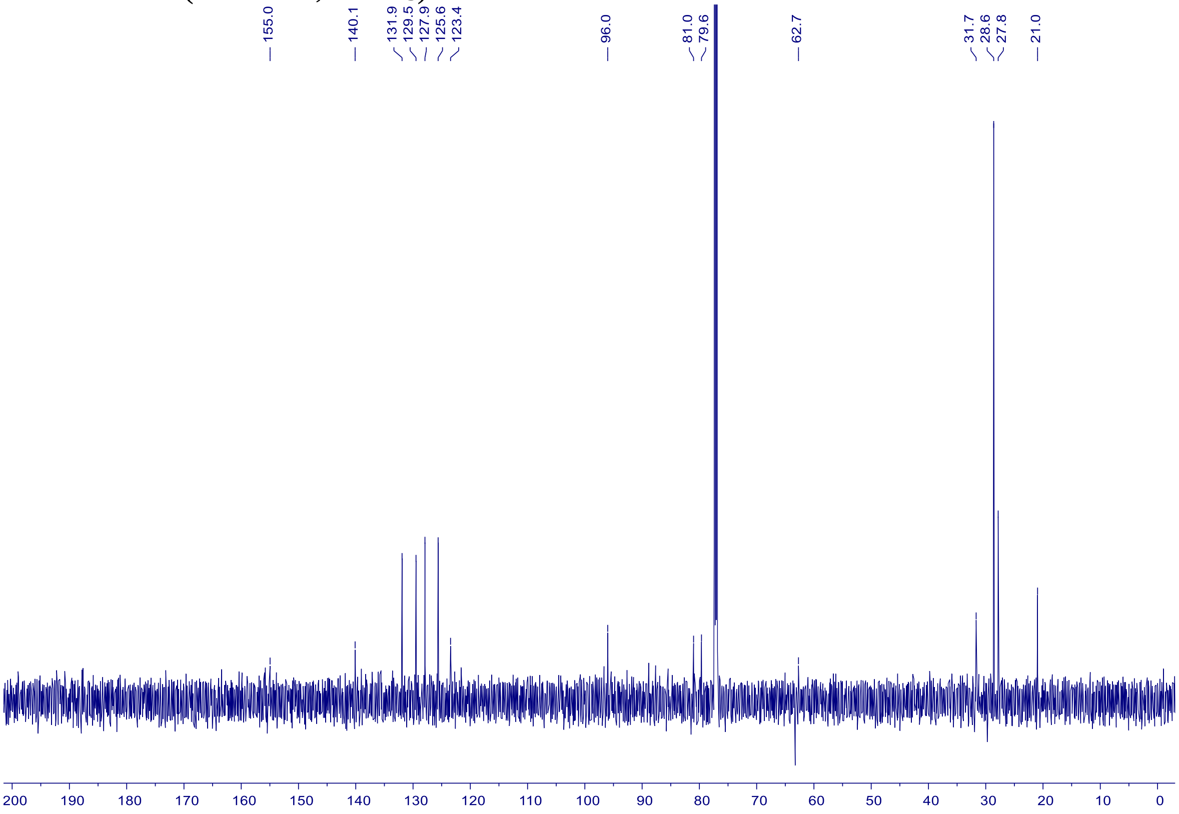

14 – ^{1}H NMR (600 MHz, $CDCl_3$)

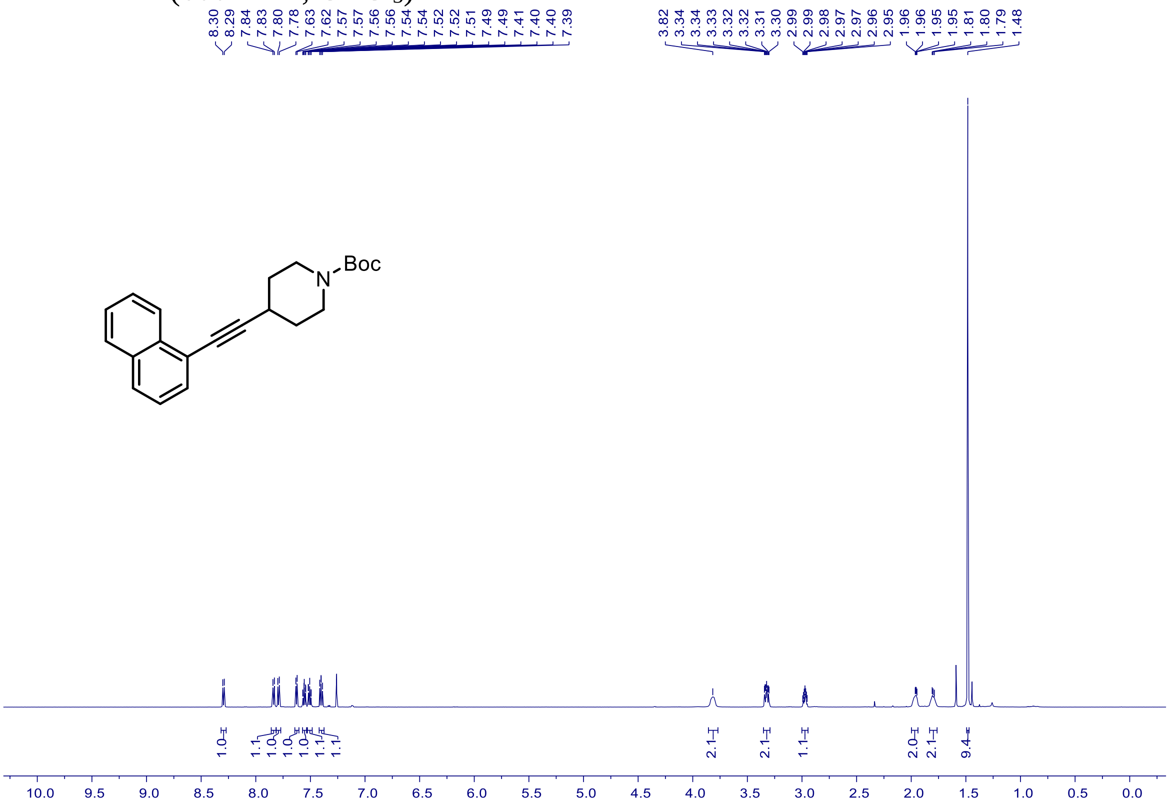

14 – ^{13}C NMR (151 MHz, $CDCl_3$)

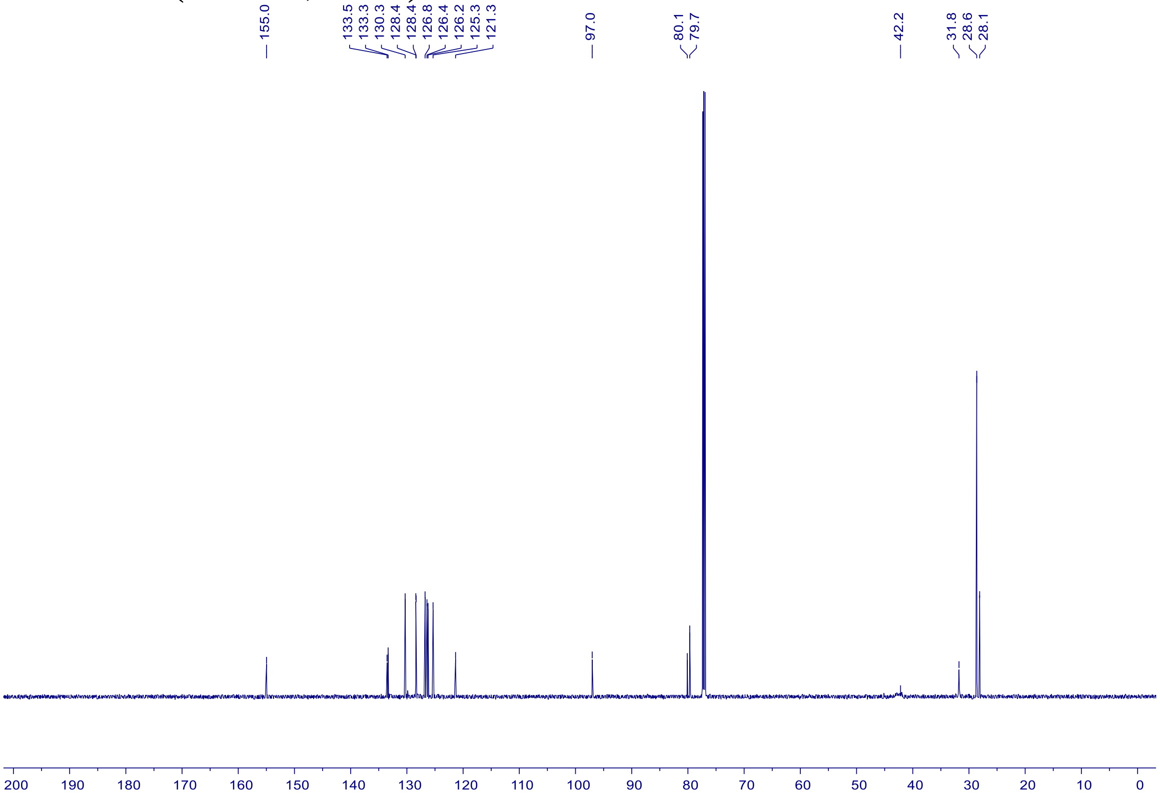

15 – ^{1}H NMR (600 MHz, $CDCl_3$)

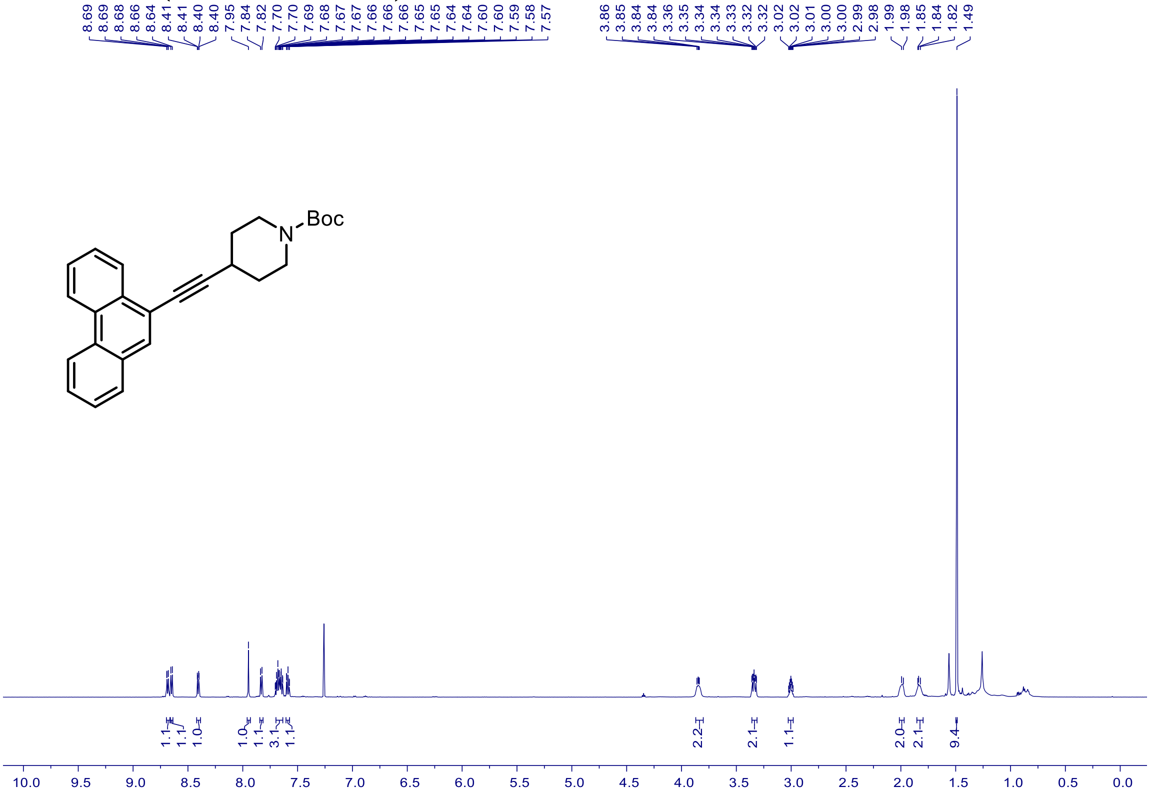

15 – ^{13}C NMR (151 MHz, $CDCl_3$)

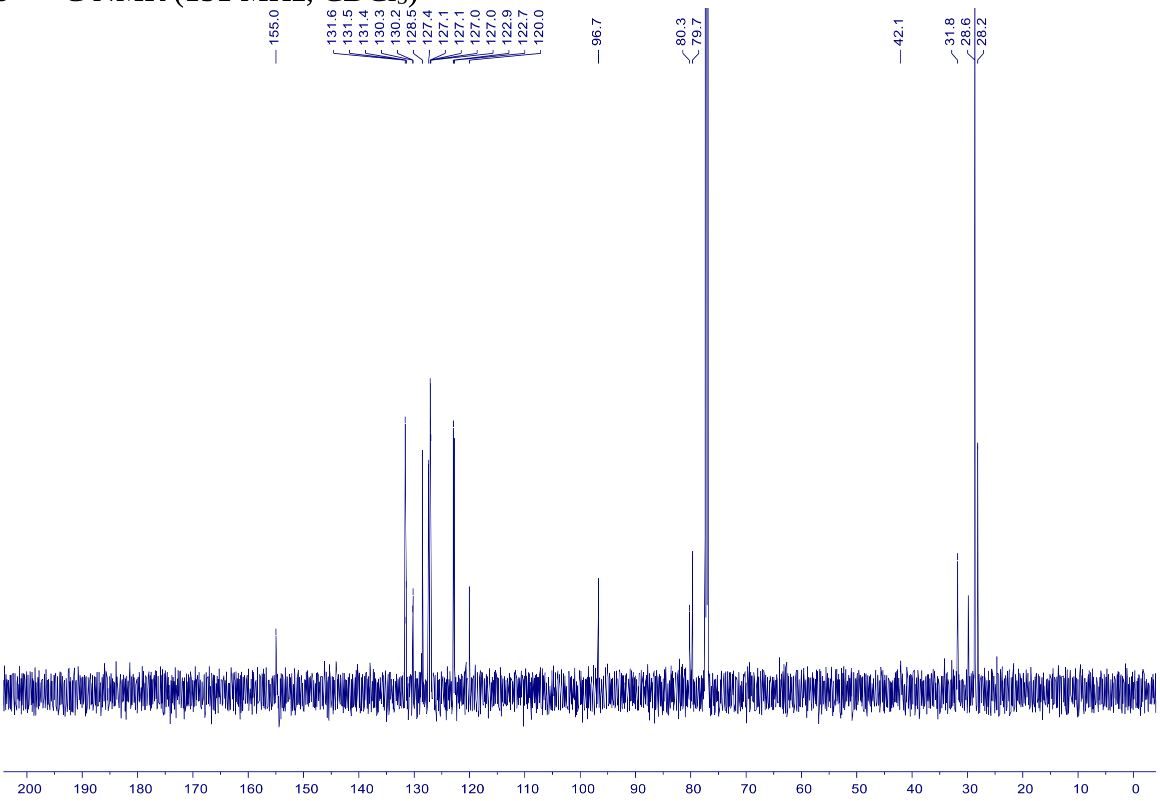

16 – ^{1}H NMR (600 MHz, $CDCl_3$)

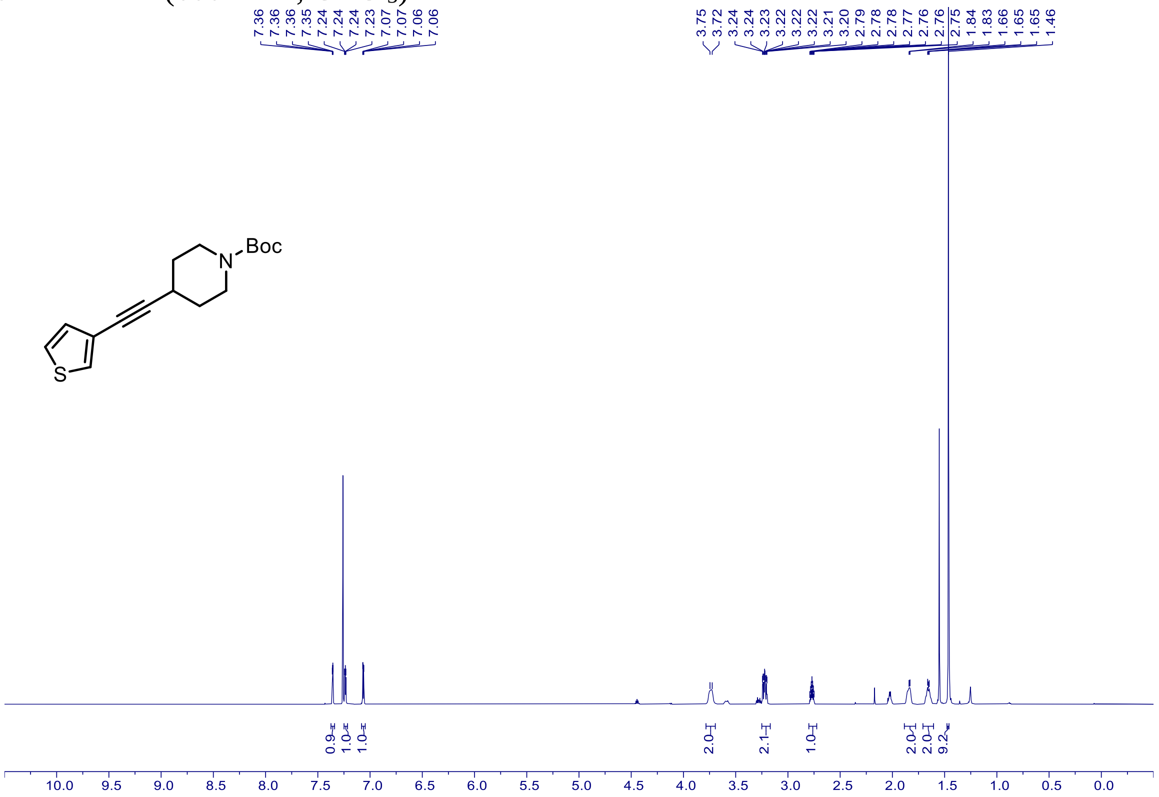

16 – ^{13}C NMR (151 MHz, $CDCl_3$)

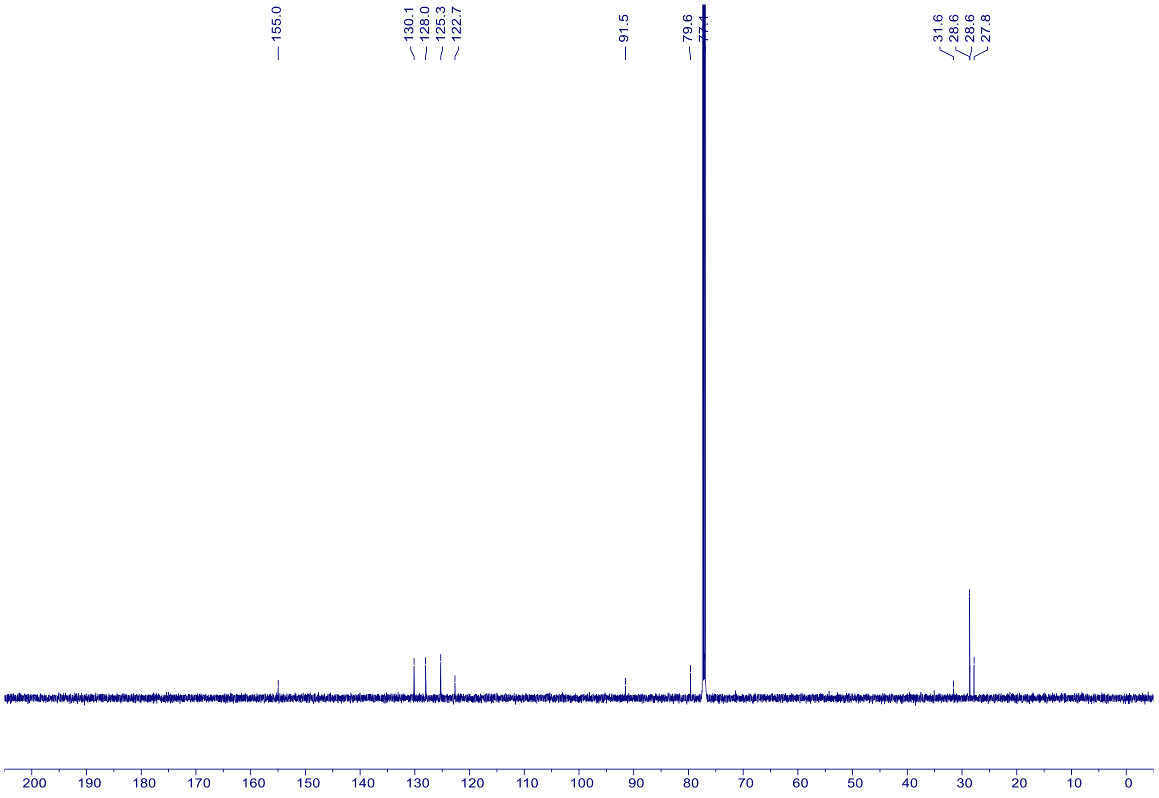

17 – ^{1}H NMR (600 MHz, $CDCl_3$)

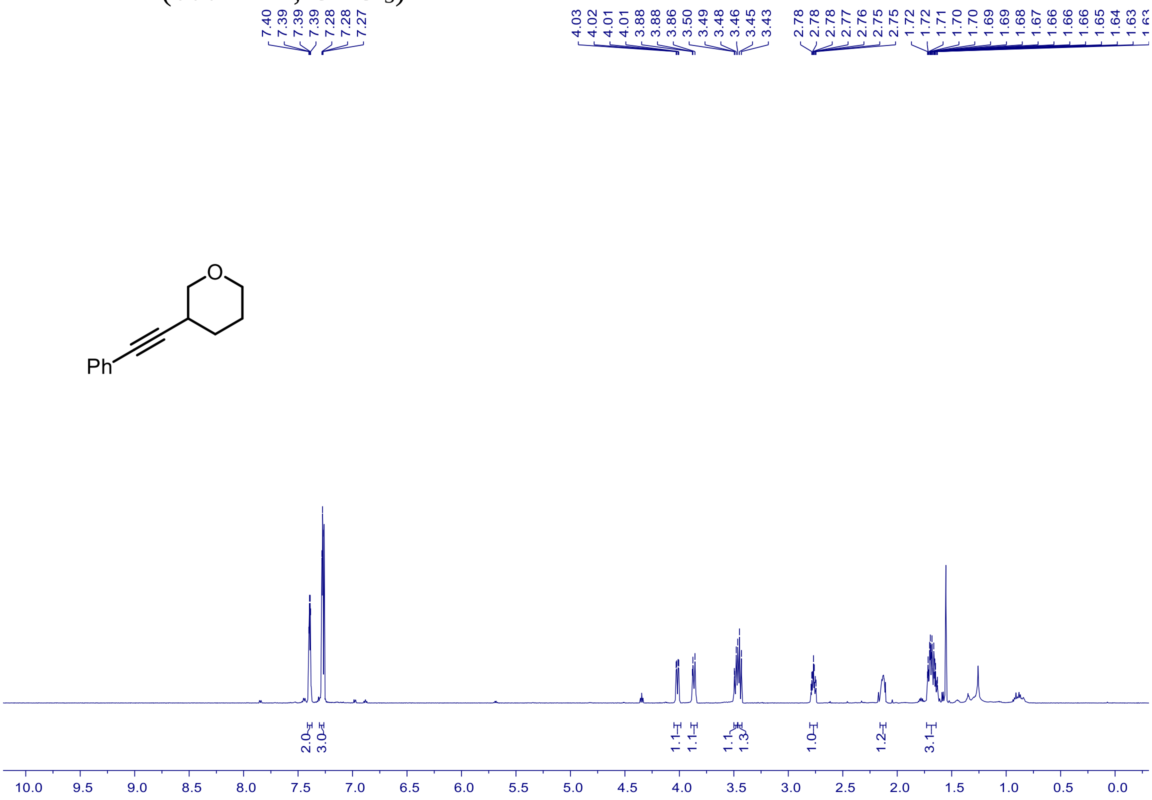

17 – ^{13}C NMR (151 MHz, $CDCl_3$)

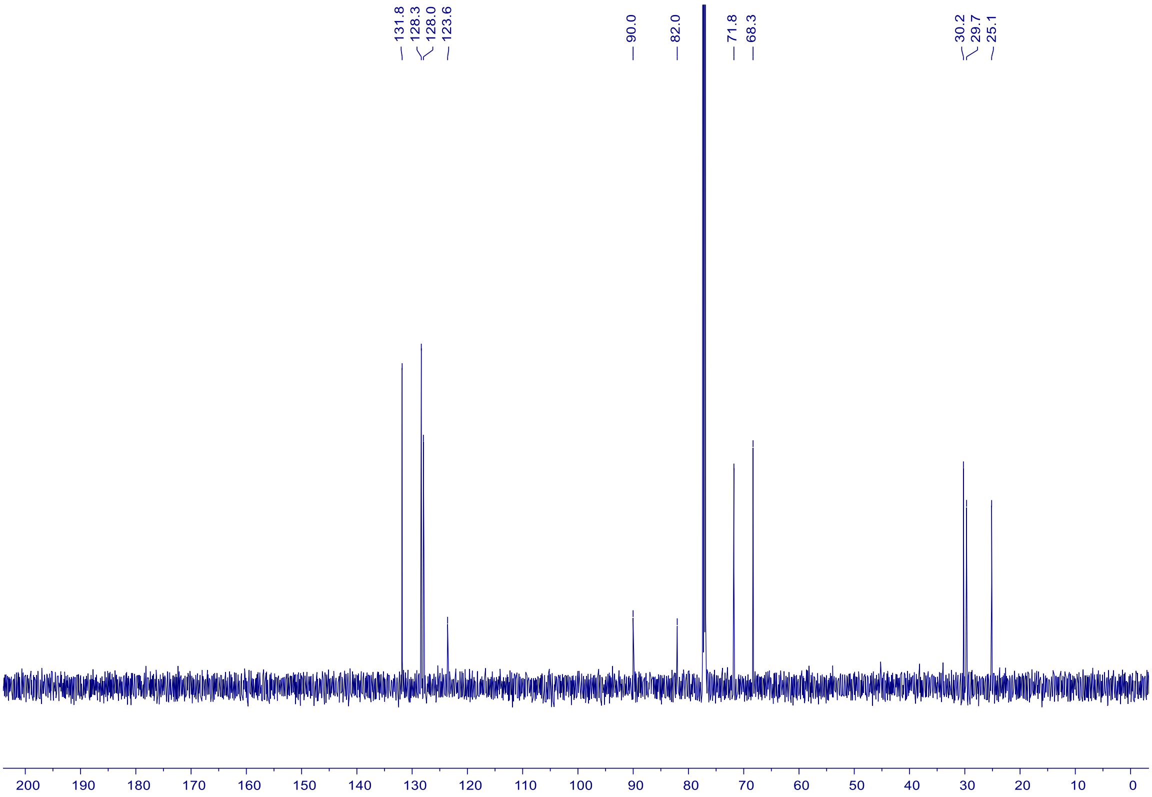

19 – ^{1}H NMR (600 MHz, $CDCl_3$)

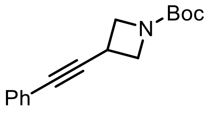

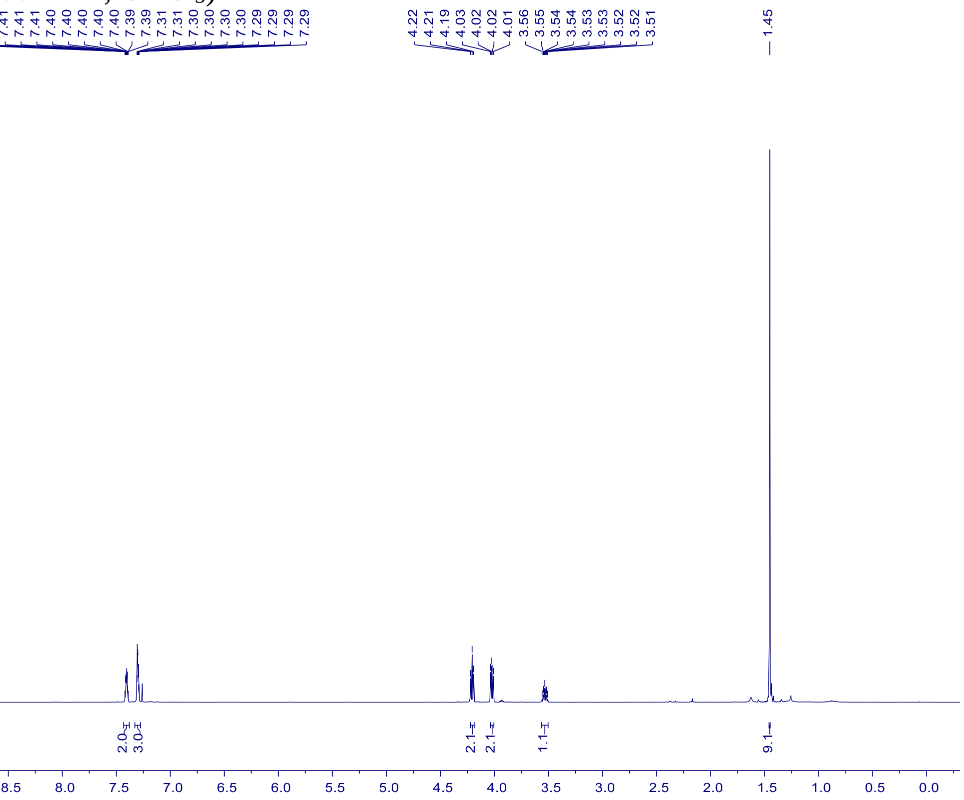

19 – ^{13}C NMR (151 MHz, $CDCl_3$)

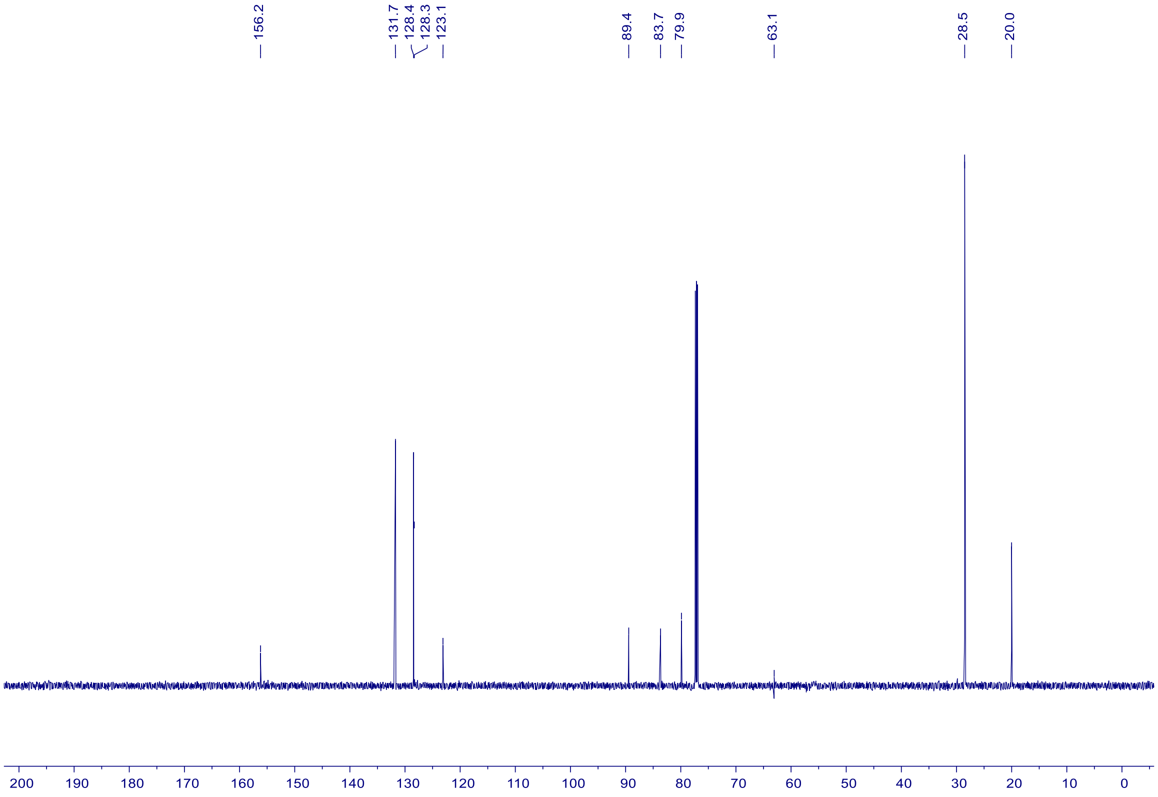

21 – ^{1}H NMR (600 MHz, $CDCl_3$)

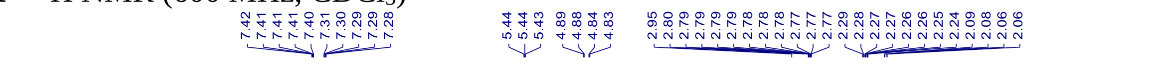

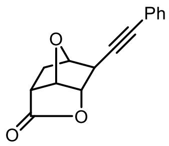

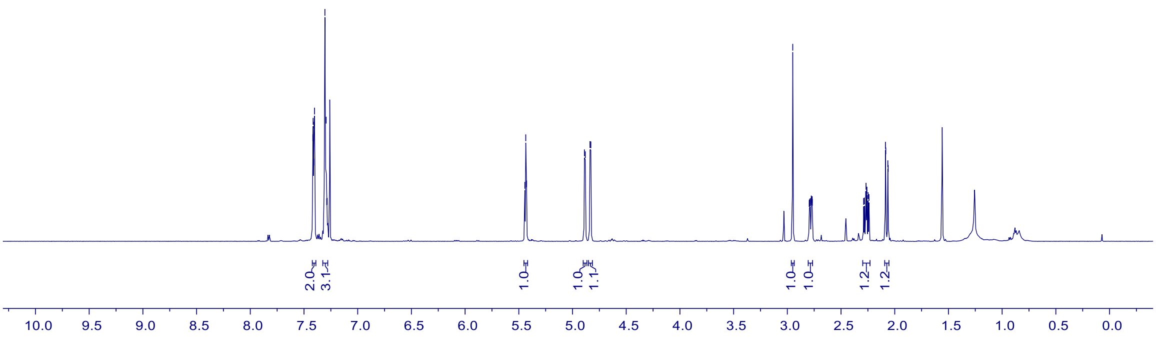

21 – ^{13}C NMR (151 MHz, $CDCl_3$)

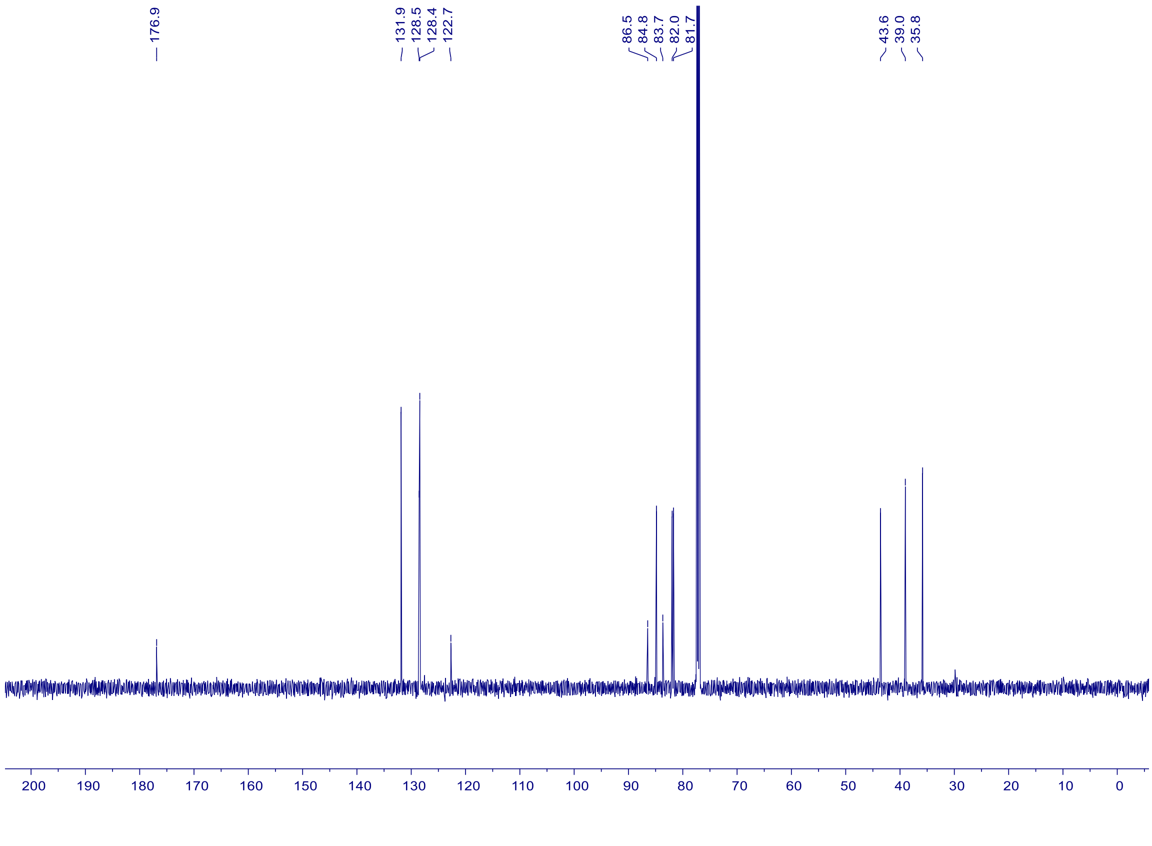

22 – ^{1}H NMR (600 MHz, $CDCl_3$)

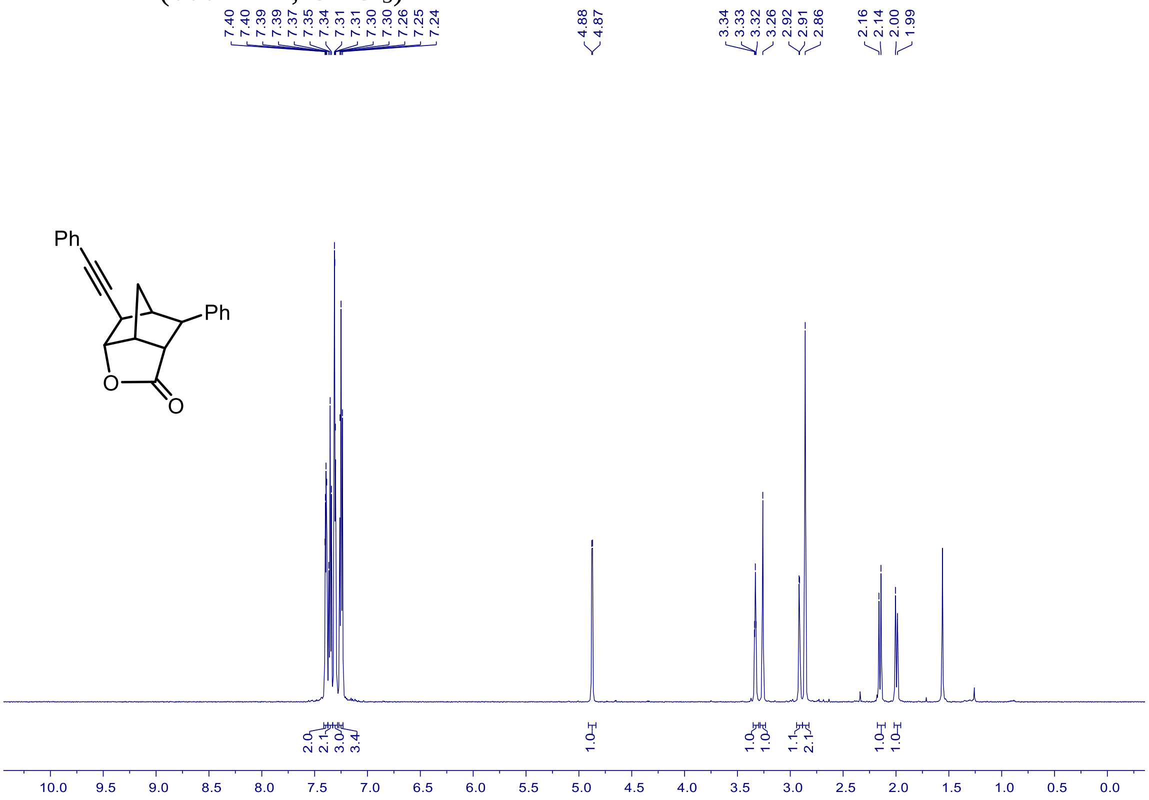

22 – ^{13}C NMR (151 MHz, $CDCl_3$)

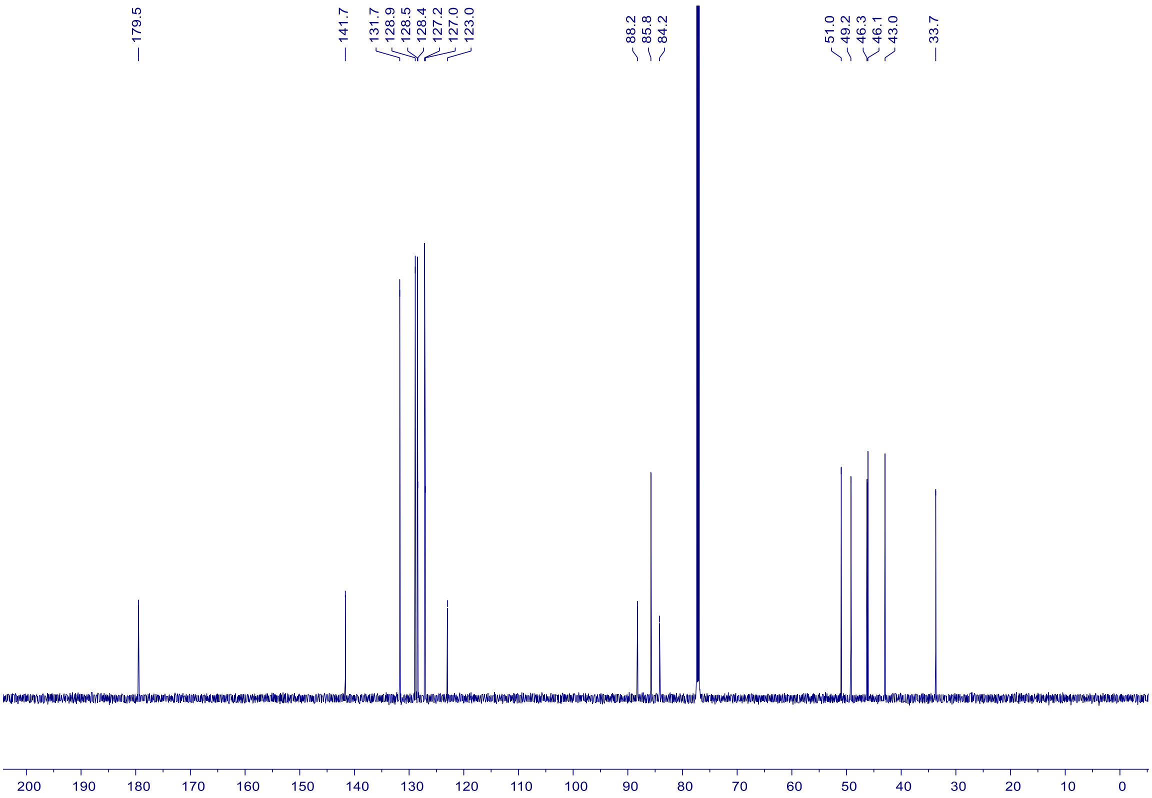

23 – ^{1}H NMR (600 MHz, $CDCl_3$)

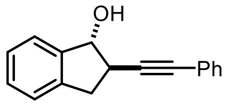

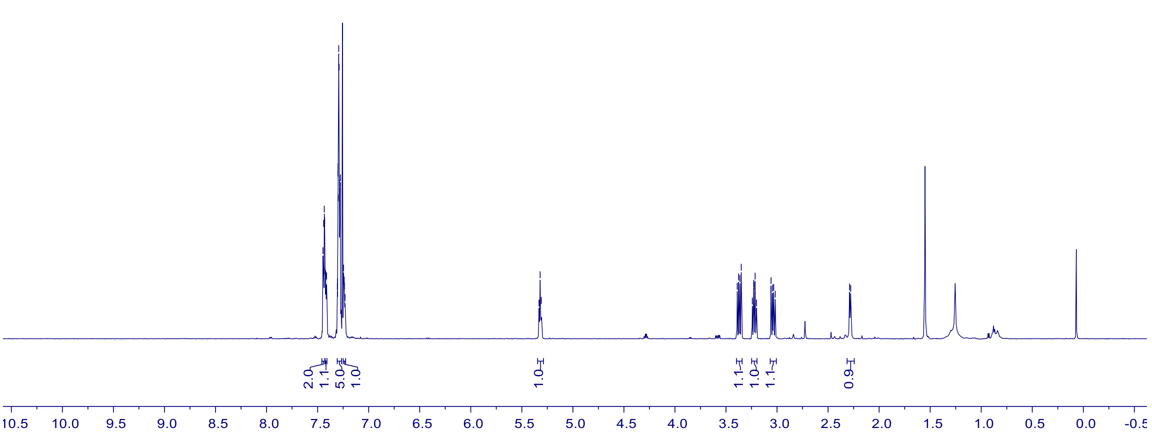

23 – ^{13}C NMR (151 MHz, $CDCl_3$)

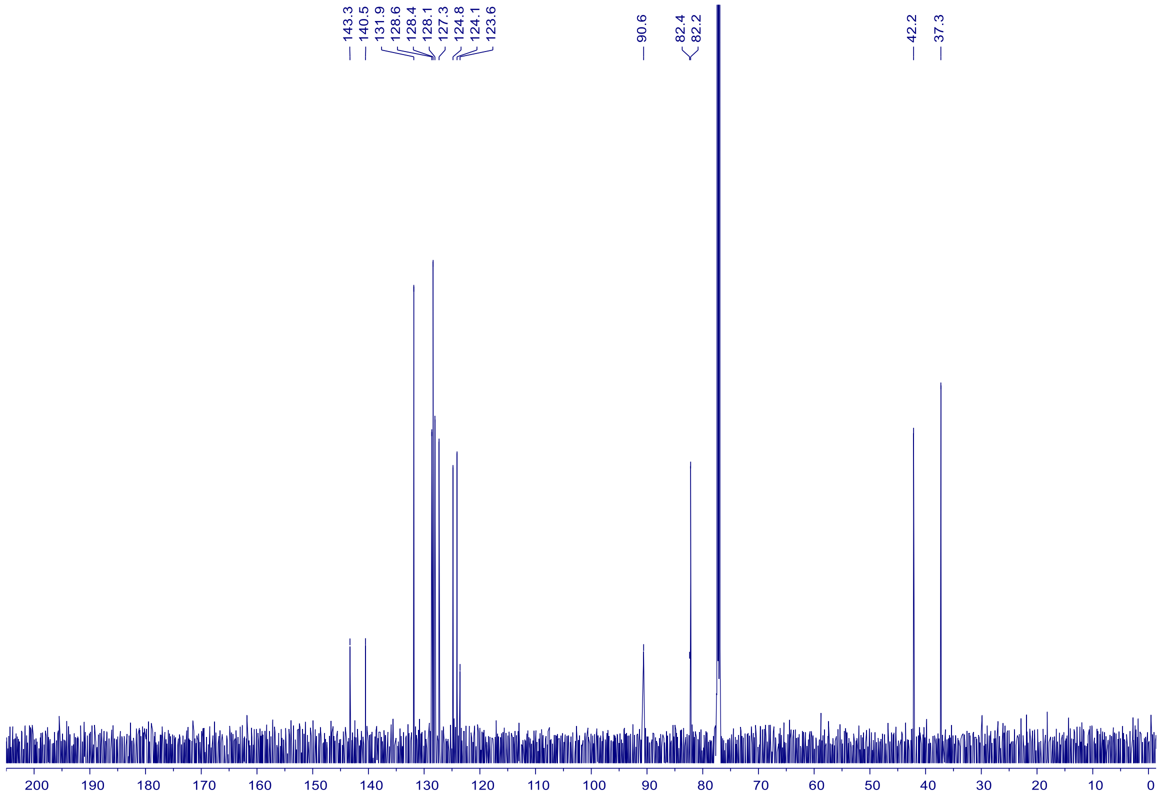

23 – Quantitative NOE (600 MHz, $CDCl_3$)

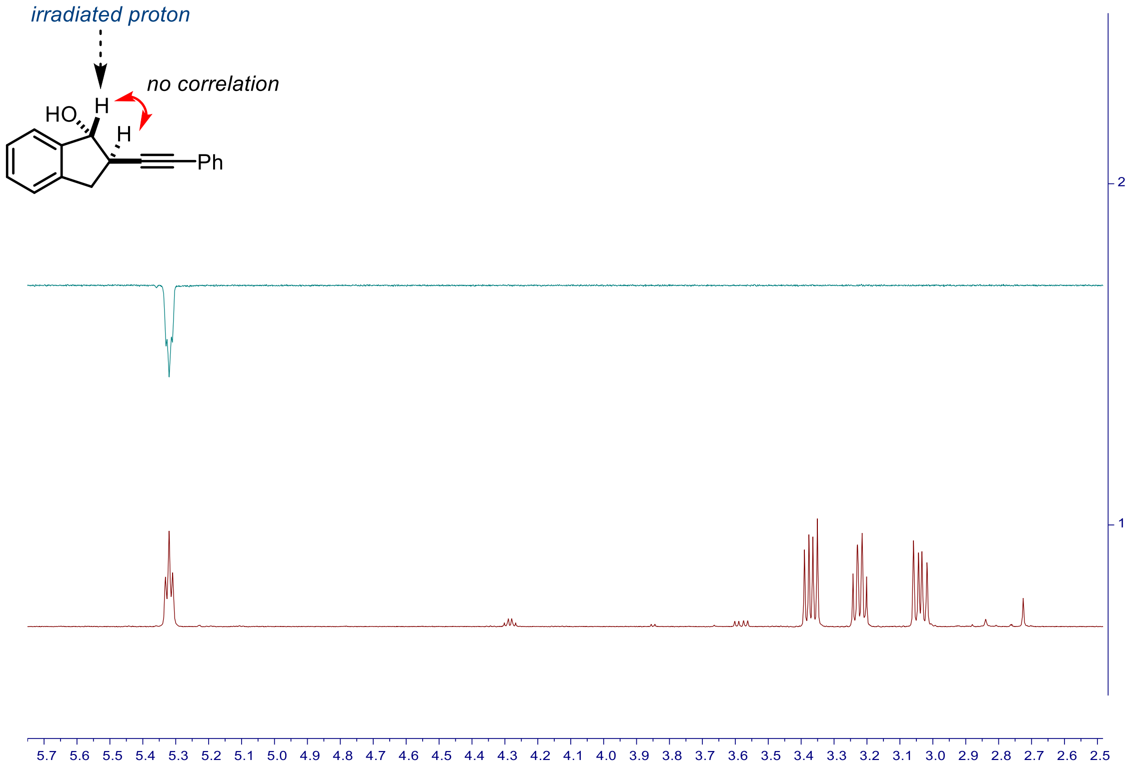

23 – Quantitative NOE (600 MHz, $CDCl_3$)

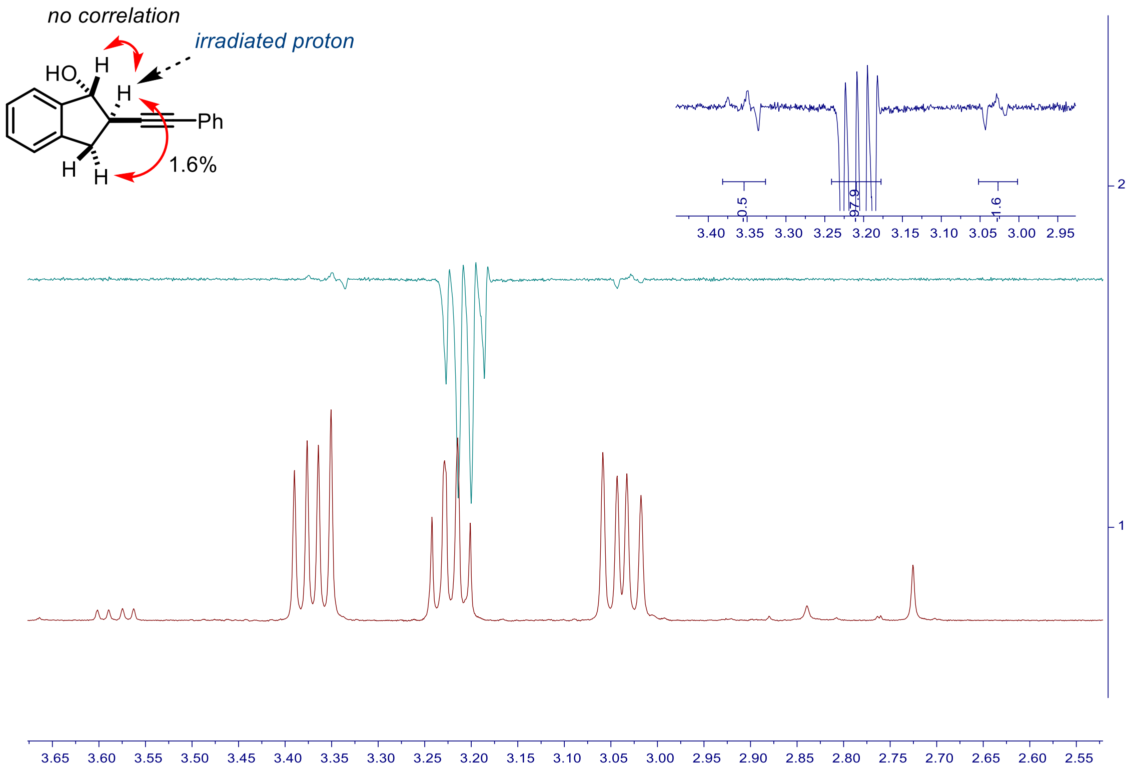

24 – ^{1}H NMR (600 MHz, $CDCl_3$)

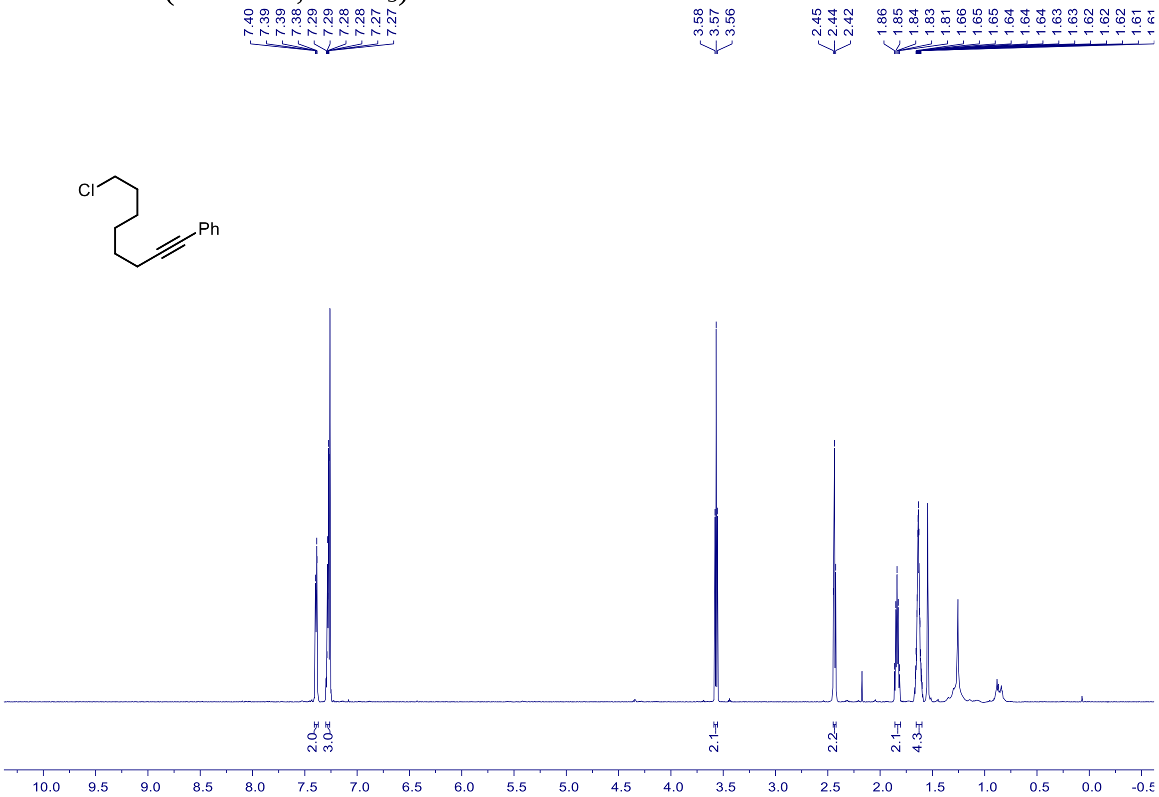

24 – ^{13}C NMR (151 MHz, $CDCl_3$)

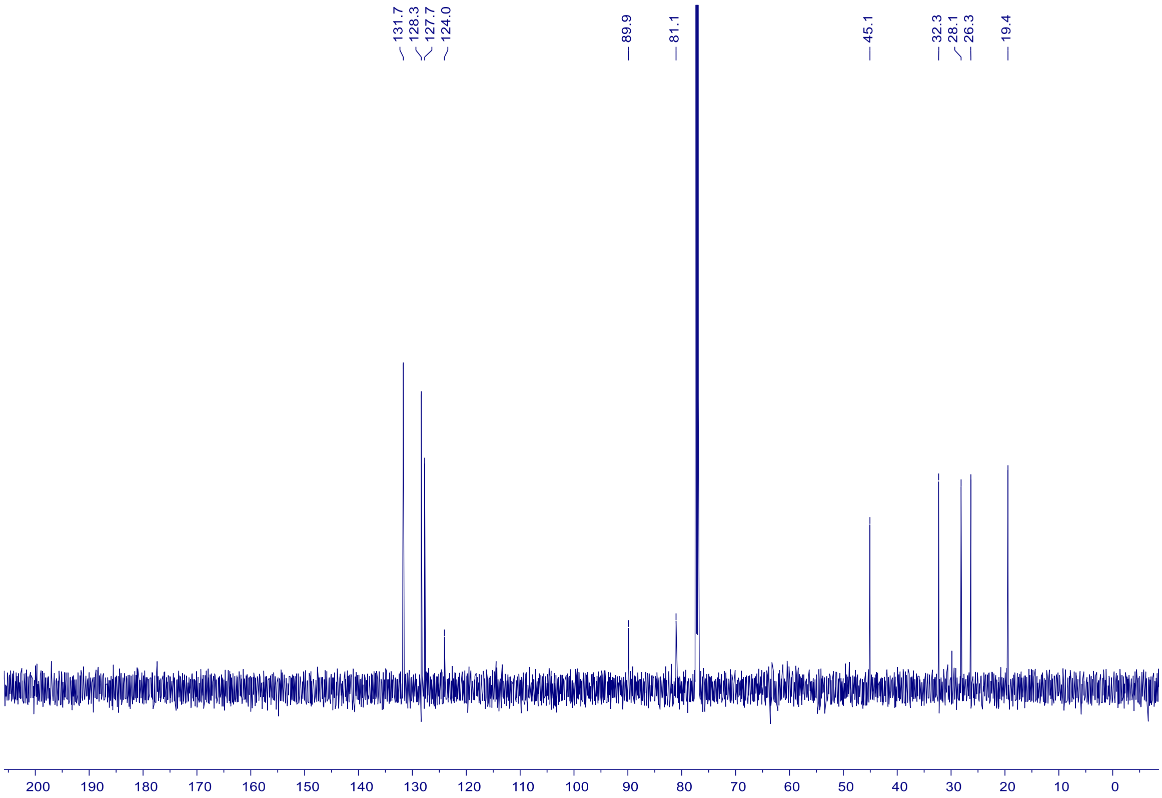

28 – ^{1}H NMR (400 MHz, $CDCl_3$)

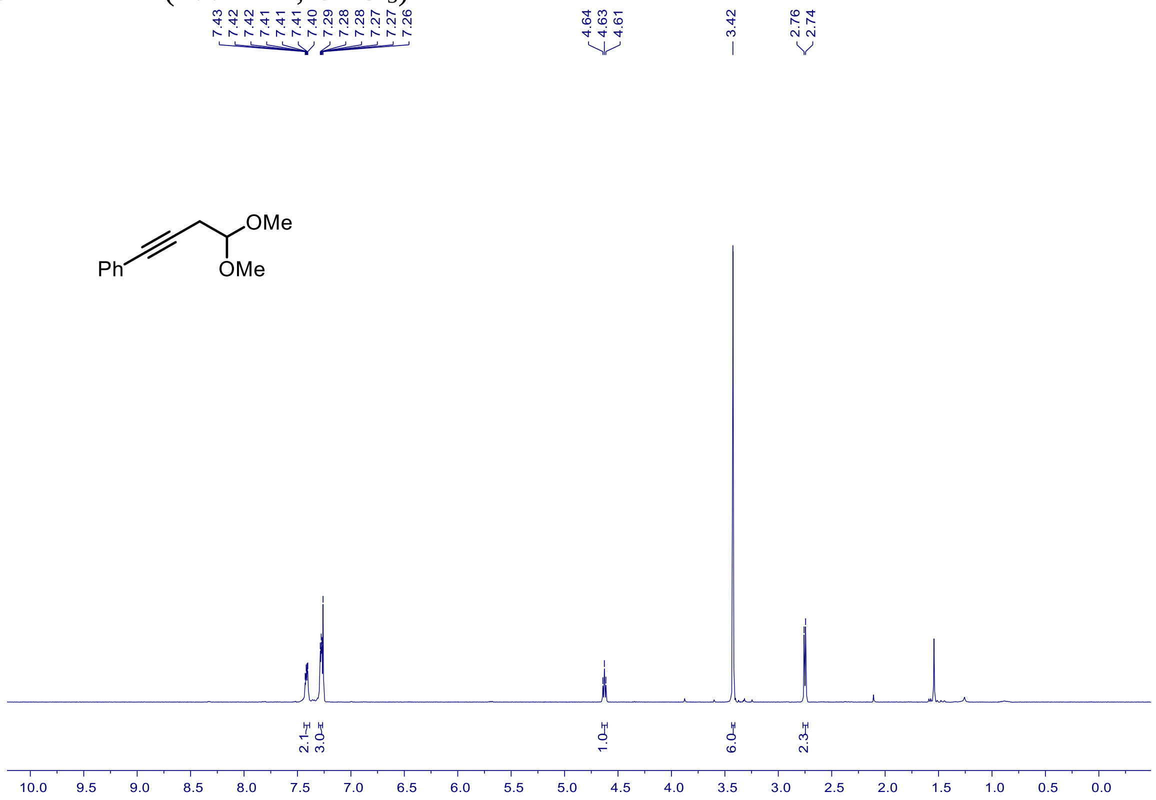

28 – ^{13}C NMR (101 MHz, $CDCl_3$)

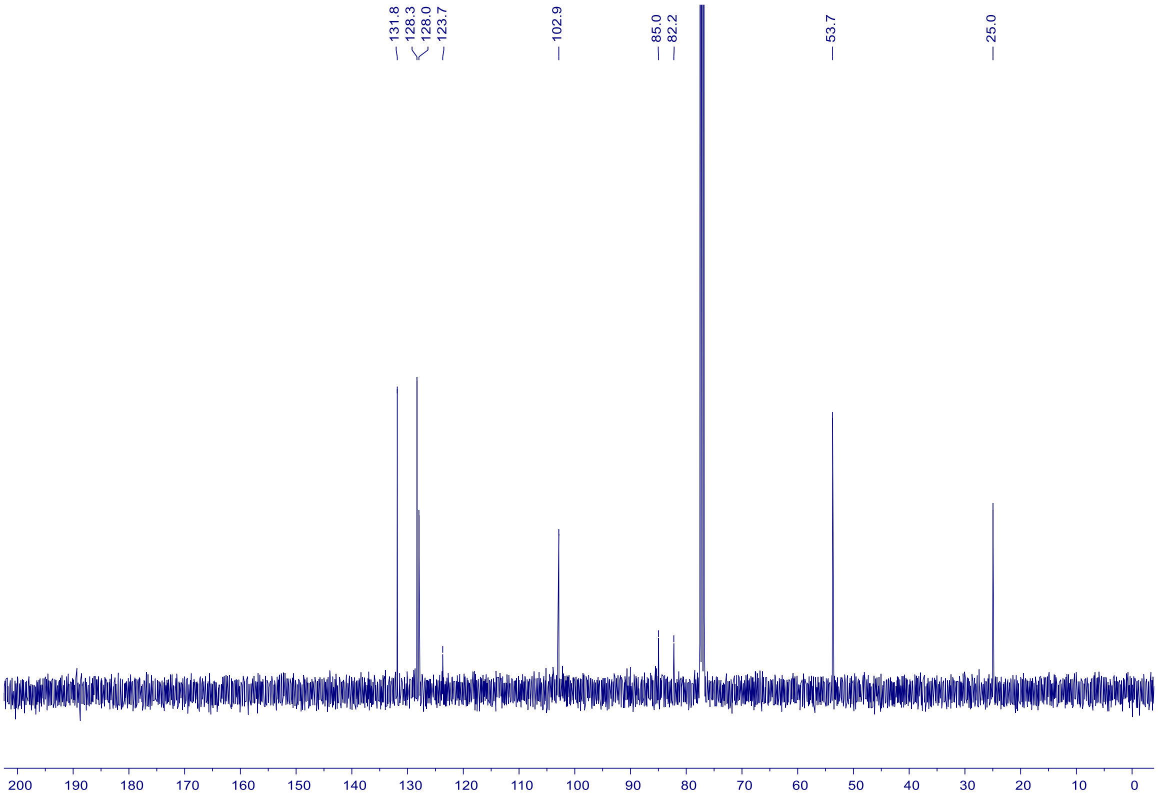

29 – ^{1}H NMR (600 MHz, $CDCl_3$)

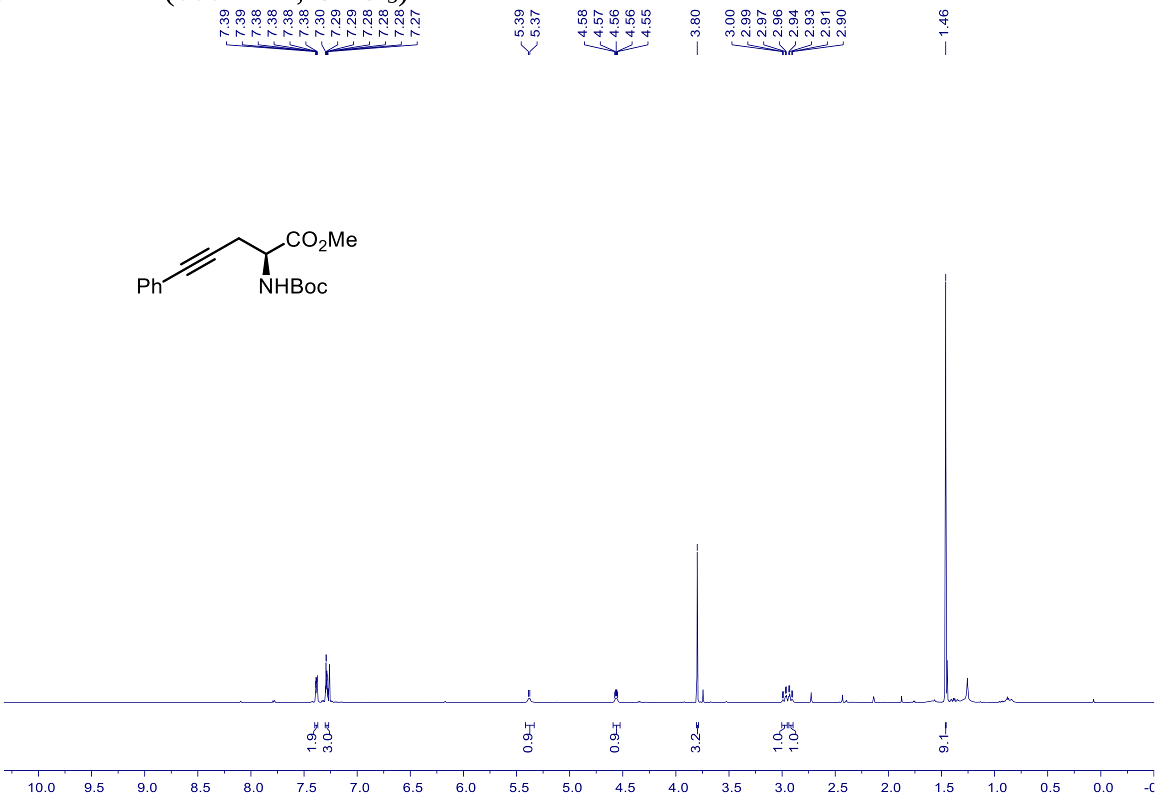

29 – ^{13}C NMR (151 MHz, $CDCl_3$)

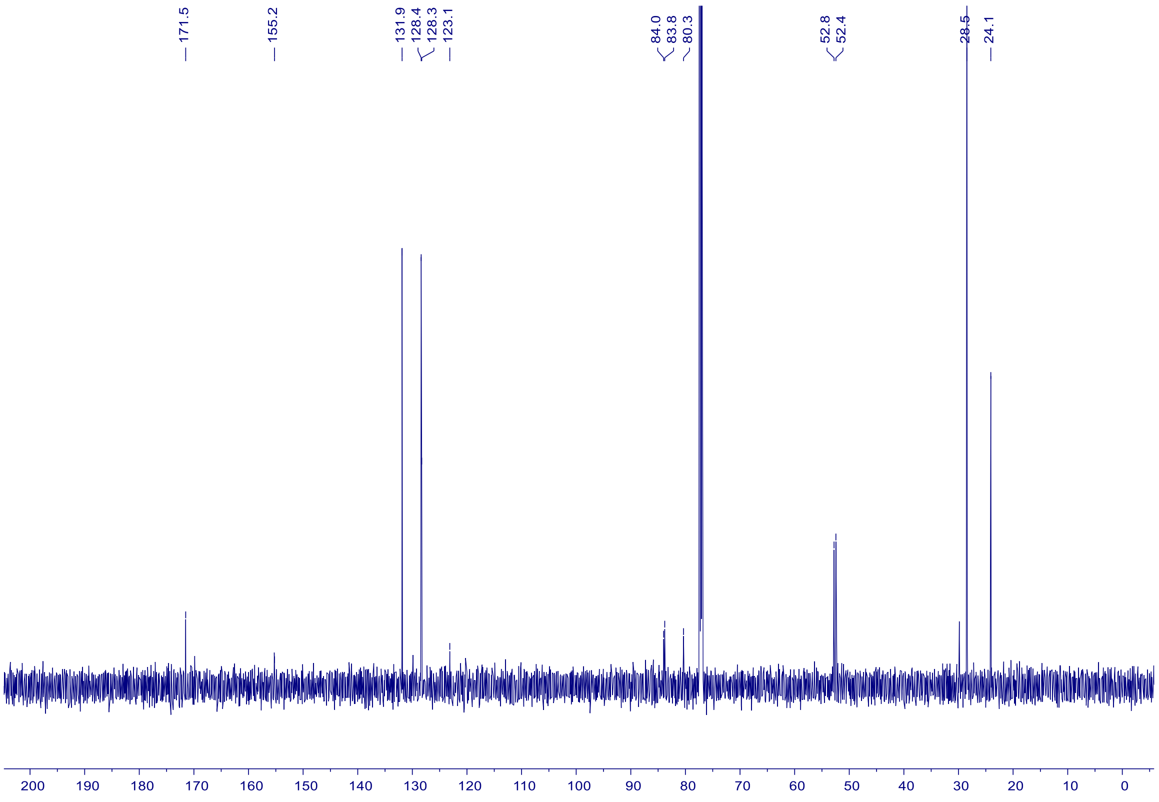

31 – ^{1}H NMR (600 MHz, $CDCl_3$)

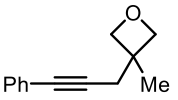

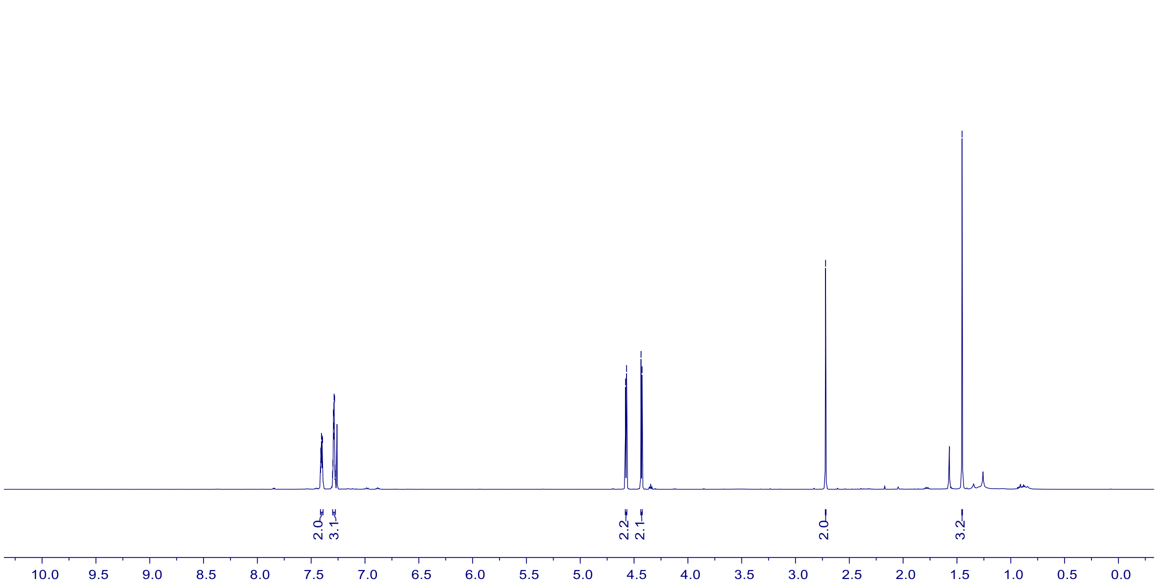

31 – ^{13}C NMR (151 MHz, $CDCl_3$)

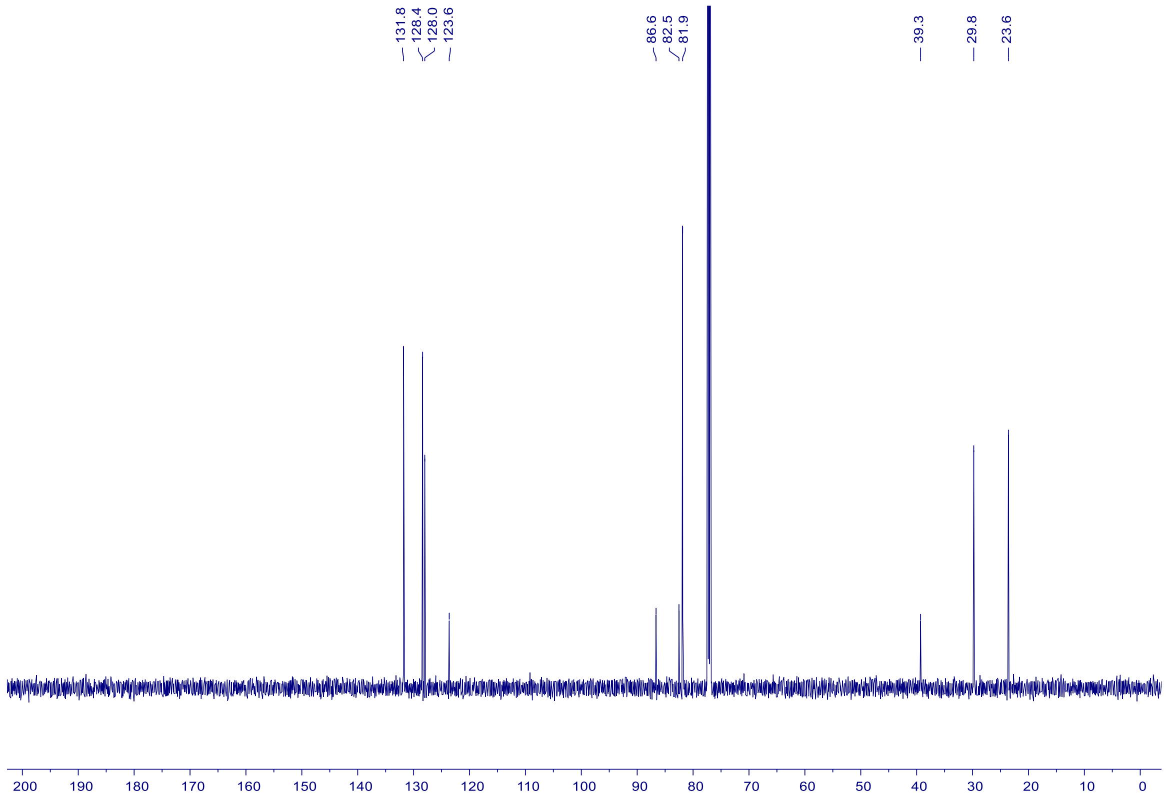

35 – ^{1}H NMR (600 MHz, $CDCl_3$)

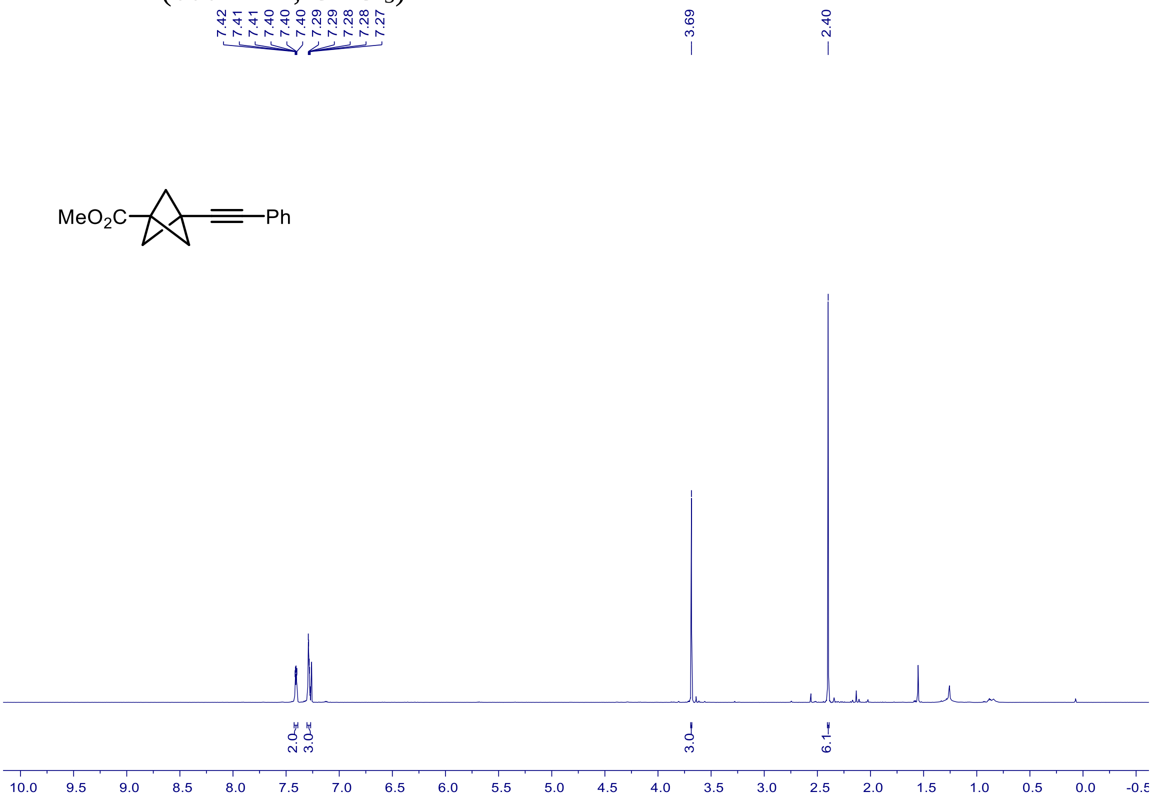

35 – ^{13}C NMR (151 MHz, $CDCl_3$)

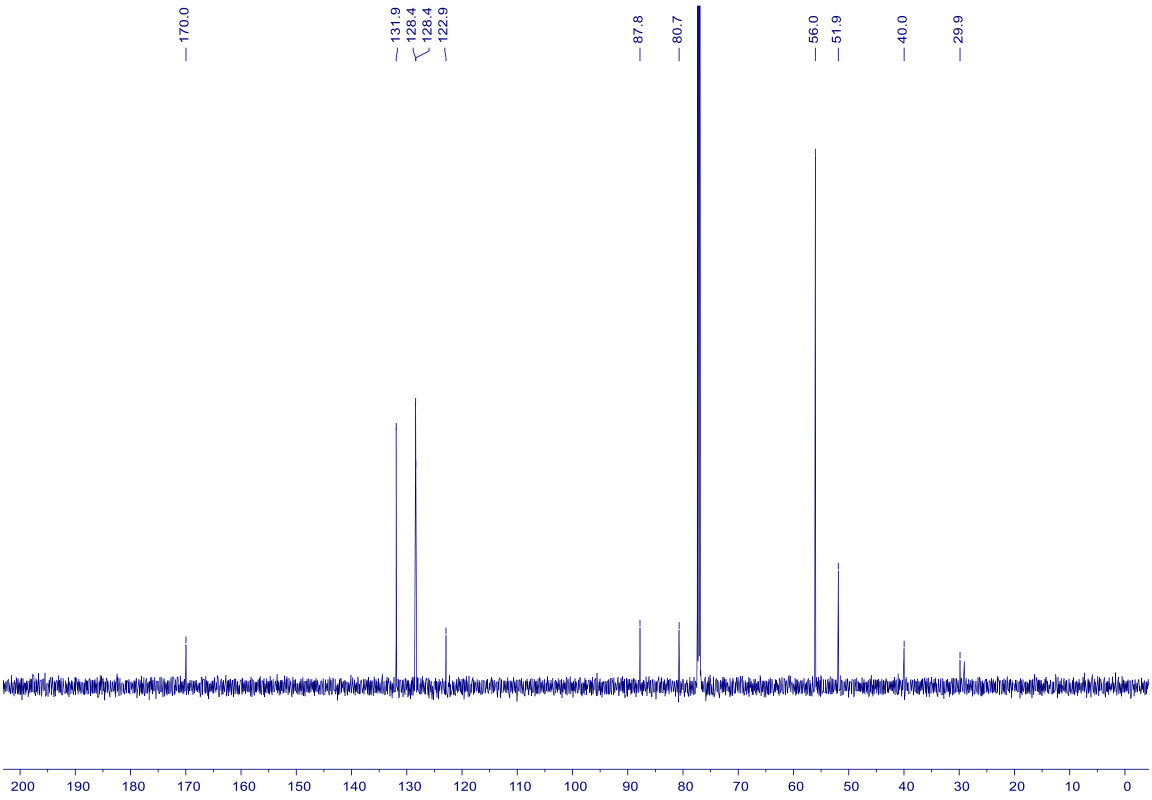

36 – ^{1}H NMR (600 MHz, $CDCl_3$)

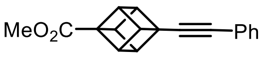

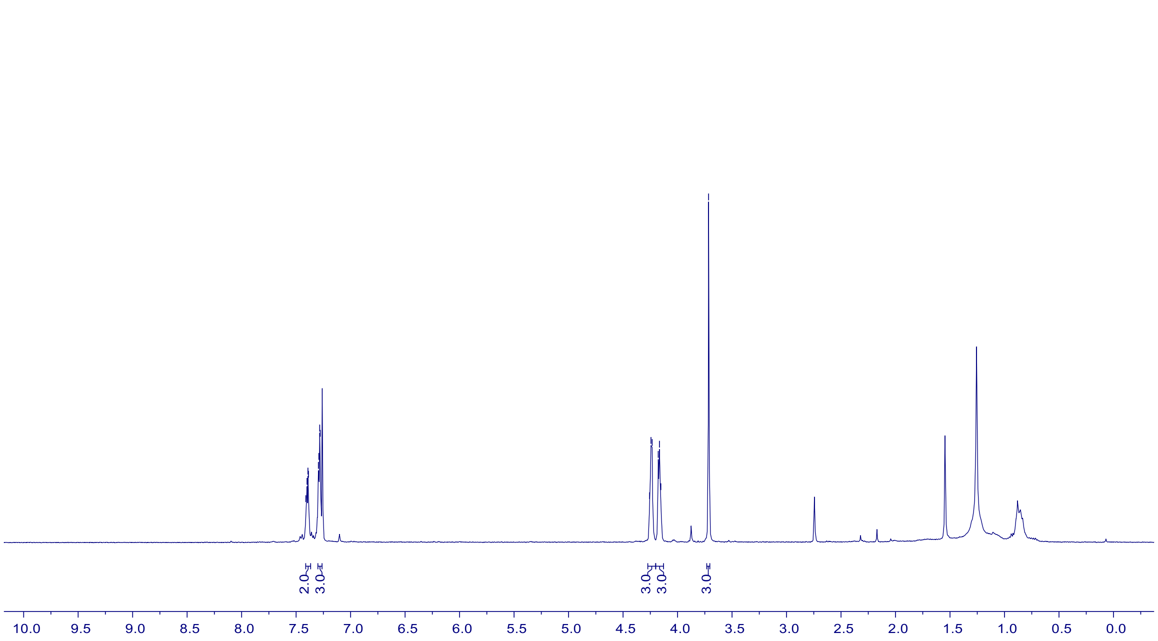

36 – ^{13}C NMR (151 MHz, $CDCl_3$)

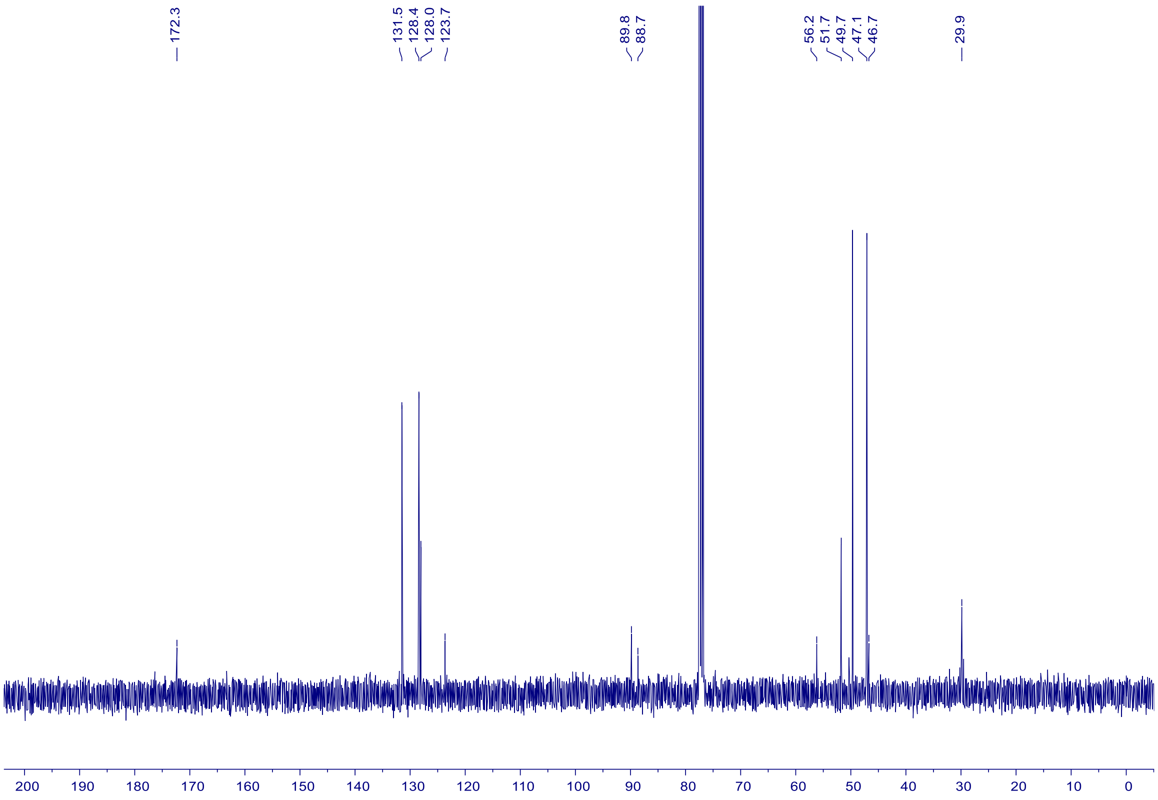

37 – ^{1}H NMR (600 MHz, $CDCl_3$)

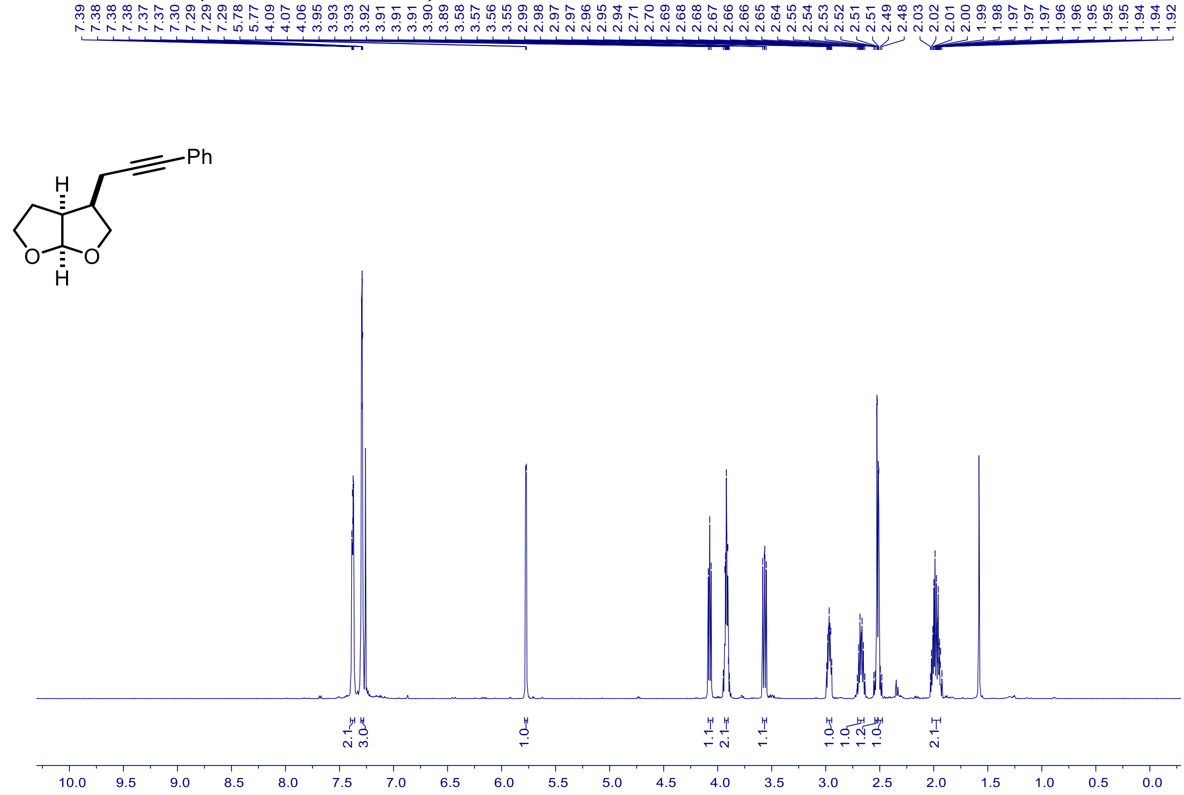

37 – ^{13}C NMR (151 MHz, $CDCl_3$)

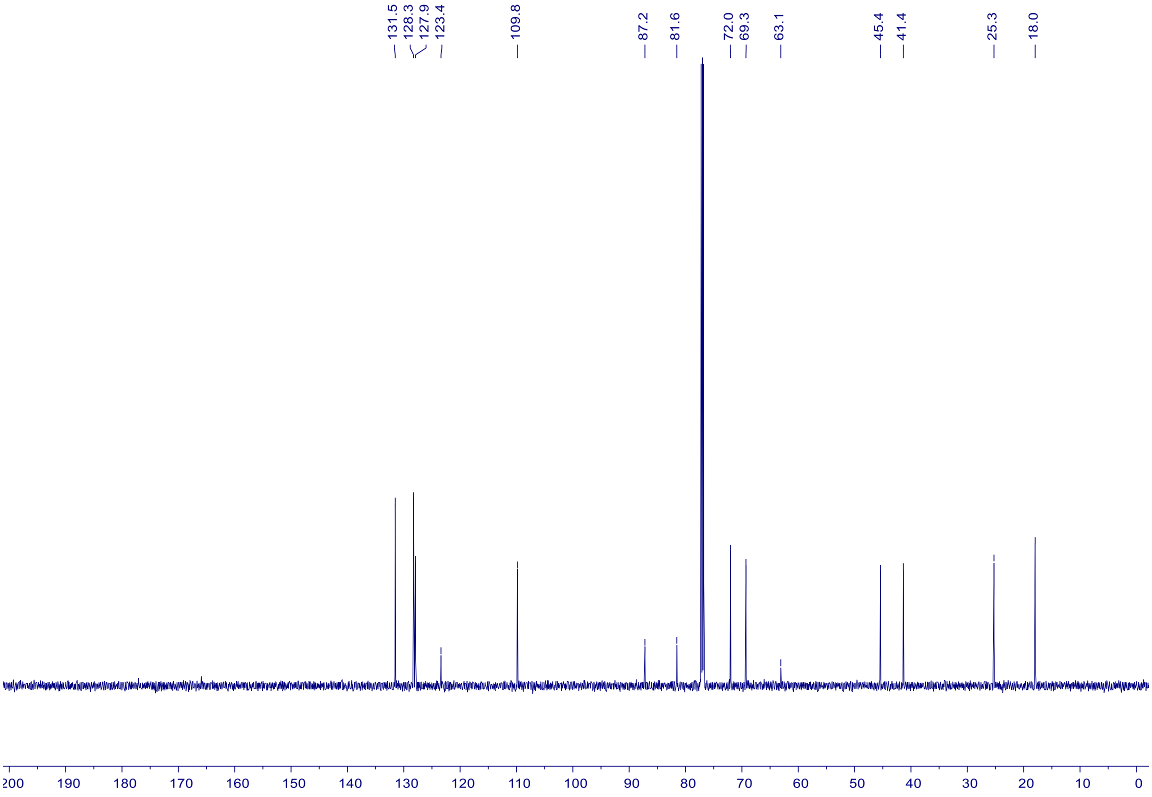

37 – 2D COSY (600 MHz, $CDCl_3$)

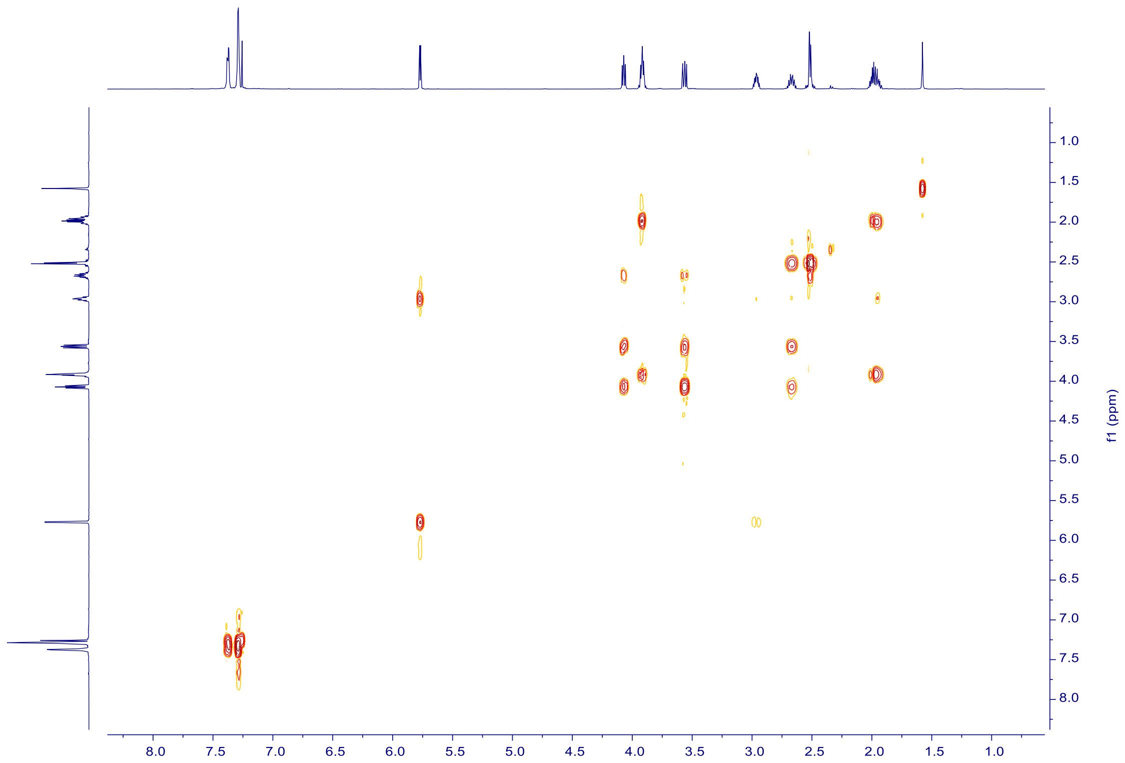

37 – 2D HSQC (151 MHz, $CDCl_3$)

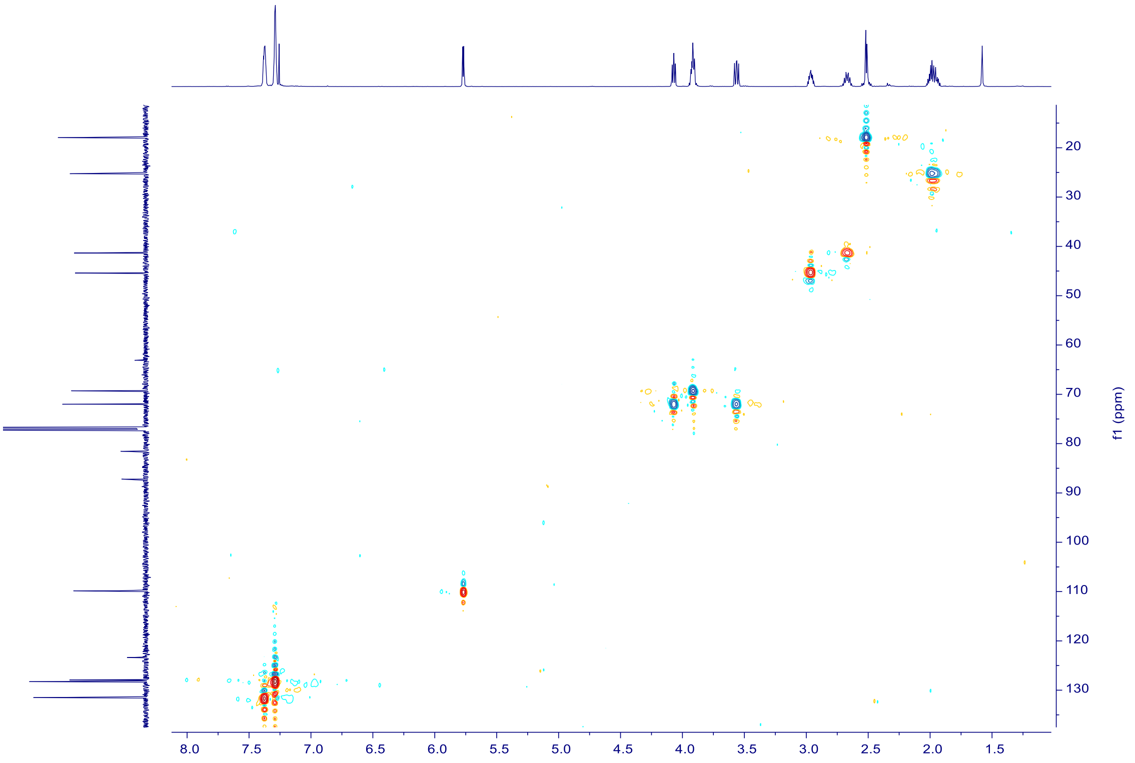

37 –2D HMBC (151 MHz, $CDCl_3$)

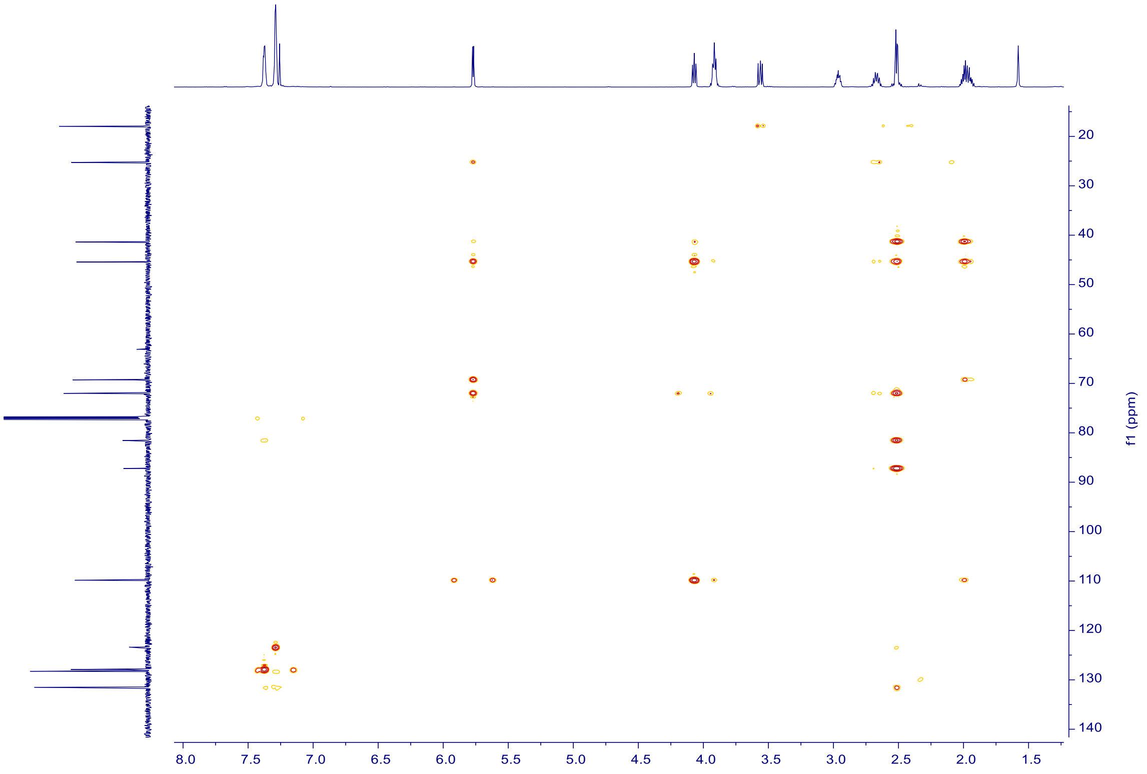

37 – Quantitative NOE (600 MHz, $CDCl_3$)

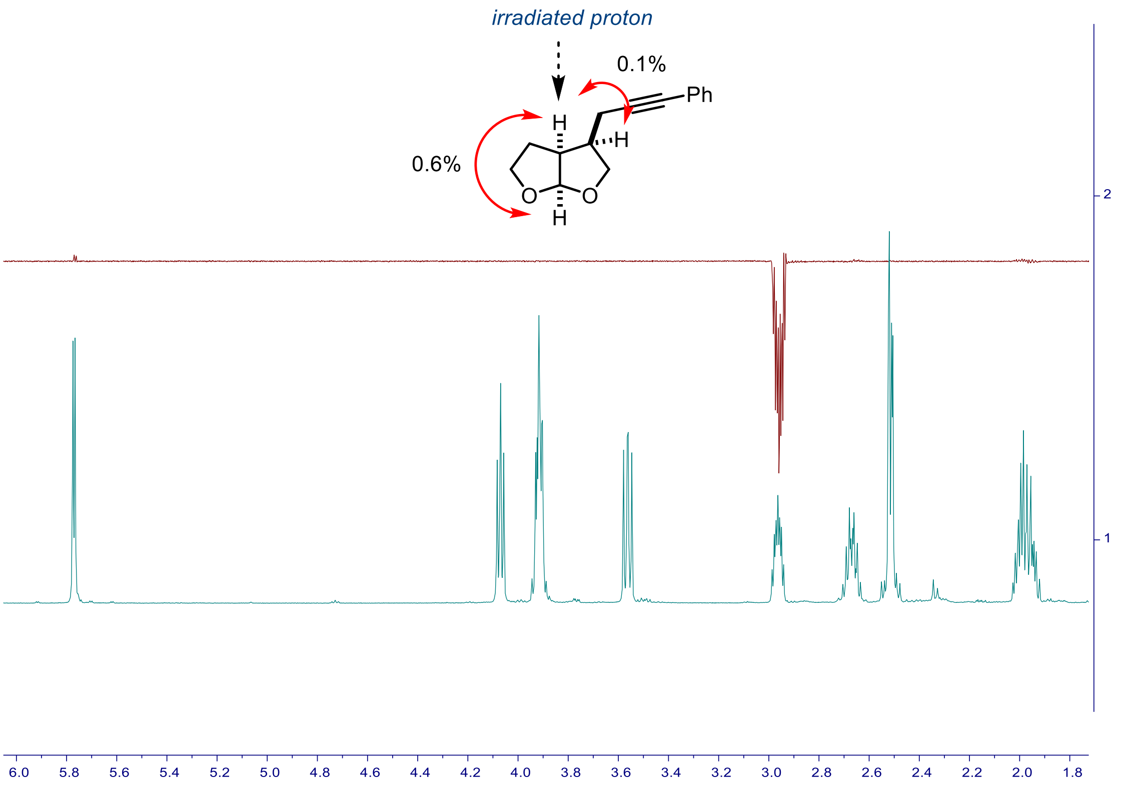

37 – Quantitative NOE (600 MHz, $CDCl_3$)

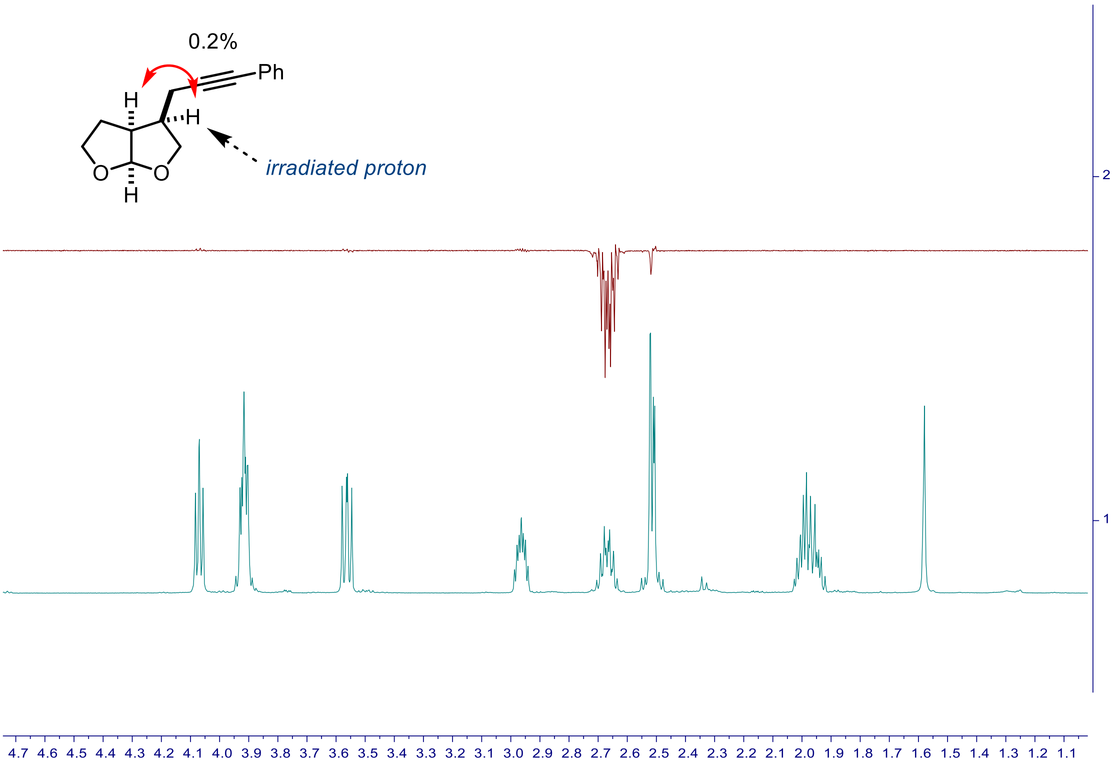

37 – Quantitative NOE (600 MHz, $CDCl_3$)

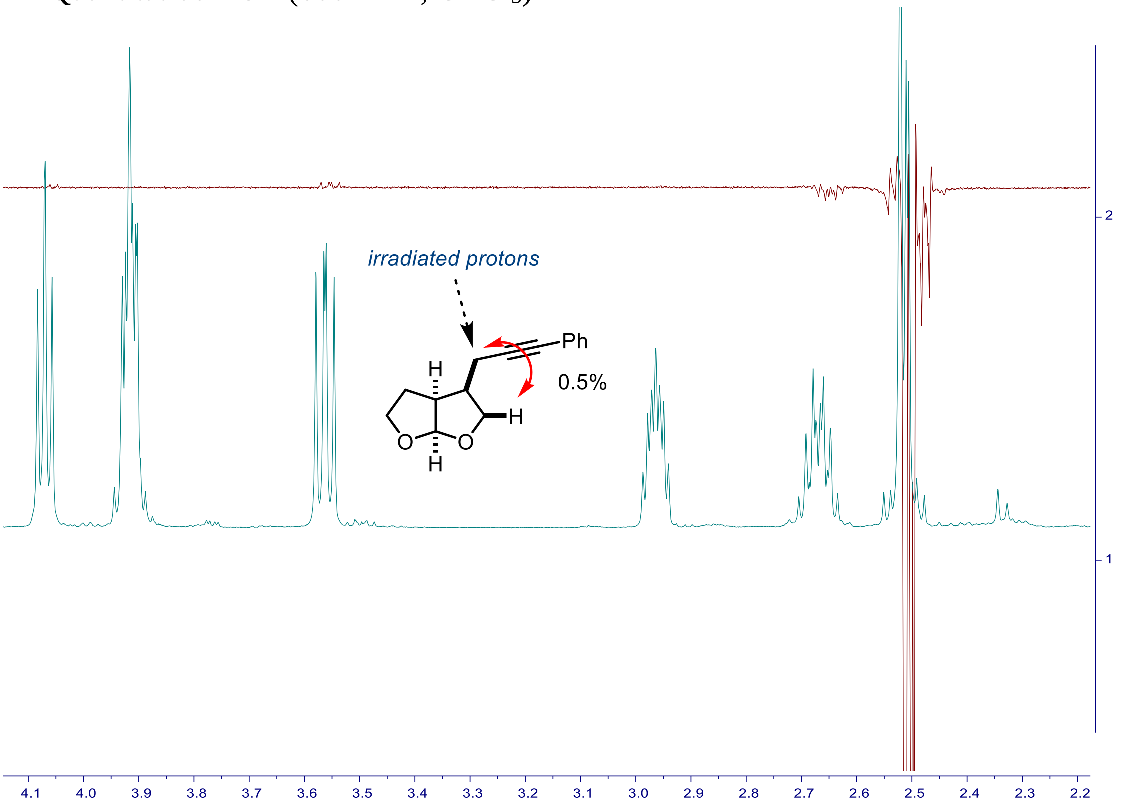

38 – ^{1}H NMR (600 MHz, $CDCl_3$)

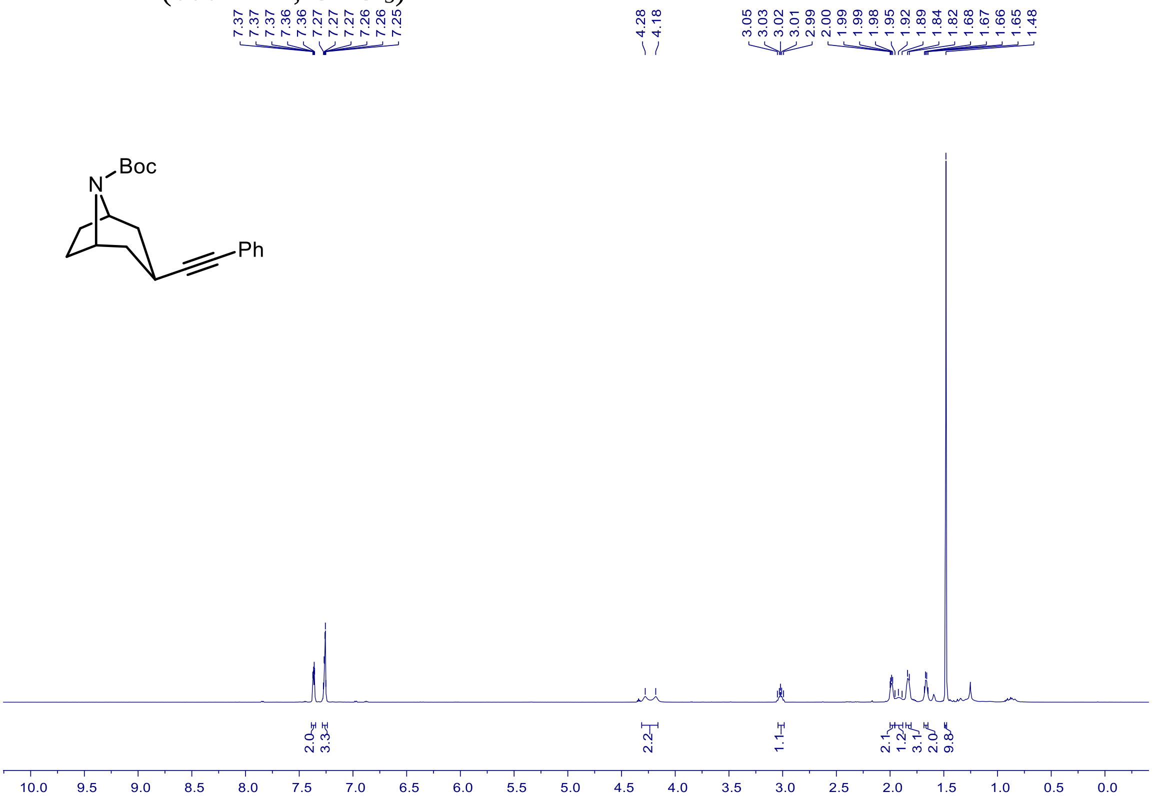

38 – ^{13}C NMR (151 MHz, $CDCl_3$)

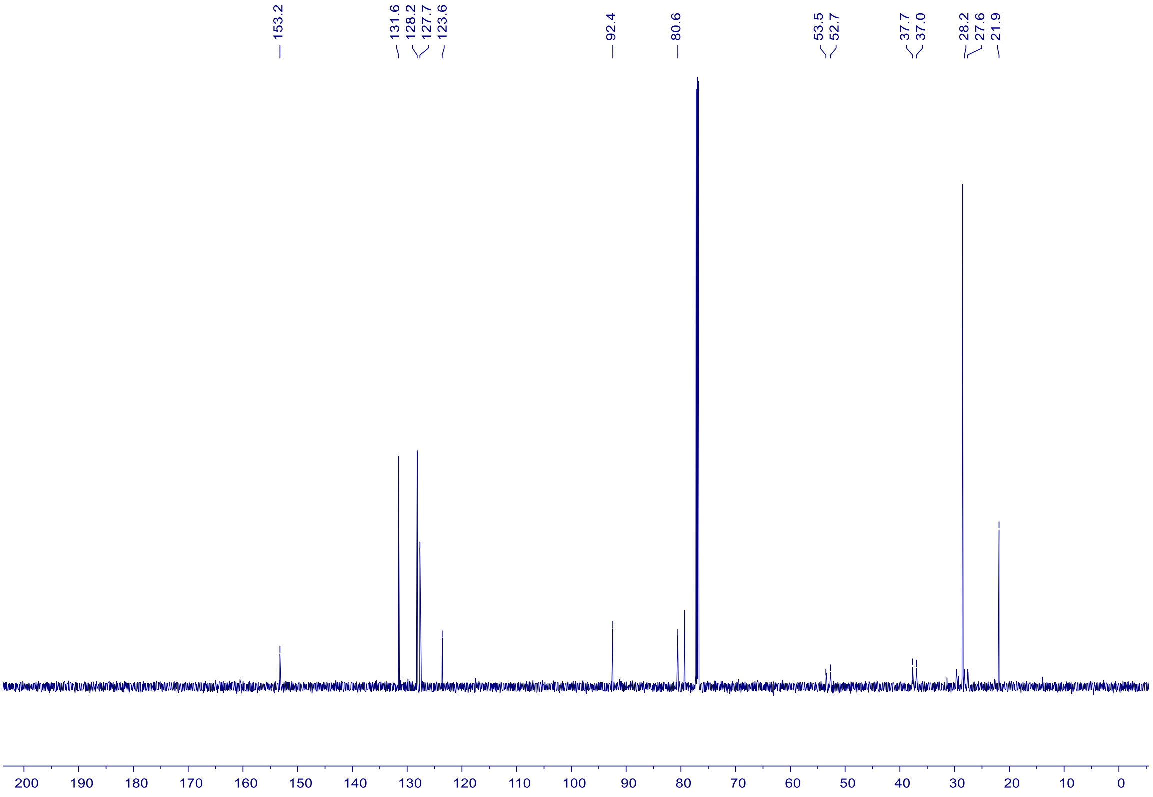

39 – ^{1}H NMR (600 MHz, $CDCl_3$)

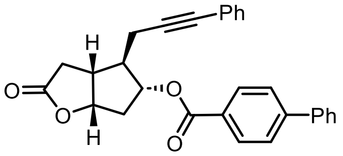

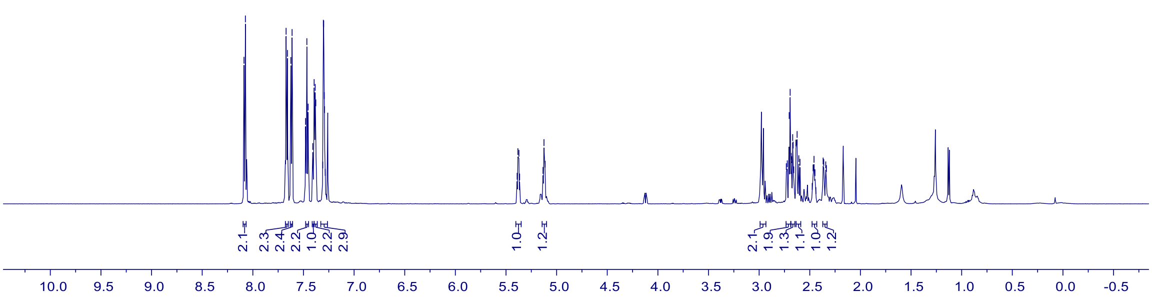

39 – ^{13}C NMR (151 MHz, $CDCl_3$)

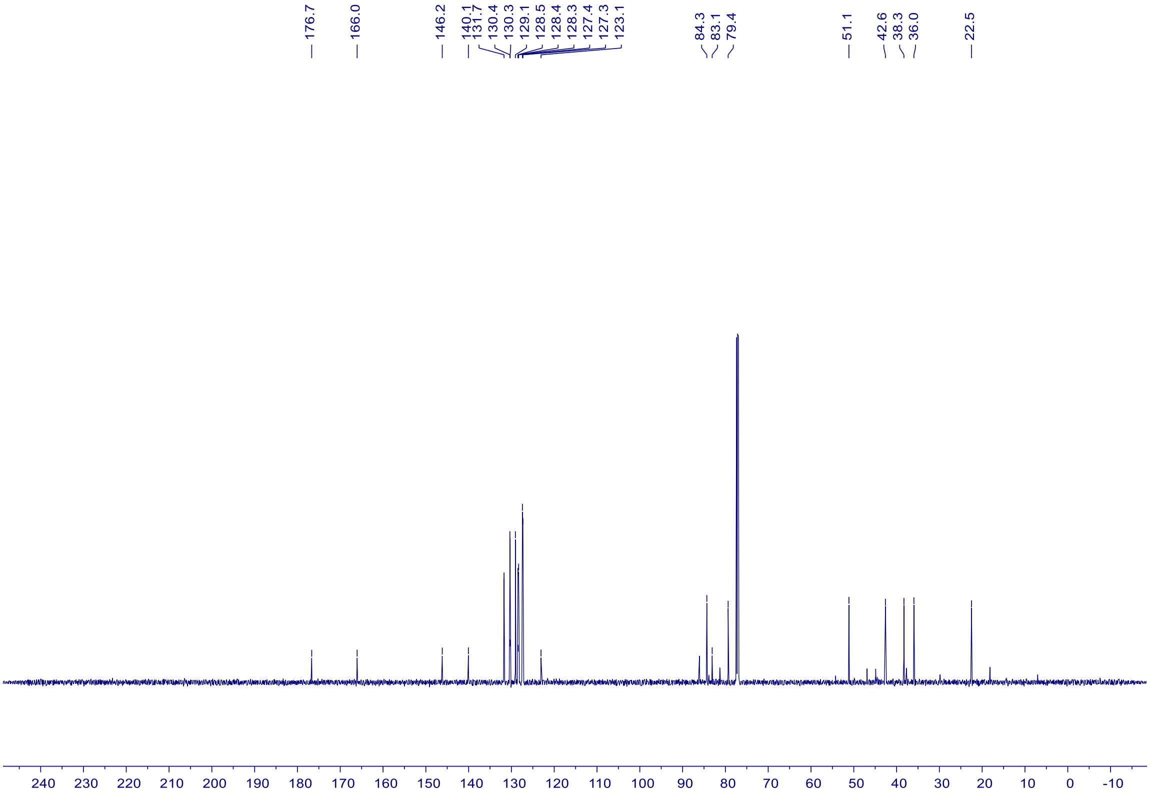

40 – ^{1}H NMR (600 MHz, CDCl$_3$)

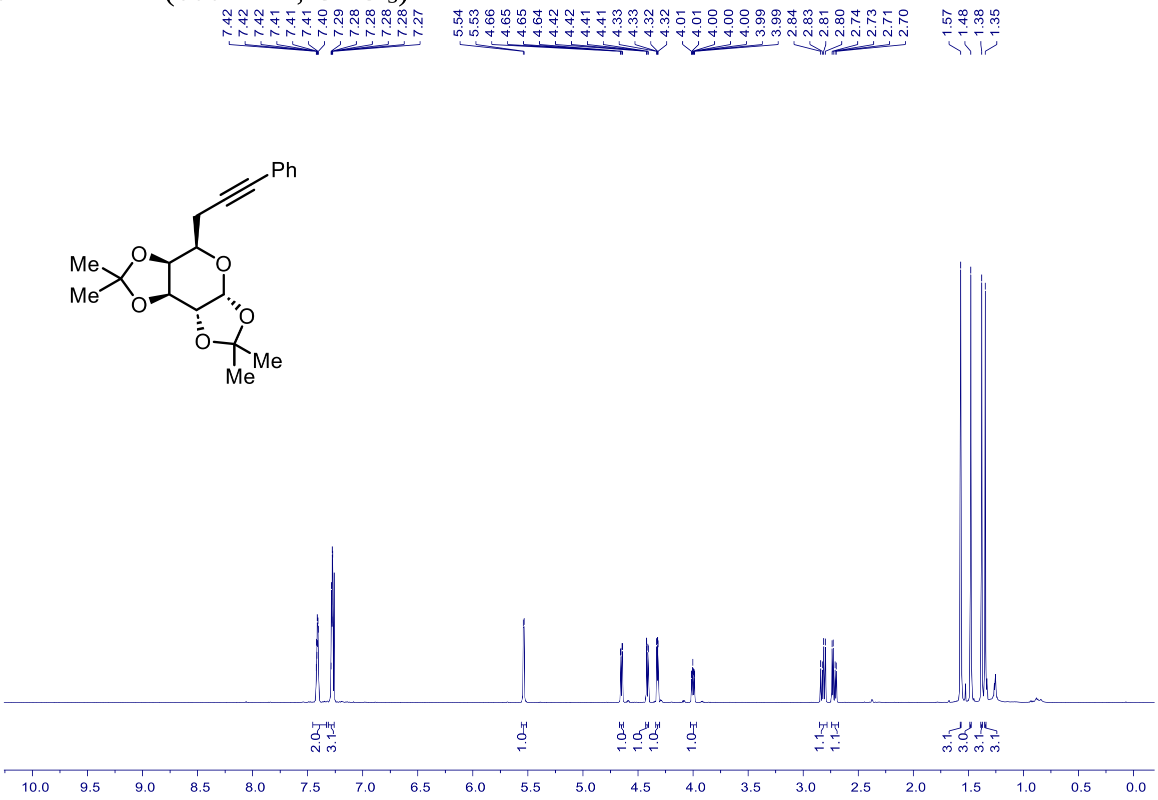

40 – ^{13}C NMR (151 MHz, CDCl$_3$)

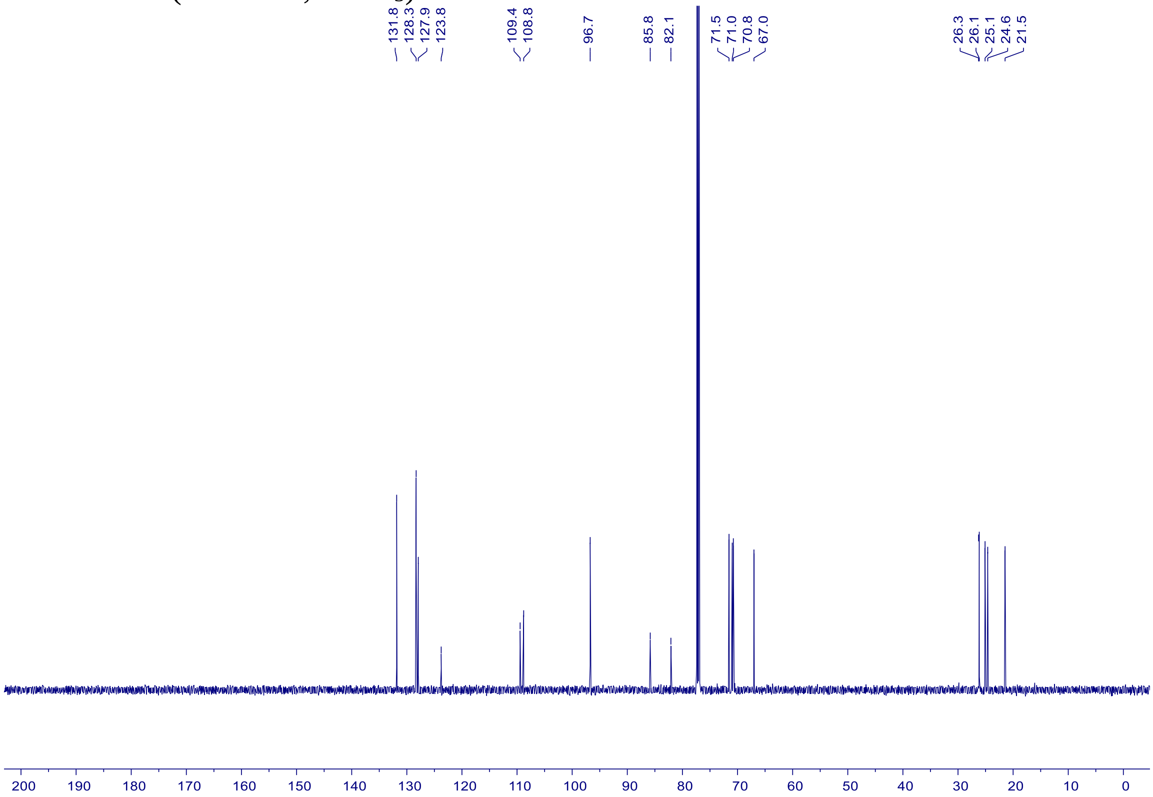

41 – ^{1}H NMR (600 MHz, $CDCl_3$)

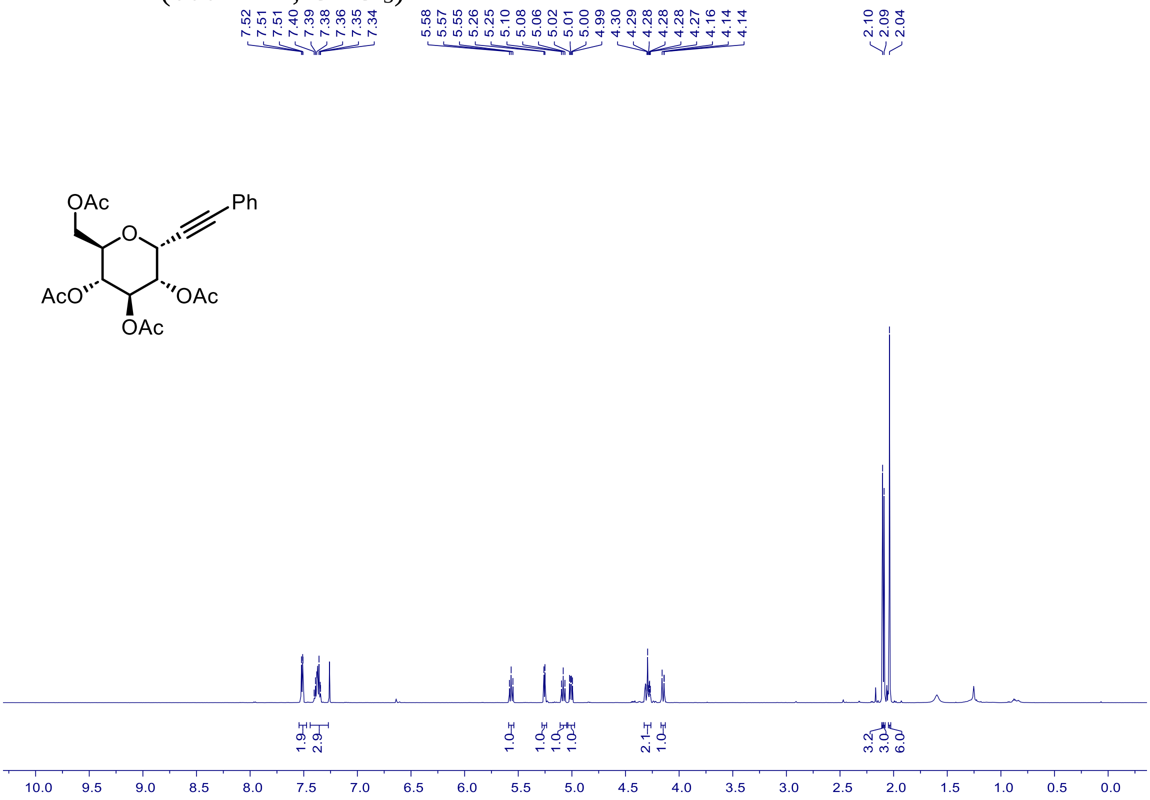

41 – ^{13}C NMR (151 MHz, $CDCl_3$)

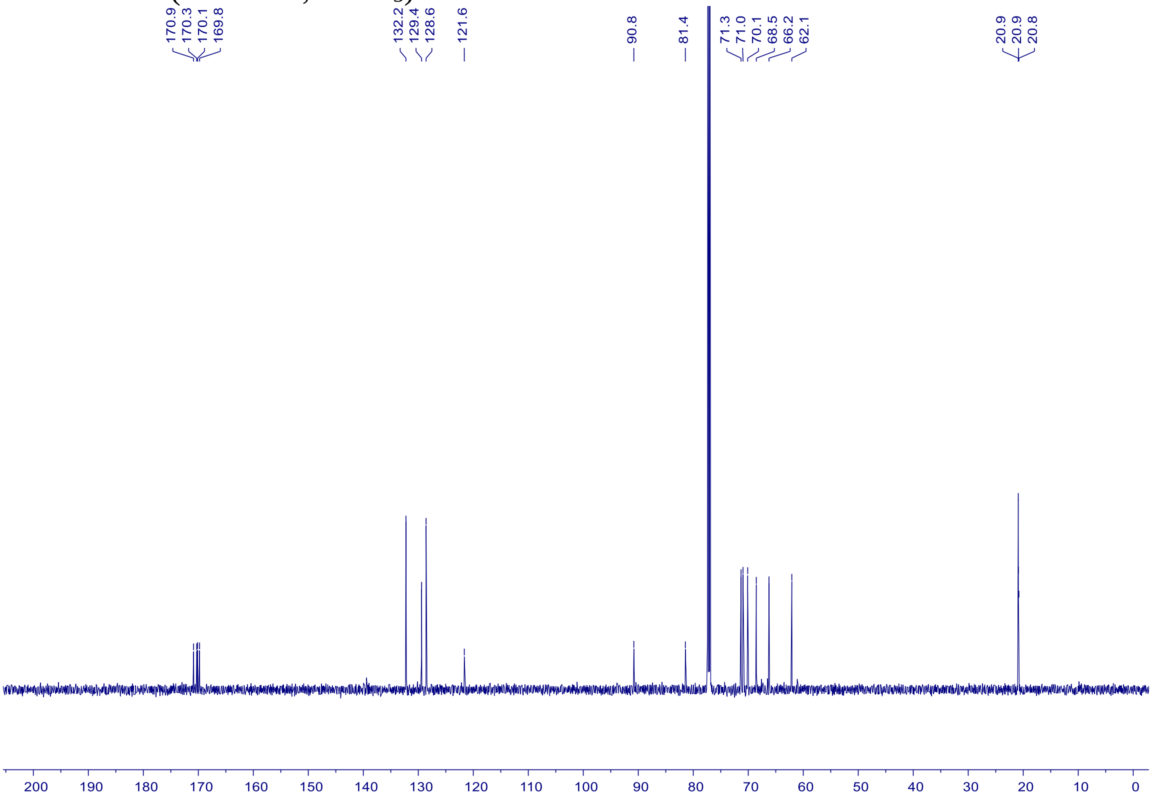

41 – 2D NOESY (600 MHz, $CDCl_3$)

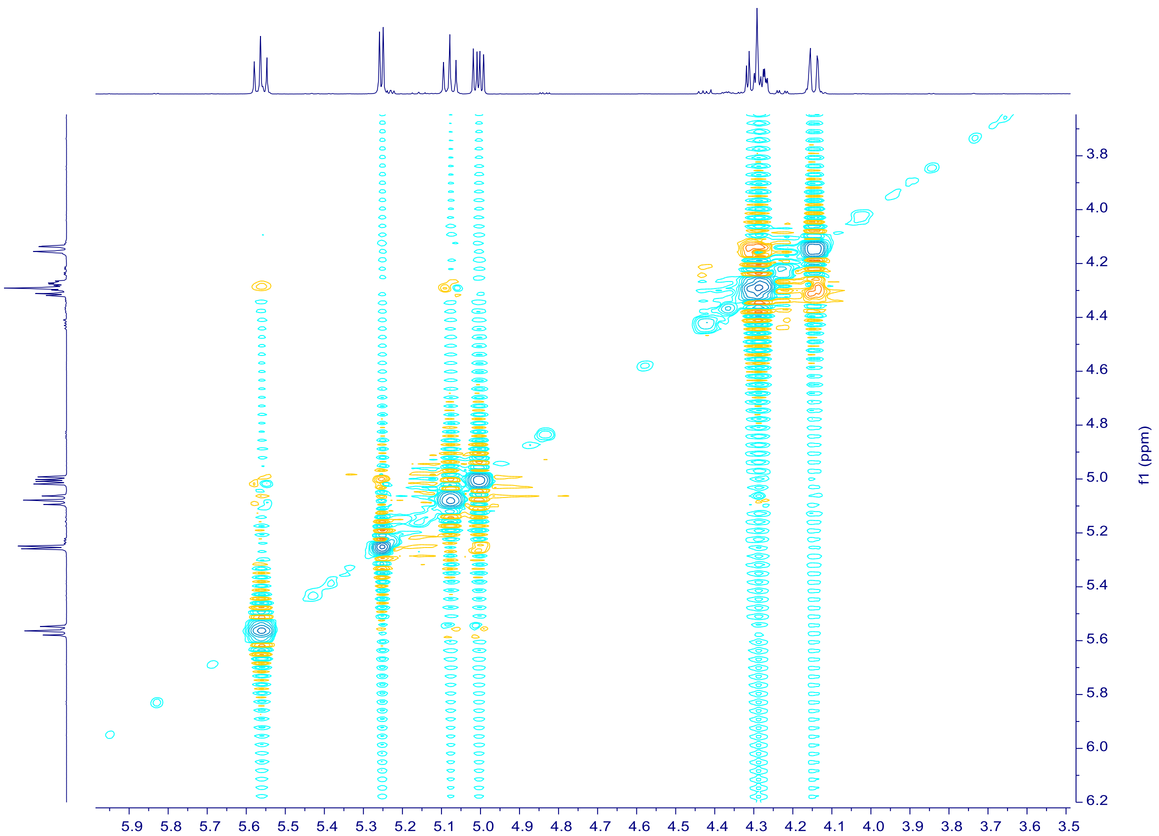

42 – ^{1}H NMR (600 MHz, $CDCl_3$)

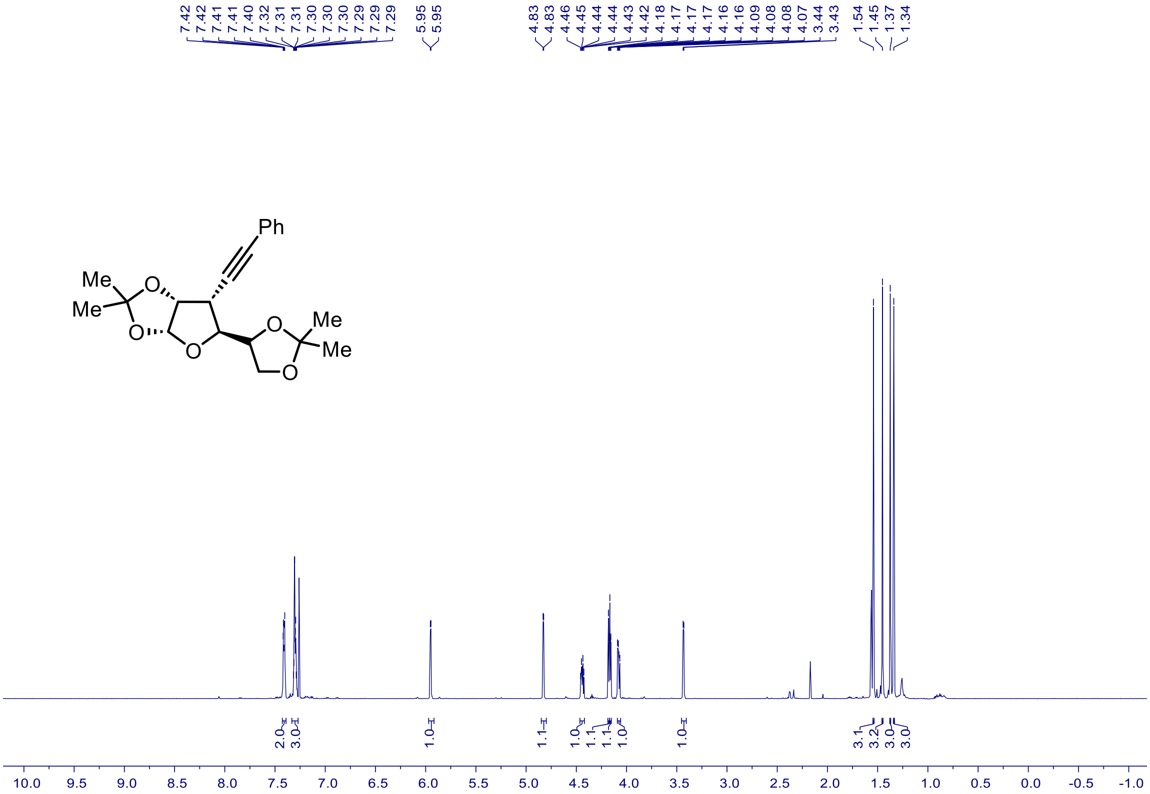

42 – ^{13}C NMR (151 MHz, $CDCl_3$)

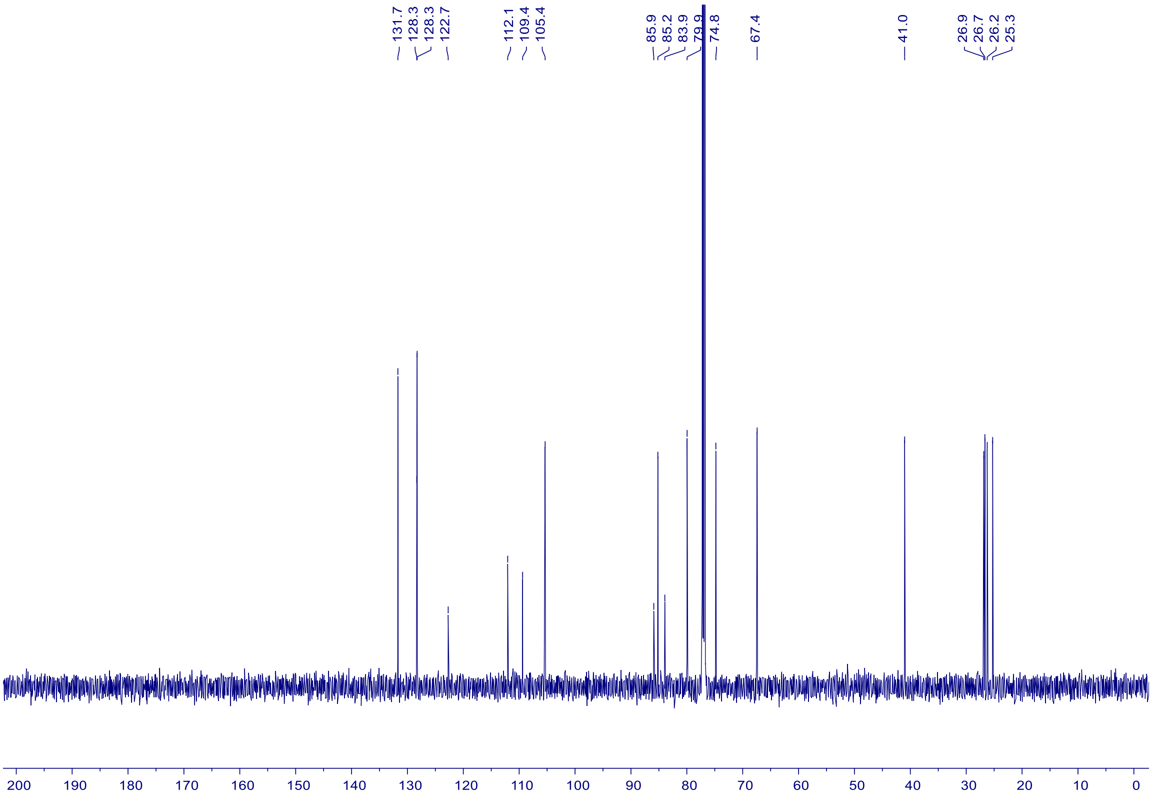

42 – Quantitative NOE (600 MHz, $CDCl_3$)

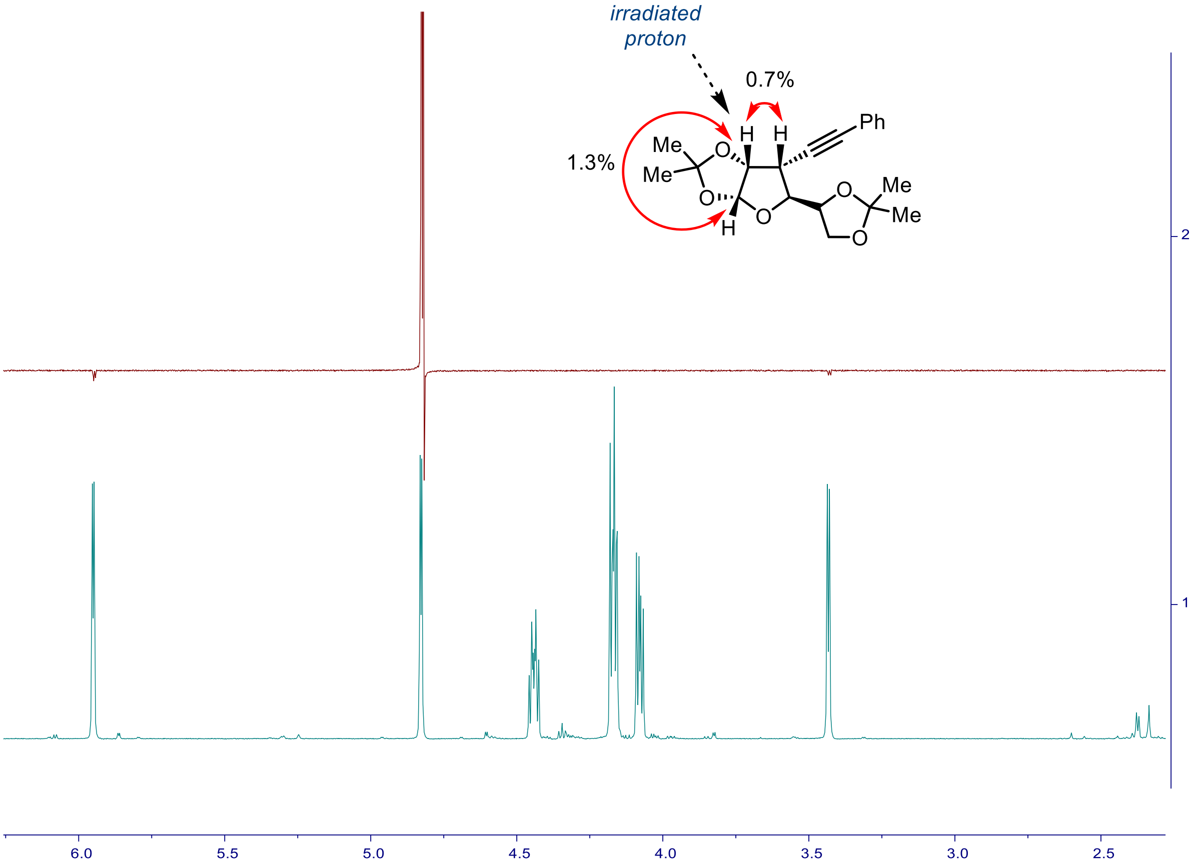

43 – ^{1}H NMR (600 MHz, $CDCl_3$)

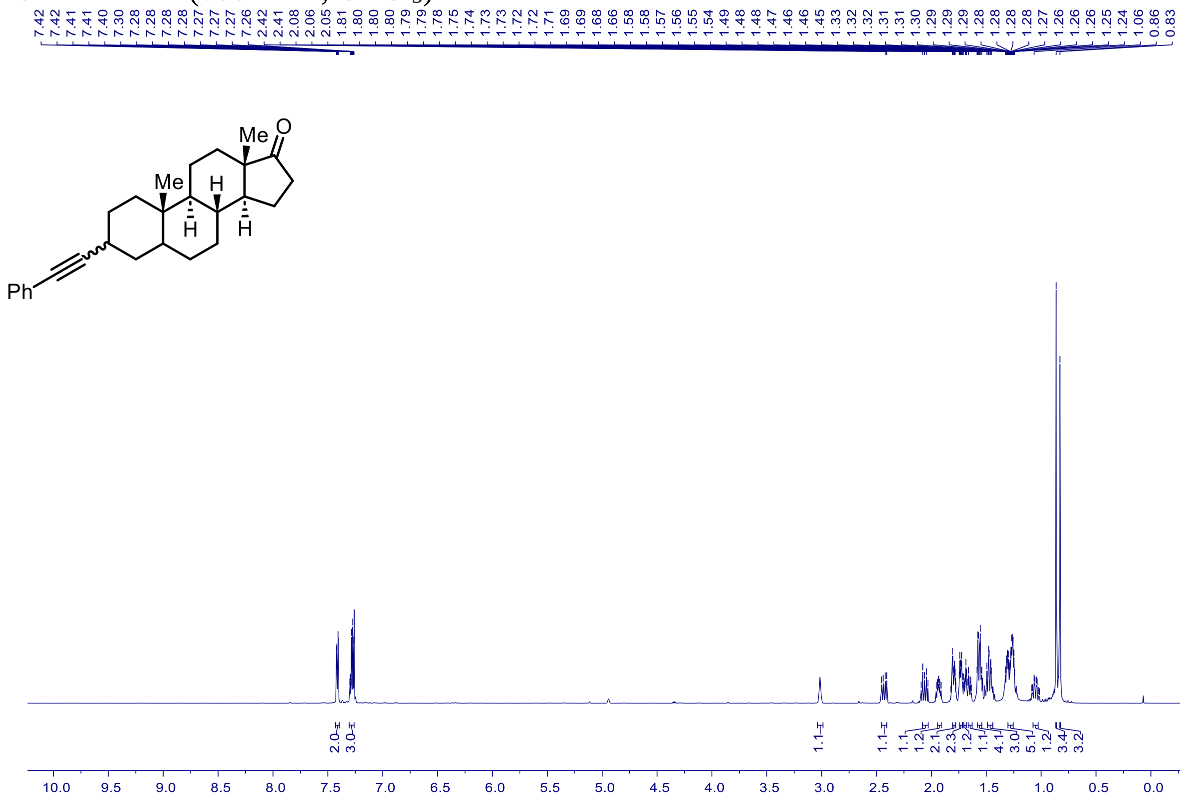

43 – ^{13}C NMR (151 MHz, $CDCl_3$)

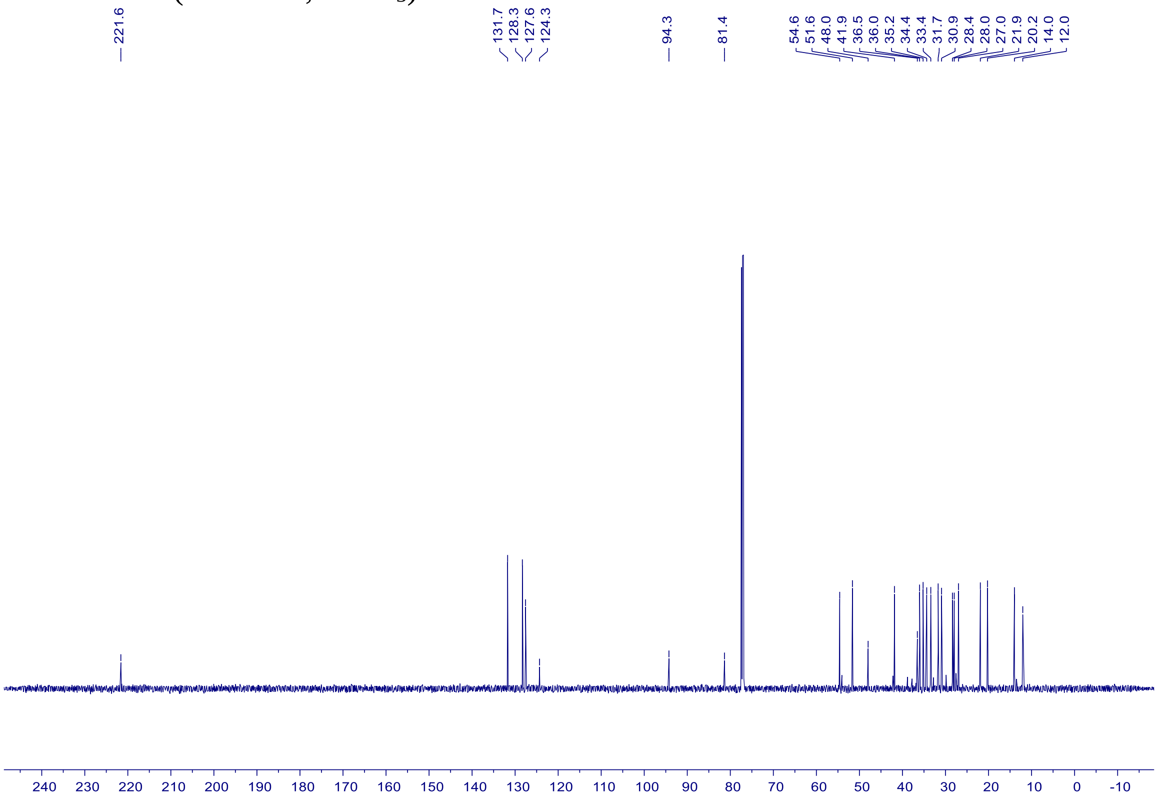

43’ – ^{1}H NMR (600 MHz, $CDCl_3$)

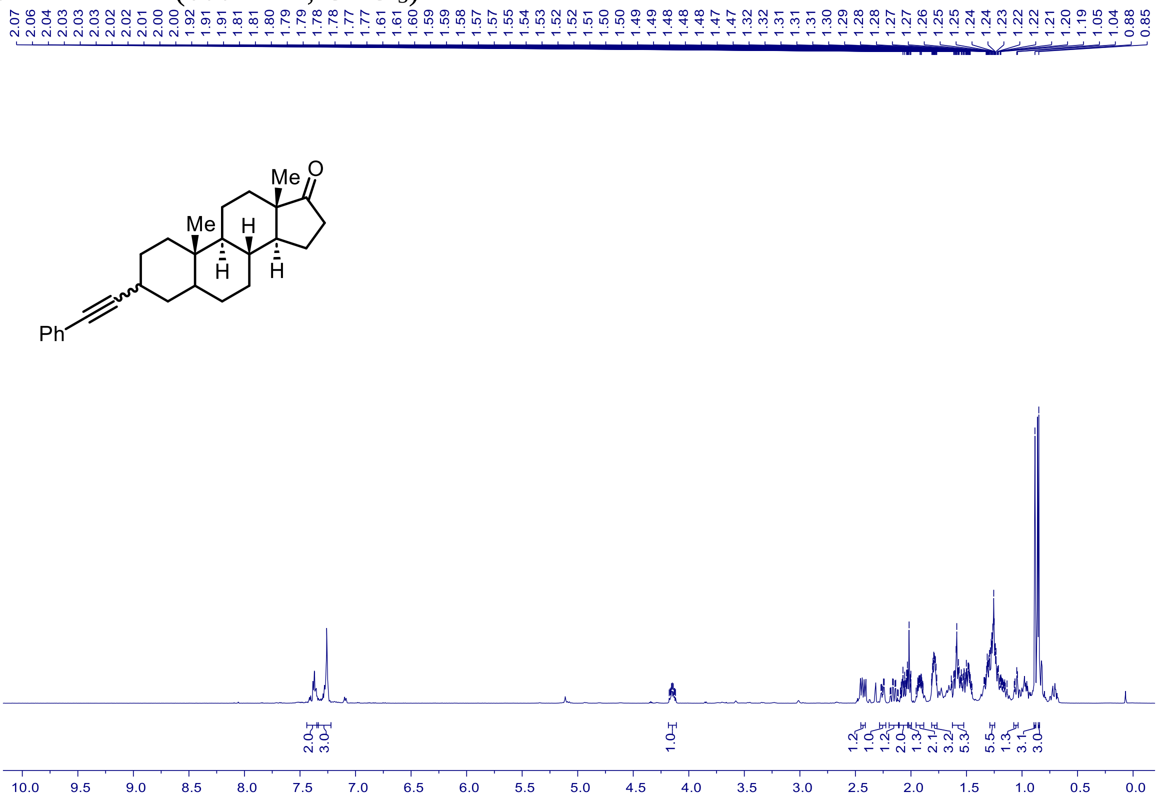

43’ – ^{13}C NMR (151 MHz, $CDCl_3$)

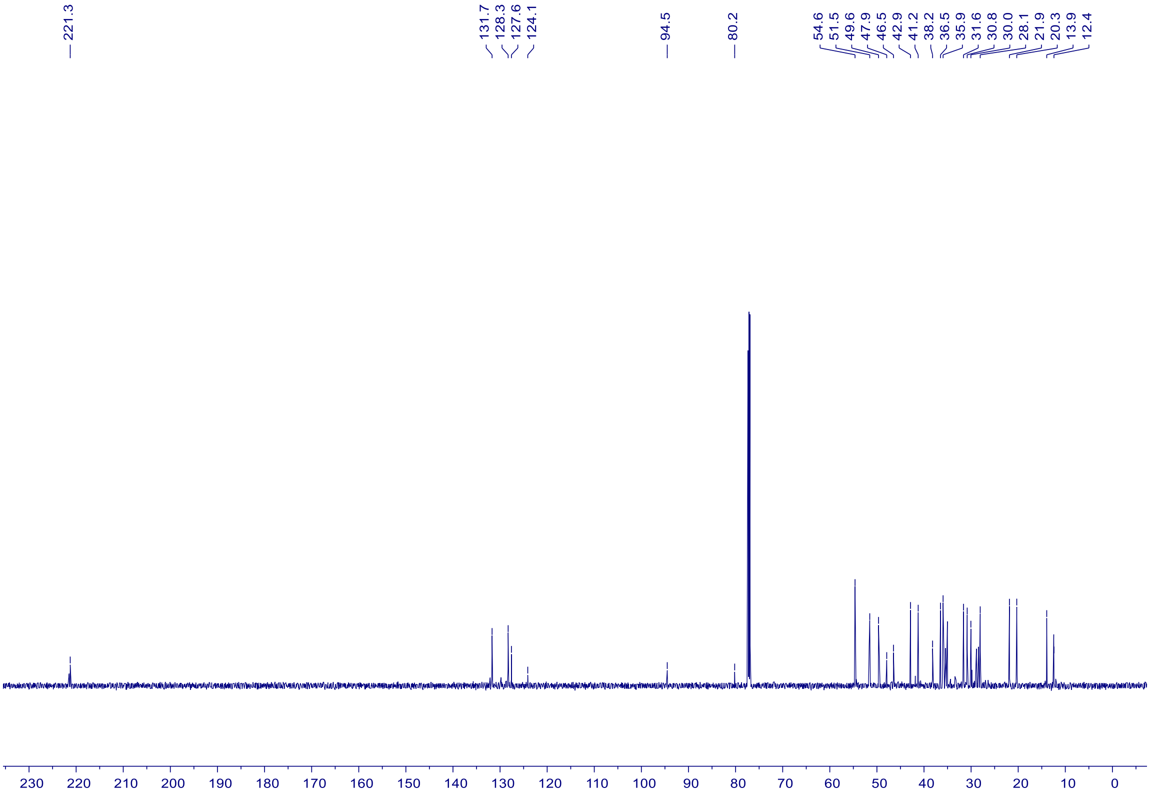

44 – ^{1}H NMR (600 MHz, CDCl$_3$)

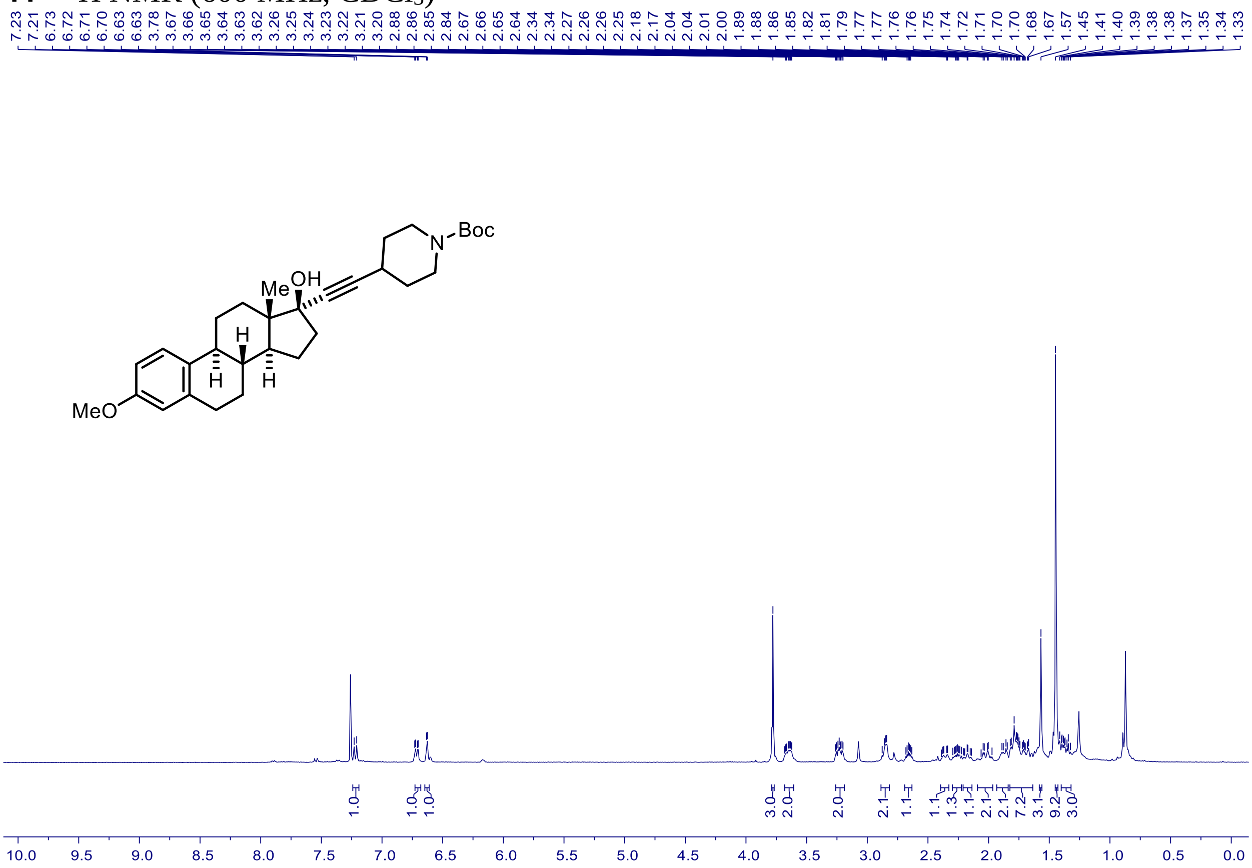

44 – ^{13}C NMR (151 MHz, CDCl$_3$)

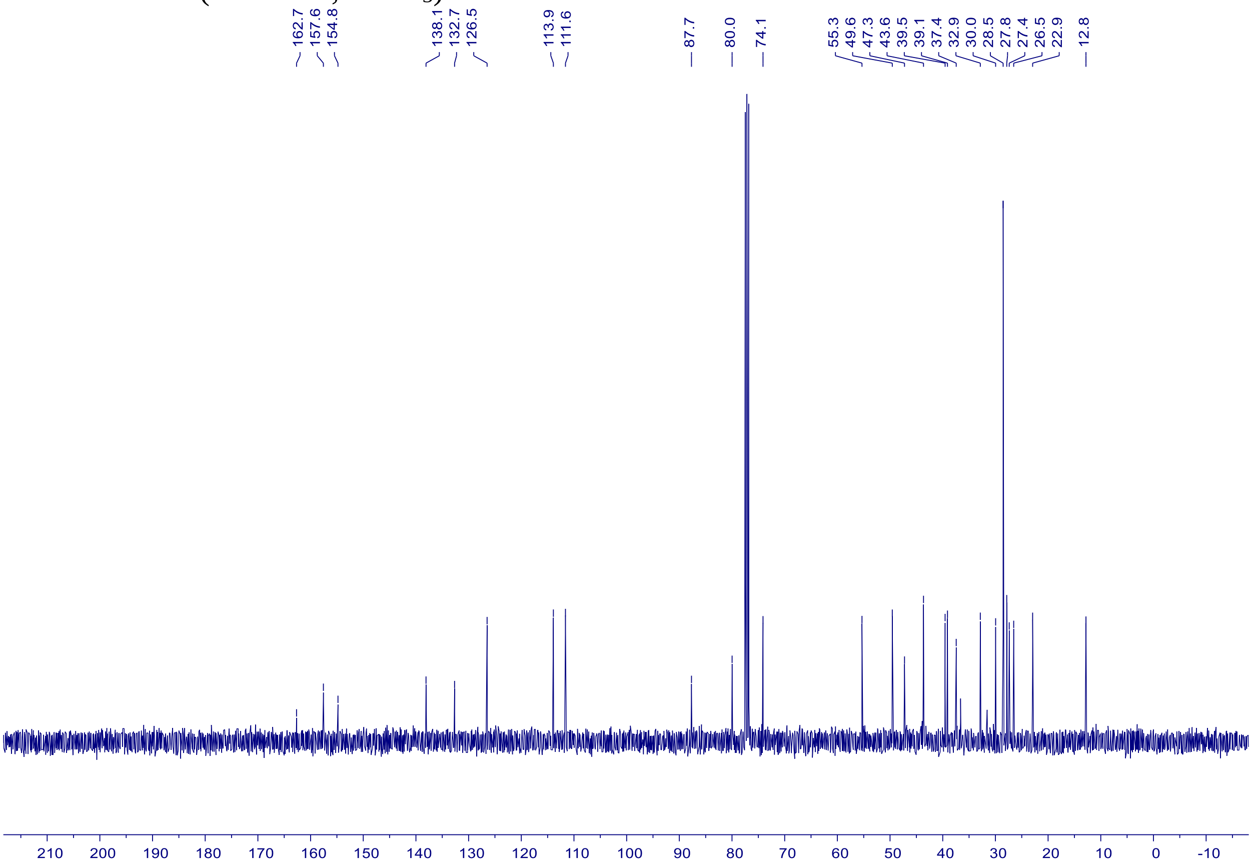

10. References

[1] (a) Z. Zhang, M. J. Tilby, and D. Leonori, *Nat. Synth. 2024*, DOI: 10.1038/s44160-024-00587-5; (b) Z. Zhang, L. Poletti and D. Leonori, *J. Am. Chem. Soc. 2024*, **146**, 22424–22430.

[2] J. Meesin, P. Katrun, C. Pareseecharoen, M. Pohmakotr, V. Reutrakul, D. Soorukram, and C. Kuhakarn, *J. Org. Chem. 2016*, **81**, 2744–2752.

[3] Z.-Y. Mo, Y.-Z. Zhang, G.-B. Huang, X.-Y. Wang, Y.-M. Pan, and H.-T. Tang, *Adv. Synth. Catal. 2020*, **362**, 2160–2167.

[4] K. Cao, N. Zhang, L. Lin, Q. Shen, H. Jiang, and J. Li, *Adv. Synth. Catal. 2024*, **366**, 207–213.

[5] I. Tanaka, M. Sawamura, and Y. Shimizu, *Org. Lett. 2022*, **24**, 520–524**.**

[6] L. Wanga, W. Weia, D. Yanga, H. Cuia, H. Yueb, and H. Wang, *Tetrahedron Lett. 2017*, **58**, 4799–4802.

[7] C. Savarin, J. Srogl, and L. S. Liebeskind, *Org. Lett. 2001*, **3**, 91–93.

[8] M. Ye, M. Hou, Y. Wang, X. Ma, K. Yang, and Q. Song, *Org. Lett. 2023*, **25**, 1787–1792.

[9] A. Rani, T. Amit, K. Rizwana, R. A. Avishek, K. Jha, and V. R. Yatham, *Org. Lett. 2022*, **24**, 5186–5191.

[10] Y. Shen, B. Huang, J. Zheng, C. Lin, Y. Liu, and S. Cui, *Org. Lett. 2017*, **19**, 1744–1747.

[11] F. Zhurkin, W. Parisot, and G. Lefèvre, *Adv. Synth. Catal. 2024*, **366**, 1782–1787.

[12] J. M. Halford-McGuff , A. P. McKay , and A. J. B. Watson, *Synlett 2024*, **35**, A–E. DOI: 10.1055/a-2285-0007.

[13] M.-G. Braun, and A. G. Doyle, *J. Am. Chem. Soc. 2013*, **135**, 12990–12993.

[14] B. C. Gorske, C. T. Mbofana, and S. J. Miller, *Org. Lett. 2009*, **11**, 4318–4321.

[15] A. C. Breman, A. Ruiz-Olalla, J. H. van Maarseveen, S. Ingemann, and H. Hiemstra, *Eur. J. Org. Chem. 2014*, 7413–7425.

[16] P. A. Wender, F. Inagaki, M. Pfaffenbach, and M. C. Stevens, *Org. Lett. 2014*, **16**, 2923–2925.

[17] M. C. B. Legault, C. S. McKay, J. Moran, M. A. Lafreniere, and J. P. Pezacki, *Tetrahedron Lett. 2012*, **53**, 5663–5666.

[18] X. Ma, L. Wang, X. Meng, W. Li, Q. Wang, Y. Gua, and L. Qiua, *Org. Biomol. Chem., 2023*, **21**, 6693–6696.

[19] K. B. Pal, E. M. D. Tommaso, A. K. Inge, and B. Olofsson, *Angew. Chem. Int. Ed. 2023*, **62**, e202301368.

[20] H. Zhao, V. D. Cuomo, J. A. Rossi-Ashton, and D. J. Procter, *Chem 2024,* **10**, 1240–1251.
[21] B. D. Dherange, M. Yuan, C. B. Kelly, C. A. Reiher, C. Grosanu, K. J. Berger, O. Gutierrez, and M. D. Levin, *J. Am. Chem. Soc. 2023,* **145**, 17–24.
